# Pseudovibriamides from *Pseudovibrio* marine sponge bacteria promote flagellar motility via transcriptional modulation

**DOI:** 10.1128/mbio.03115-24

**Published:** 2024-12-27

**Authors:** Yitao Dai, Vitor Lourenzon, Laura P. Ióca, Dua Al-Smadi, Lydia Arnold, Ian McIntire, Roberto G. S. Berlinck, Alessandra S. Eustáquio

**Affiliations:** 1 Department of Pharmaceutical Sciences and Center for Biomolecular Sciences, College of Pharmacy, University of Illinois Chicago, Chicago, Illinois, USA; 2 Instituto de Química de São Carlos, Universidade de São Paulo, São Carlos, Brazil; Max-Planck-Institut fur terrestrische Mikrobiologie, Marburg, Germany

**Keywords:** flagellar motility, proteobacteria, secondary metabolite, transcriptomics, marine sponge

## Abstract

*Pseudovibrio* α-Proteobacteria have been repeatedly isolated from marine sponges and proposed to be beneficial to the host. Bacterial motility is known to contribute to host colonization. We have previously identified pseudovibriamides A and B, produced in culture by *Pseudovibrio brasiliensis* Ab134, and shown that pseudovibriamide A promotes flagellar motility. Pseudovibriamides are encoded in a hybrid nonribosomal peptide synthetase-polyketide synthase gene cluster that also includes several accessory genes. Pseudovibriamide A is a linear heptapeptide and pseudovibriamide B is a nonadepsipeptide derived from pseudovibriamide A. Here, we define the borders of the pseudovibriamides gene cluster, assign function to biosynthetic genes using reverse genetics, and test the hypothesis that pseudovibriamides impact motility by modulating gene transcription. RNA-sequencing transcriptomic analyses of strains having different compositions of pseudovibriamides suggested that both pseudovibriamides A and B affect genes potentially involved in motility, and that a compensatory mechanism is at play in mutants that produce only pseudovibriamide A, resulting in comparable flagellar motility as the wild type. The data gathered suggest that pseudovibriamides A and B have opposite roles in modulating a subset of genes, with pseudovibriamide B having a primary effect in gene activation, and pseudovibriamide A on inhibition. Finally, we observed many differentially expressed genes (up to 29% of the total gene number) indicating that pseudovibriamides have a global effect on transcription that goes beyond motility.

**IMPORTANCE:**

Marine sponges are found throughout the oceans from tropical coral reefs to polar sea floors, playing crucial roles in marine ecosystems. *Pseudovibrio* bacteria have been proposed to contribute to sponge health. We have previously shown that pseudovibriamides produced by *Pseudovibrio brasiliensis* promote bacterial motility, a behavior that is beneficial to bacterial survival and host colonization. The gene cluster that encodes pseudovibriamide biosynthesis is found in two-thirds of *Pseudovibrio* genomes. This gene cluster is also present in *Pseudomonas* bacteria that interact with terrestrial plants and animals. Here, we first assign functions to pseudovibriamide biosynthetic genes using reverse genetics. We then show that pseudovibriamides play a major role in transcriptional regulation, affecting up to 29% of *P. brasiliensis* genes, including motility genes. Thus, this work gives insights into pseudovibriamide biosynthesis and provides evidence that they are signaling molecules relevant to bacterial motility and to other yet-to-be-identified phenotypes.

## INTRODUCTION

Marine sponges are among the oldest animals on Earth (1). Their filter-feeding capacity contributes to biogeochemical cycling and they are also involved in habitat formation, properties that are critical to marine ecology (2). In a bulk nutrient-depleted environment like the open ocean, colonization on marine sponges provides microbes greater access to nutritional resources and environmental stability (3). Conversely, sponge-associated microbes have nutritional and protective roles, contributing to the animal’s health (4).

Marine sponges develop relationships with numerous bacterial species (5, 6). Among these, *Pseudovibrio* spp. are Gram-negative α-Proteobacteria with high frequency of association with marine sponges and have been proposed to contribute to sponge health (7). The presence of *Pseudovibrio* was also confirmed in larvae of a marine sponge suggesting this genus could be a vertically transmitted symbiont (8).

Metabolites produced by microbes are important in the establishment and maintenance of host-microbe associations and in holobiont homeostasis. *Pseudovibrio* bacteria, for example, produce antimicrobials which could ward off pathogens (7). Moreover, flagellar motility—swimming and swarming—has been shown to be important for host colonization (9). Bacterial metabolites such as surfactants are known to facilitate swarming motility (9).

We have previously reported a link between a biosynthetic gene cluster (BGC) in *Pseudovibrio brasiliensis* Ab134 and its flagellar motility (10). Strain Ab134 was isolated from marine sponge *Arenosclera brasiliensis* (11). The BGC, which we termed *Pseudovibrio* and *Pseudomonas* non-ribosomal peptide (*ppp*), is present in 2/3 of sequenced *Pseudovibrio* genomes, and sporadically found in *Pseudomonas* γ-Proteobacteria known to interact with terrestrial plants and insects (10). Moreover, a *ppp*-like BGC was recently reported from *Microbulbifer* γ-Proteobacteria likewise isolated from marine sponges (12). Related natural products detoxins, rimosamides, and chitinimides have been isolated from soil bacteria (13–16).

**Fig 1:**
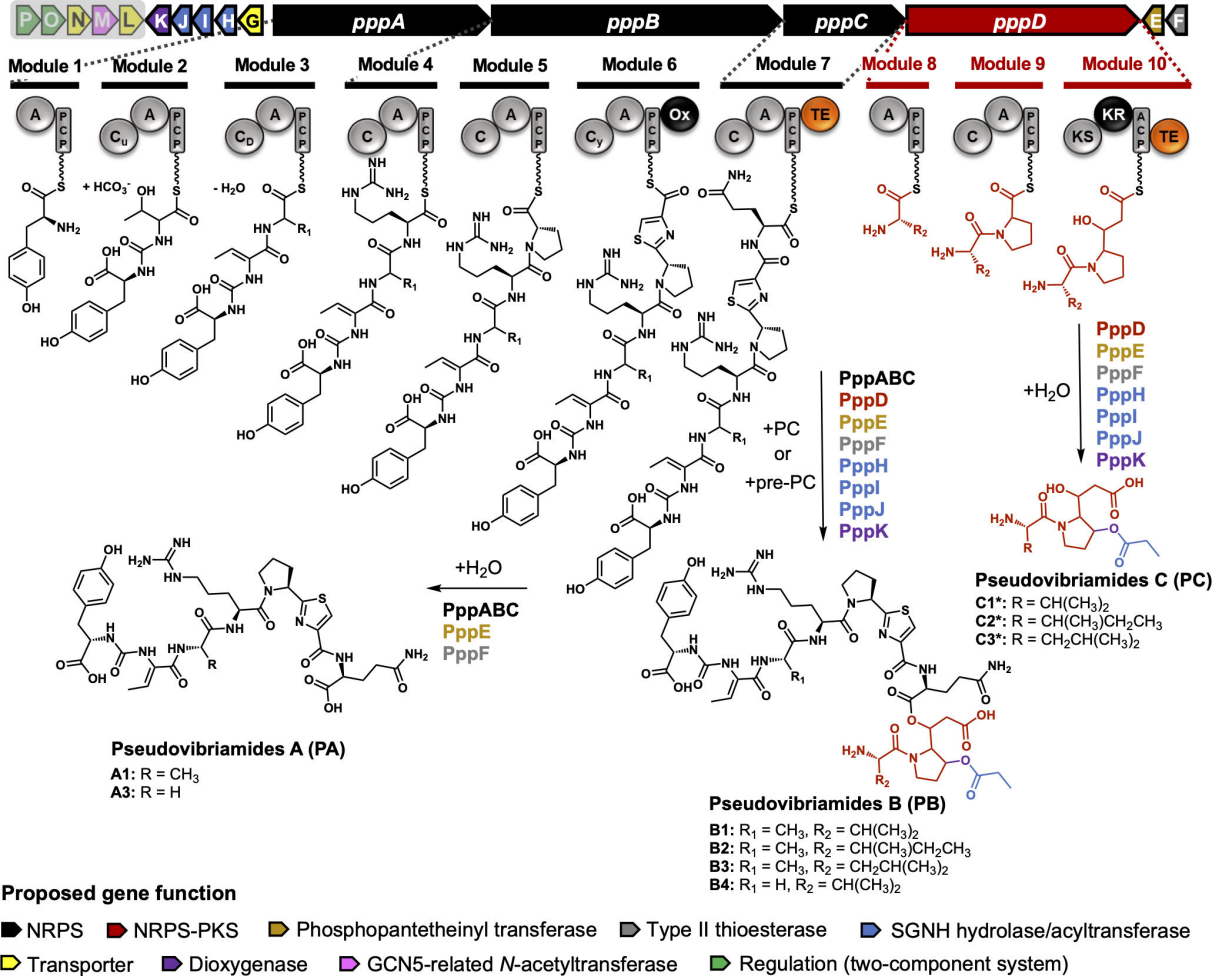
Organization of the *ppp* gene cluster from *P. brasiliensis* Ab134, and proposed biosynthesis of pseudovibriamides A, B, and C. Biosynthetic proposal based on gene knockout results reported in this study. Structural components are color-coded according to genes that encode them. Enzymes proposed to be involved in the biosynthesis of each pseudovibriamide are listed next to the arrows. NRPS, nonribosomal peptide synthetase. PKS, polyketide synthase. See Table S1 for further details on the ppp proteins. Domain key: A, adenylation; ACP/PCP, acyl/peptidyl carrier protein; C, condensation; C_u_, ureido-linkage formation condensation domain; C_D_, dehydration condensation domain; C_y_, condensation and heterocyclization; KR, ketoreductase; KS, ketosynthase; Ox, oxidase; TE, thioesterase. Figure adapted from ref (10). The structures of PA and PB have been previously reported (10). PC structures (*) are proposed based on mass spectrometry analyses performed in this study. Genes proposed to be outside the *ppp* gene cluster are shaded.

The *ppp* BGC consists of genes *pppABC* encoding nonribosomal peptide synthetases (NRPS), *pppD* encoding a hybrid NRPS-polyketide synthase (NRPS-PKS), and multiple flanking genes *pppE to pppP* (Fig. 1; Table S1). NRPS *pppA::neo* mutants helped us identify the products of the *ppp* BGC, which we termed pseudovibriamides A, and B (PA and PB) (10). *P. brasiliensis* Ab134 also accumulates a third product or intermediate, pseudovibriamide C (PC), detected here by mass spectrometry analysis. Impaired swimming and swarming motility were reported for *pppA::neo* mutants, but not for *pppD::neo* mutants, indicating that only PA is required for wild type level motility (10).

However, an understanding of how pseudovibriamides affect flagellar motility is lacking. Flagellar motility is a complex behavior that involves many factors, including chemotaxis and quorum sensing, flagella which are driven by the proton motive force, and, in the case of swarming, metabolites like surfactants (9). Most nonribosomal peptides that are known to mediate swarming are lipopeptide surfactants (17). Surfactants facilitate swarming by lowering the surface tension and easing cell spreading. Pseudovibriamides have no surfactant activity, as expected from their chemical structures (10). Alternatively, nonribosomal peptides, including those with a surfactant nature, can function as signaling molecules, exerting an effect on transcription and regulating various phenotypes (18–21).

Herein we report and discuss the results of a study aiming to gain insight into pseudovibriamide biosynthesis using reverse genetics, while obtaining mutants with different compositions of pseudovibriamides to ultimately test the hypothesis that pseudovibriamides act as signaling molecules, regulating genes affecting flagellar motility.

## RESULTS

### Biosynthetic insights from mutagenesis studies and access to strains with different compositions of pseudovibriamides

Due to the lack of pure pseudovibriamides, having mutants that produce only one of the pseudovibriamide congeners would facilitate probing their individual roles. According to the scheme presented in Fig. 1, PB is the full-length product. PA and PC could conceivably either represent hydrolysis products of PB catalyzed by an accessory hydrolase or be directly released from the assembly lines using water as the nucleophile in reactions catalyzed by thioesterase (TE) domains present in modules 7 and 10, respectively. To probe these two hypotheses and to assign functions to accessory genes, we employed reverse genetics. The only genes that had been previously inactivated were *pppA* and *pppD* (10). However, mutants had been generated by replacement with a selectable marker. To avoid polar effects, we generated markerless, in-frame deletion mutants of *pppA* and *pppD*, in addition to each of nine predicted accessory genes, i.e., *pppE* to *pppM* (Fig. 1). Prediction of the borders of the BGC was based on MultiGeneBlast results from our previous investigation (10), that showed *pppA-pppD* and *pppG-pppK* to be conserved in *Pseudovibrio* and *Pseudomonas*, *pppEF* to be conserved in *Pseudovibrio* and *pppL-pppP* to be present in some *Pseudovibrio* strains.

All *P. brasiliensis* mutants were generated using homologous recombination and confirmed by PCR (Fig. S1 and S2). Different congeners of PA, PB, and PC are produced by *P. brasiliensis* (Fig. 1). Here we will refer only to the major congeners PA1, PB1/2/3 (Fig. S3) and PC1/2/3 (Fig. S4 and S5) as identified by Matrix-Assisted Laser Desorption/Ionization Time of Flight (MALDI-ToF) mass spectrometry (MS) and liquid chromatography MS (LC-MS), respectively, since minor congeners are not always detected.

**Fig 2:**
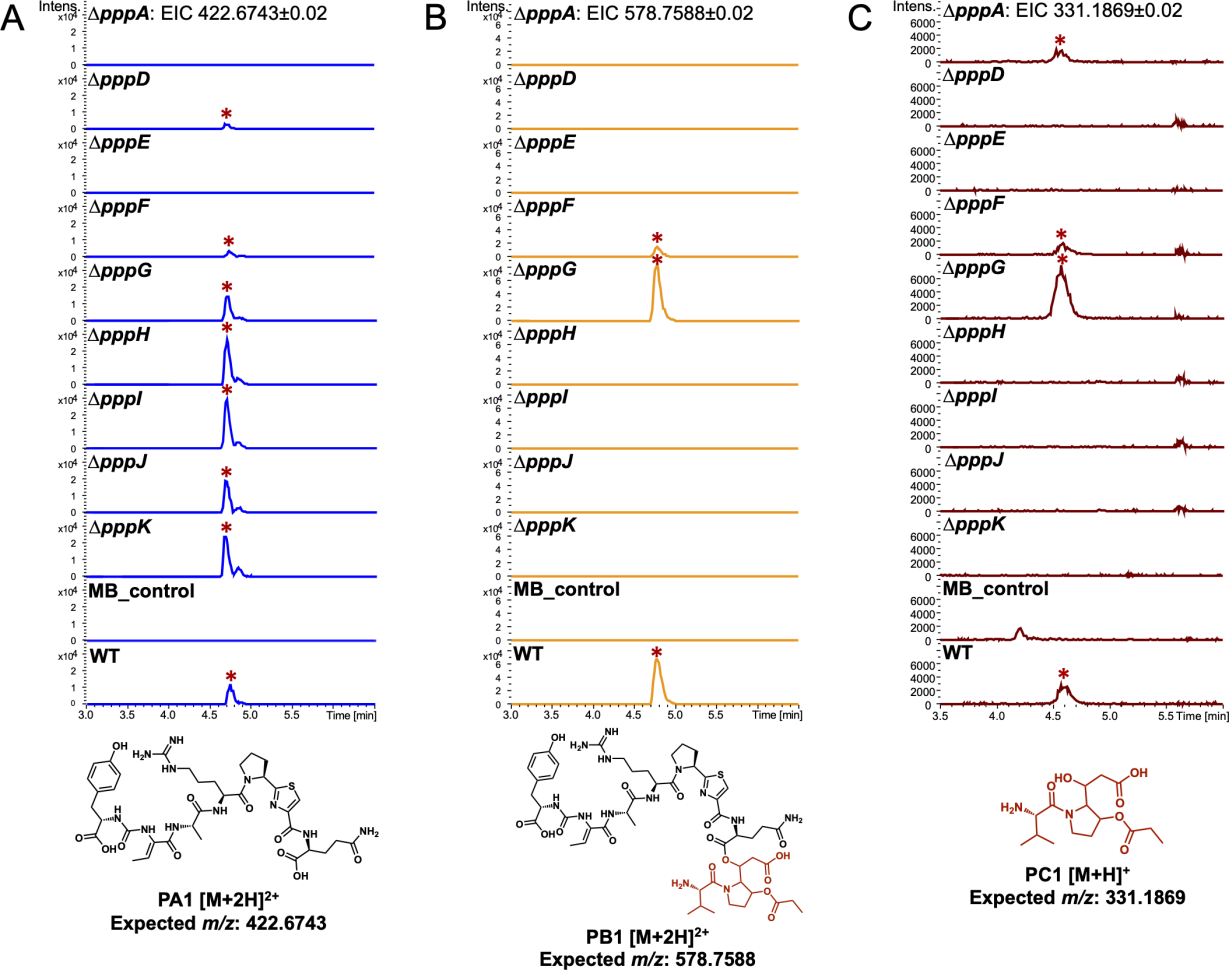
Comparison of the production of PA, PB, and PC representatives between the wild type (WT) and mutants. Extracted Ion Chromatograms (EIC) from LC-MS analyses of WT and mutants. (**A**) PA1. (**B**) PB1. (**C**) PC1. Pseudovibriamides-related EIC peaks are marked with red asterisks. Marine broth (MB) extracts were used as negative control. The same mass filter (the expected *m/z* ± 0.02) was applied to all extracts. The same intensity scale was applied in between strains for each pseudovibriamide. All analyses were performed in at least triplicates.

Deletion of flanking genes *pppL* and *pppM* showed no effect on the pseudovibriamides produced by *P. brasiliensis* (Fig. S6 to S8). Genes *pppLMNOP* appear to be part of the same operon (Fig. 1), and they are not conserved in all *Pseudovibrio* strains (10) which agrees with a non-requirement for pseudovibriamide biosynthesis. They were thus assigned as not part of the *ppp* BGC and we did not generate deletion mutants of *pppN*, *pppO*, and *pppP*. In contrast, we observed changes in pseudovibriamide production for most other *P. brasiliensis* mutants (Fig. 2) as described below.

The *∆pppA* mutant did not produce PA or PB, matching the result of our previous study using *pppA::neo* mutants which had been genetically complemented (Fig. 2A and B; Fig. S9 to S11) (10). However, we observed the production of a metabolite we tentatively identified here as PC by MS analyses, consistent with PppD being still intact in the *∆pppA* mutant (Fig. 2C; Fig. S4 and S5). In contrast, the *∆pppD* mutant produced only PA in agreement with previous results using *pppD::neo* (Fig. 2; Fig. S9 to S11) (10). Genetic complementation using pYDcompD (Table S2) successfully recovered the production of PB and PC (Fig. S12).

The *∆pppE* mutant produced no pseudovibriamides consistent with PppE’s crucial role as a 4’-phosphopantetheinyl transferase to activate NRPS and NRPS-PKS enzymes (Fig. 2; Fig. S4, S5, S9 to S11) (22). Genetic complementation using pYDcompE successfully recovered the production of pseudovibriamides (Fig. S13). The *∆pppF* mutant showed a reduction in overall pseudovibriamide abundance, whereas genetic complementation using pYDcompF improved pseudovibriamide production (Fig. S14), consistent with a role for PppF as a type II, proofreading thioesterase that regenerates mis-acylated ACPs (23, 24).

Mutants *∆pppH, ∆pppI,* and *∆pppJ* produced only PA (Fig. 2). Blast analyses revealed sequence similarity to unknown proteins, except for PppH which showed sequence similarity to SGNH hydrolases (Table S1). Additionally, protein structure prediction using Phyre2 (25) suggested PppH, PppI, and PppJ belong to hydrolase or acyltransferase families of proteins whereas analysis using CLEAN (26) predicted all three to be transferases. To test for polar effects, we performed genetic complementation using plasmids pDS00H, pDS00I, and pDS00J, respectively. The production of all pseudovibriamides was recovered in each of the *P. brasiliensis* mutants, ruling out polar effects (Fig. S15 to S19). We hypothesize that PppHIJ is involved in propionylation of the hydroxyproline residue and/or function as a *trans*-acyltransferase to load the PKS module of PppD. If PppHIJ is involved in propionylation, we expected to observe a pseudovibriamide analog lacking this modification, however, none was detected. The data suggests that PppHIJ may act together as a complex and they are each crucial for PB and PC biosynthesis. Another hypothesis we investigated was that PppM, predicted to be a GCN5-related *N*-acetyltransferase, could catalyze propionyl transfer or function as the acyltransferase that is missing in module 10. However, as stated above, Δ*pppM* mutants had the same metabolite profile as the WT (Fig. S7 and S8), indicating that *pppM* is not required for pseudovibriamide biosynthesis.

Thus, no PB hydrolase was identified. Instead, it is conceivable that the TE in module 7 can accept either water as the nucleophile leading to PA or products of the PppD enzyme (PC or pre-PC) leading to PB.

### PppK is a hydroxylase

**Fig 3:**
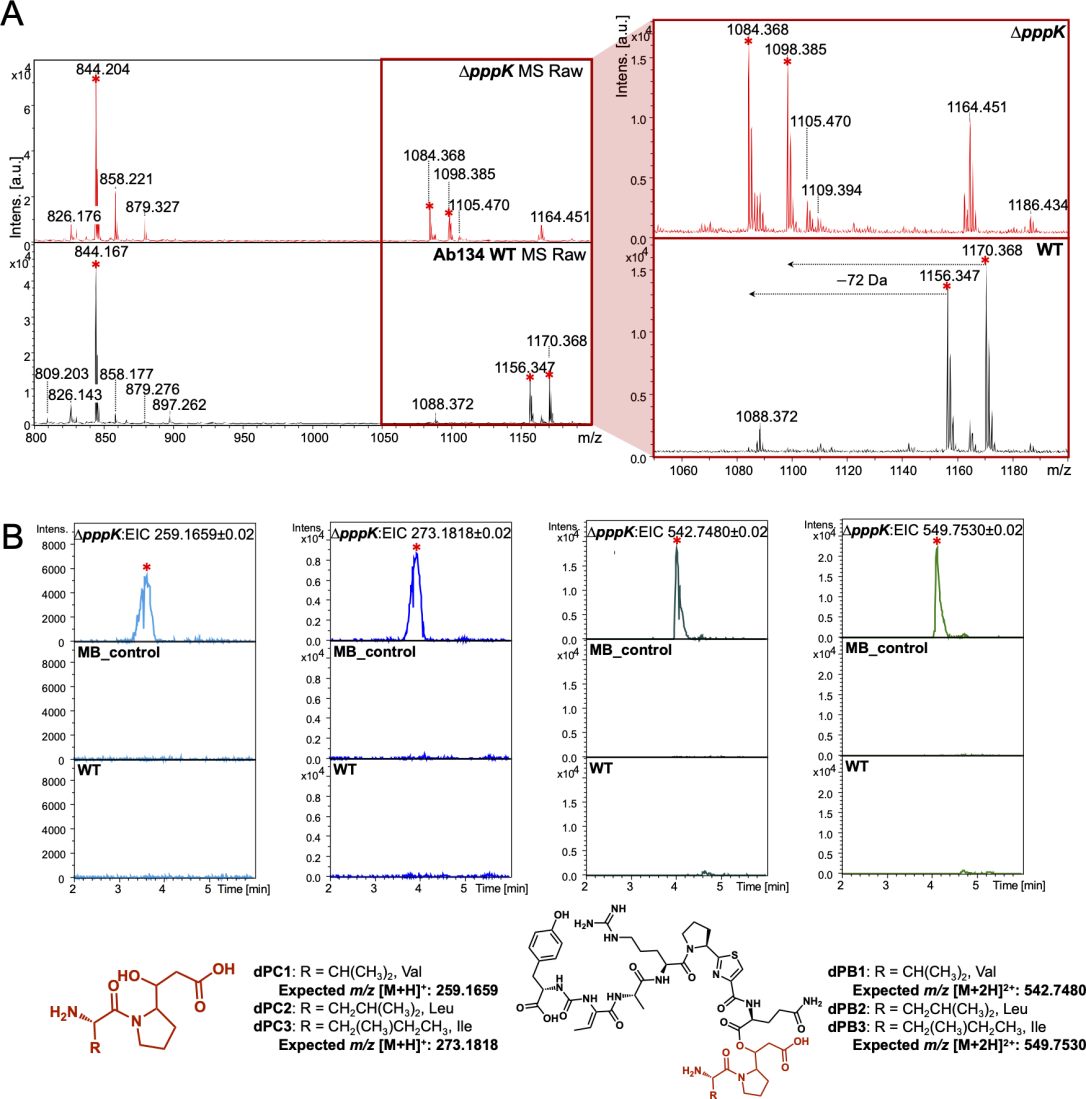
Comparison of PB and PC production between the WT and the *∆pppK* mutant. (**A**) MALDI-ToF MS analyses. Molecular features for pseudovibriamides are indicated with red asterisks. The peak at *m/z* 844.2 represents PA1 ([M + H]^+^); *m/z* 1156.3, PB1 (Val, [M + H]^+^); *m/z* 1170.4, PB2 or PB3 (Ile or Leu, [M + H]^+^); *m/z* 1084.4, depropionylated PB1 (dPB1 Val, [M + H]^+^); and *m/z* 1098.4, depropionylated PB2 or PB3 (dPB2, Leu, [M + H]^+^ or dPB3, Ile, [M + H]^+^). The range from *m/z* 1050 to 1200 was zoomed in (red box) to show the *m/z* change of PBs. (**B**) LC-MS analyses. dPC, depropionylated PC; dPB, depropionylated PB. Extracted Ion Chromatogram (EIC) from left to right: dPCs (Val, [M + H]^+^, *m/z* 259.1659; and Leu or Ile, [M + H]^+^, *m/z* 273.1818), dPBs (Val, [M + 2H]^2+^, *m/z* 542.748; and Leu or Ile, [M + 2H]^2+^, *m/z* 549.753). The same mass filter (the expected *m/z* ± 0.02) was applied to all samples. The predicted structure is listed below each chromatogram. All analyses were performed in at least triplicates.

PppK shows sequence similarity to Fe(II)/α-ketoglutarate dependent dioxygenases (27) and we predicted it would be responsible for the hydroxyl group in the proline residue of PB and PC. Accordingly, the *∆pppK* mutant produced PA as in the WT (Fig. 2) but PB and PC analogs showed a 72 Da mass loss, indicating that they lacked the hydroxyl group on the proline and consequently also lacked propionylation (Fig. 3; Fig. S20 and S21). Fragmentation patterns from MS^2^ spectra further verified the assignment (Fig. S22 and S23). Genetic complementation using plasmid pVL00K recovered the production of PB and PC (Fig. S24). The timing of hydroxylation remains unknown. Three scenarios are conceivable. PppK could be either a proline hydroxylase acting on free proline, or it could act in-line while the proline-containing substrate is attached to PppD, or after pre-pseudovibriamides are released from PppD, either on pre-PC or pre-PB. The amino acid code of the second adenylation domain within PppD is consistent with the previously reported selectivity-conferring code of A domains that activate proline (Fig. S25) (28), supporting the latter two scenarios.

### The amount of pseudovibriamides exported is not significantly affected in *∆pppG* and *∆pppL* mutants

According to previous imaging mass spectrometry studies, PA and PB are excreted (10). Both *pppG* and *pppL* genes are predicted to encode transporters that could be responsible for pseudovibriamide export (Table S1). Both *∆pppG* and *∆pppL* mutants produce all pseudovibriamides (Fig. 2; Fig. S4 to S6, S9 to S11). To check for export, we extracted pseudovibriamides from cell pellets and supernatant separately (Fig. S26). The export ratio—defined as the amount of pseudovibriamides in the supernatant by the amount in the cell pellet—of PB1, PB2, and PB3 was slightly higher for the WT and genetically complemented *∆pppG* pYDcompG compared to *∆pppG* cultures, but not statistically significant (Fig. S27A). Likewise, no statistically significant difference was observed between the WT and the *∆pppL* mutants (Fig. S27B, S28 to S40). Thus, the protein involved in pseudovibriamide export remains to be identified. It is possible that *pppG* and *pppL* may compensate for each other, which could explain the lack of significant differences in the single mutants, a topic that may be investigated in the future.

### Effect of gene deletion on flagellar motility

**Fig 4:**
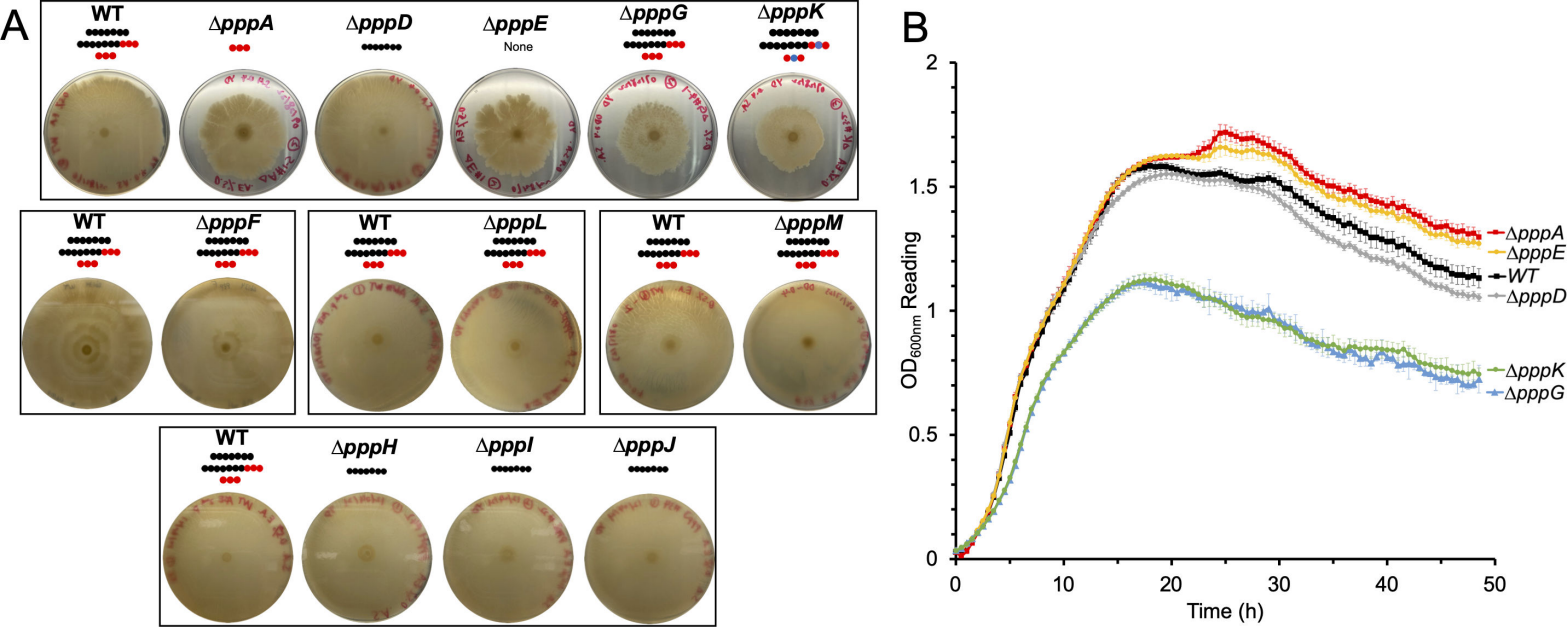
Effect of *ppp* gene inactivation on flagellar motility. (**A**) Swarming assays performed on marine broth with 0.5% Eiken agar. Pictures shown were taken 72  h after inoculation. The assay was performed multiple times, each time in at least triplicates with the WT as the control, and similar results were obtained each time (see Fig. S41 to S49). Representative results are shown. Plates are grouped and boxed based on assays that were run together the same day. Pseudovibriamides are represented by beads: PA, seven black beads; PB, seven black beads and three red beads; PC, three red beads; depropionylated PB, seven black beads, two red beads, and one blue bead; depropionylated PC, two red beads and one blue bead. (**B**) Growth of strains from top box in panel A as measured by OD_600_. *N* = 6. Error bars indicate standard deviation. Note that the apparent reduced swarming of Δ*pppG* and Δ*pppK* mutants is in fact due to reduced growth.

We performed swarming assays for the 11 in-frame deletion mutants generated here. Both *∆pppA* and *∆pppD* mutants showed consistent swarming phenotypes as reported previously (Fig. 4; Fig. S41 and S42) (10). Moreover, WT level swarming motility was observed for *∆pppH, ∆pppI,* and ∆*pppJ* mutants, which have the same metabolite profile as the *∆pppD* mutant, that is, production of PA only (Fig. S43). In contrast, the *∆pppE* mutant that produces no pseudovibriamides showed decreased swarming motility like the *∆pppA* mutant that produces only PC (Fig. 4; Fig. S44). The *pppA::neo* mutant had been previously genetically complemented showing restoration of swarming proficiency (10). Likewise, the swarming motility of the *∆pppE* mutant was successfully recovered by genetic complementation (Fig. S45).

The combined results of *∆pppD, ∆pppH, ∆pppI,* and ∆*pppJ* mutants suggest that only PA is required for WT level swarming motility. However, both *∆pppK* and *∆pppG* mutants showed impaired swarming, even though PA was produced in these mutants (Fig. 4A). Pseudovibriamide production was restored by genetic complementation using pVL00K and pYDcompG, respectively (Fig. S24); however, swarming was not, suggesting factors other than pseudovibriamides were involved in the phenotype of Δ*pppK* and Δ*pppG* mutants (Fig. S46). It turns out both *∆pppG* and *∆pppK* mutants showed decreased growth, suggesting the cause of apparently reduced swarming motility is in fact related to reduced growth (Fig. 4B). Whole genome sequencing of both *∆pppG* and *∆pppK* mutants showed several potential mutations (Table S3 and S4). It remains to be shown which mutations are responsible for the reduced growth phenotype.

Other mutants (∆*pppF*, ∆*pppH*, ∆*pppI*, ∆*pppJ*, ∆*pppL*, and ∆*pppM*) showed no observable defect in swarming motility (Fig. 4A; Fig. S43, S47 to S49).

### Transmission electron microscopy identifies no apparent changes in flagella

We used transmission electron microscopy (TEM) to visualize cells of strains with different compositions of pseudovibriamides, that is, *∆pppA*, *∆pppD*, *∆pppE* mutants, and the WT. No apparent differences in flagella were observed between the strains, although there seemed to be more cell aggregation for *∆pppA* and *∆pppE* mutants which is consistent with decreased swarming motility (Fig. S50).

### PA and PB modulate gene transcription whereas PC does not

**Fig 5:**
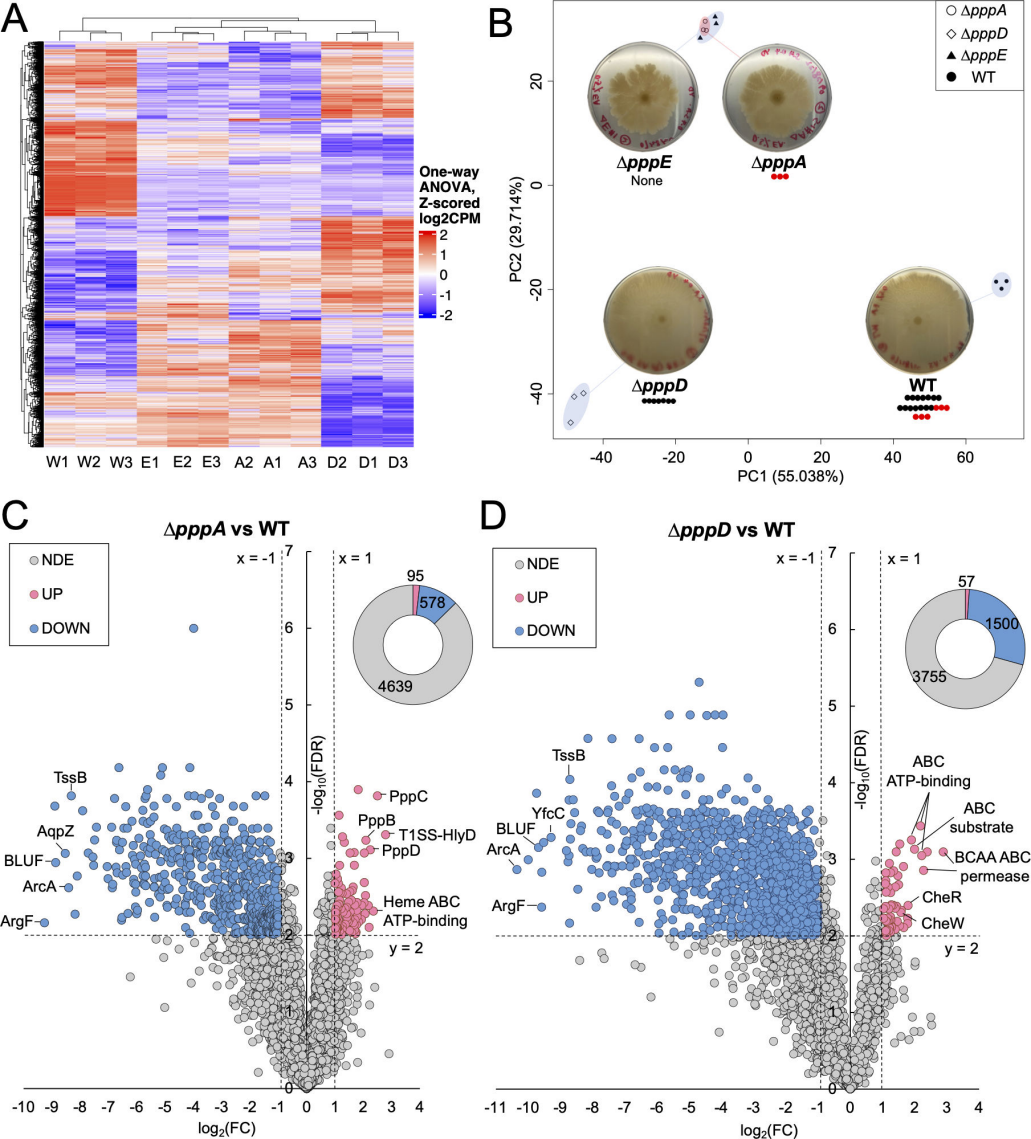
Overview of RNA-seq results. (**A**) Heatmap of gene expression based on Z-scored counts per million (CPM) of the WT (W1-3), *∆pppA* (A1-3), *∆pppD* (D1-3), and *∆pppE* mutant (E1-3). Each column represents one of three replicates. Each row represents one of 5,312 genes. One-way analysis of variance (ANOVA) was used to compare whether four samples’ means are significantly different or not. Values in each row were scaled to CPM mean of the row by using Z-score normalization. (**B**) Principal component analysis (PCA) of gene expression based on normalized CPM of triplicate samples. Corresponding swarming images at 72 h (duplicated from Fig. 4A for convenience) are shown and pseudovibriamide composition is indicated for each strain. Seven black beads represent PA; seven black beads plus three red beads represent PB; and three red beads represent PC. (**C** and **D**) Volcano plots of differentially expressed (DE) genes identified between the WT and *∆pppA* and *∆pppD* mutants, respectively, using transcripts per million (TPM). FDR, false discovery rate or *q*-value; FC, fold-change; NDE, non-differential expressed; UP, upregulated; DOWN, downregulated; Blue dots or blue donut portion, downregulated genes; Pink dots or pink donut portion, upregulated genes; and gray dots or gray donut portion, non-differentially expressed genes. The same threshold (dotted lines) was applied to all differential expression analyses, that is, FC ≤ −2 (x = −1) or FC ≥2 (x = 1), and FDR ≤ 0.01 (y = 2). The total number of UP, DOWN, and NDE genes are listed in the donut chart. Labeled genes are TssB, type VI secretion system contractile sheath small subunit; YfcC, Arginine/ornithine antiporter; AqpZ, aquaporin Z; ArcA, arginine deiminase; ArgF, ornithine carbamoyltransferase; BLUF, blue light using flavin domain; T1SS-HlyD, HlyD family type I secretion periplasmic adaptor subunit; BCAA ABC permease, branched-chain amino acid ATP-binding cassette transporter permease; ABC ATP-binding, ATP-binding cassette transporter ATP-binding protein; ABC substrate, ATP-binding cassette transporter substrate binding protein; Heme ABC ATP-binding, heme ATP-binding cassette transporter ATP-binding protein; CheW, chemotaxis protein; and CheR, protein-glutamate O-methyltransferase. Some outstanding dots left unlabeled are hypothetical proteins.

To test the hypothesis that pseudovibriamides affect motility by modulating gene transcription, RNA-sequencing (RNA-seq) data sets were obtained for the WT and *∆pppA*, *∆pppD*, and *∆pppE* mutants having different compositions of pseudovibriamides (Fig. 5). Triplicate samples of each strain were similar in terms of gene expression pattern, which is apparent in the principal component analysis (PCA) plot where triplicate samples from each strain clustered together, supporting the quality of the data (Fig. 5B). The highly similar transcriptomic profiles of *∆pppA* and ∆*pppE* mutants indicates that PC has none or only minimal effect on gene transcription (Fig. 5B). In contrast, the large differences in principal components 1 and 2 observed between the WT and either the *∆pppD* mutant or the *∆pppA/∆pppE* mutants indicates that PA and PB have major effects on gene transcription (Fig. 5B).

### Pairwise differential expression analyses methods and validation

To ensure the identified differentially expressed (DE) genes would have physiological relevance, we started by testing distinct analysis methods and performing follow-up experiments for validation. We first used DESeq2, which is based on negative binomial distribution (Fig. S51). An upregulation of *ppp* core biosynthetic genes was observed in both *∆pppA* and *∆pppD* mutants in which *pppBCD* and *pppABC* appeared upregulated compared to the WT, respectively (Fig. S51). According to the RNA-seq data, a promoter is located upstream of the *pppA* gene (P*_pppA_*) (Fig. S52). Upregulation of *ppp* genes in the mutants is suggestive of negative autoregulation by pseudovibriamides as reported for other products (29). To test this hypothesis, we cloned P*_pppA_* directly upstream of *GFP* in the promoterless pSEVA227M-based vector. In the case of negative autoregulation, we expected to observe increased GFP production in the mutants. However, this was not the case (Fig. S53). Instead, the promoter probe studies suggested that DESeq2 resulted in the identification of false positives.

We next tested transcripts per million (TPM) for data normalization and DE calls (Fig. 5C and D). Genes *pppBCD* were still upregulated but only in the *∆pppA* mutant, which is likely an artifact of bringing P*_pppA_* closer to the other genes in the operon by deleting ~8,000 bp of the *pppA* gene. Thus, the results indicate that TPM is more accurate for comparing gene expression levels across our samples. Therefore, TPM values were chosen for further analyses. The total number of DE genes was reduced from 1,298 to 673 in the *∆pppA* mutant, and from 1,616 to 1,557 in the *∆pppD* mutant when using TPM (Fig. 5C and D; Fig. S51). There was only one gene, *pppC*, upregulated, and no downregulated gene in the *∆pppA* mutant when compared to *∆pppE* mutant using FDR ≤ 0.01 as the cutoff. Because the PCA plots overlap, we considered *∆pppA* and *∆pppE* mutants to be indistinguishable, and the FDR cutoff of ≤0.01 was appropriate for further analyses.

### Global effects of PA and PB

**Fig 6:**
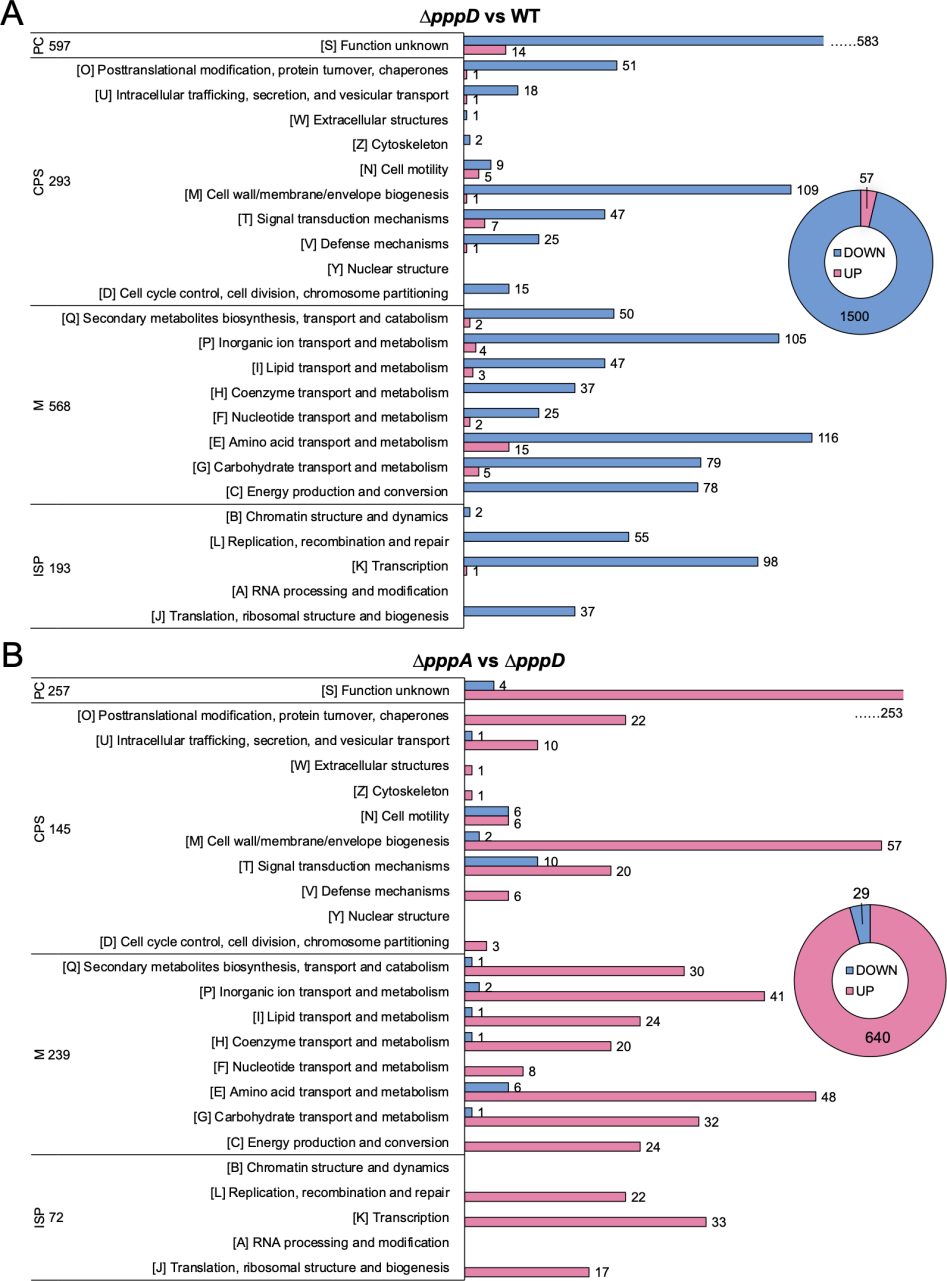
COG classification of DE genes potentially affected by PA and PB. (**A**) DE genes of *∆pppD* mutant compared to the WT. (**B**) DE genes of *∆pppA* mutant compared to the *∆pppD mutant*. Blue, downregulated; Pink, upregulated. PC, poorly characterized; CPS, cellular processes and signaling; M, metabolism; and ISP, information storage and processing. The total number of genes in each category is listed.

The large number of DE genes identified (Fig. 5C and D) suggests that PA and PB have a global effect on transcription that goes beyond motility. To obtain a broader view of the role of PB, DE genes of *∆pppD* mutant (missing PB but having PA) when compared to the WT were classified based on clusters of orthologous genes (COG) (Fig. 6A; File S1). Except for the poorly characterized (PC) group, the metabolism (M) group is the largest, followed by the cell processes and signaling (CPS) group (Fig. 6A). Regarding specific categories within these groups, cell wall biogenesis [M], inorganic ion transport and metabolism [P], amino acid transport and metabolism [E], and transcription [K] dominate (Fig. 6A; File S1). Strikingly, downregulated genes outnumber upregulated genes by roughly 30 to 1, indicating that PB may have a primary effect on gene activation.

Regarding genes potentially affected by PA, the *∆pppA* mutant (missing PA) was compared to the *∆pppD* mutant (produces only PA) (Fig. 6B; File S1). Contrary to what was observed in the previous comparison, upregulated genes outnumber downregulated genes by roughly 20 to 1 in the *∆pppA* mutant, indicating that PA may have an inhibitory effect on gene transcription. Metabolism is still the largest group followed by cell processes and signaling. The categories most affected are also the same (Fig. 6B). Thus, the results suggest that PB and PA may have opposite roles in modulating gene transcription. Indeed, 446 DE genes overlap between the two pairwise analyses and are inversely regulated (File S1).

### Identifying DE genes potentially involved with differential flagellar motility

We considered two assumptions for narrowing down potential candidate genes related to flagellar motility. On one hand, the *∆pppD* mutant may possess the same set of genes unaffected compared to the WT, which are DE in *∆pppA* and *∆pppE* mutants, resulting in reduced motility. On the other hand, the *∆pppD* mutant might harness different pathways than the WT for promoting motility, resulting in the same observable phenotype.

**Fig 7:**
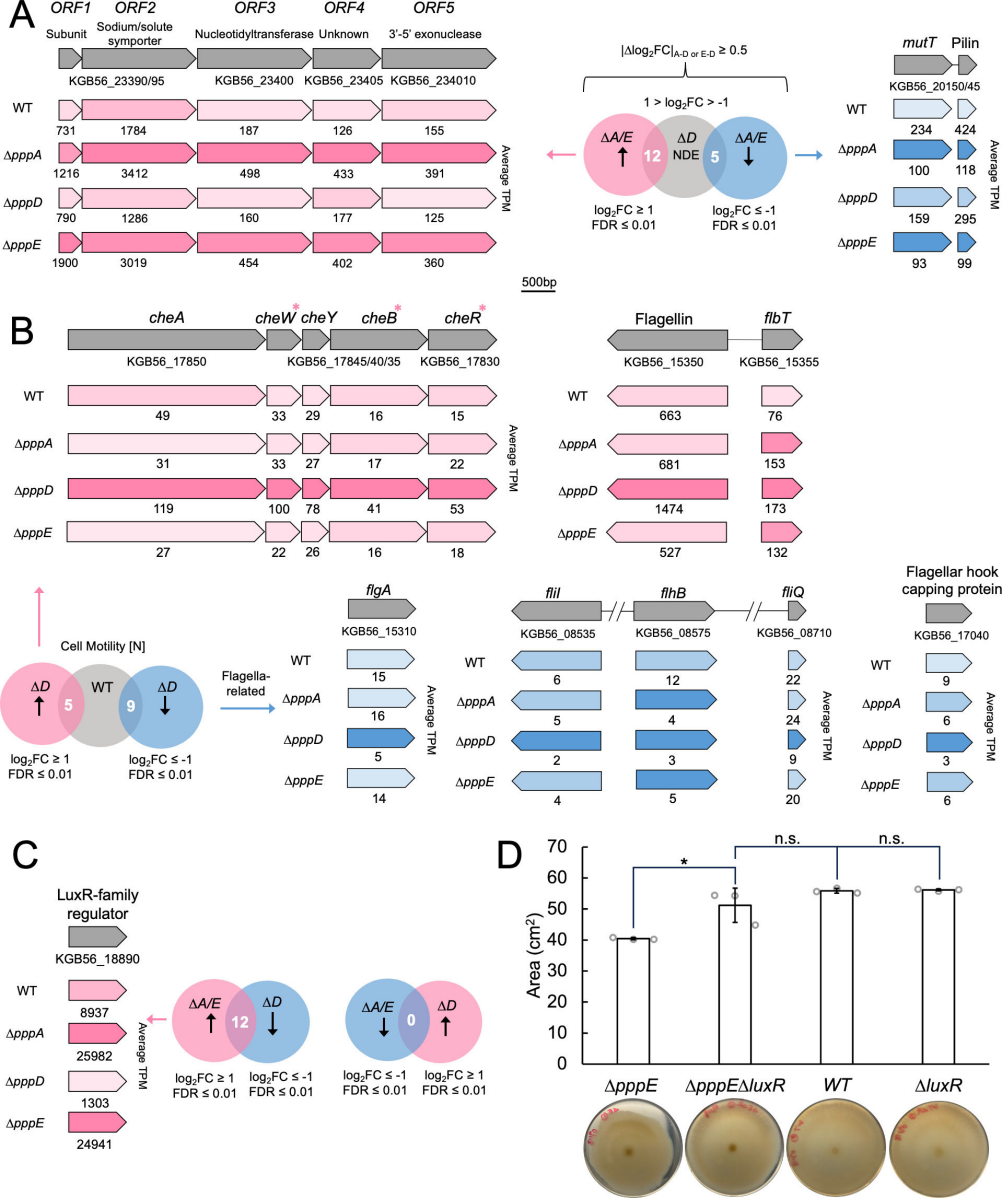
DE genes potentially involved in differential swarming motility. (**A**) Assumption 1: the ∆*pppD* mutant may possess the same set of genes unaffected compared to the WT, which are DE in ∆*pppA*/∆p*ppE* mutants compared to the WT. Filters used for selecting such genes. |∆log_2_FC|, the absolute difference of log_2_FC between *∆pppA,* or *∆pppE* and *∆pppD* mutants was set to be larger than or equal to 0.5 to exclude genes with minor variation in differential expression. See Fig. S54A for COG categories. Examples of DE operons are shown, that is, a five-gene operon that is upregulated in theΔ*pppA/E* mutants (subunit, the small subunit of the sodium/solute symporter), and a two-gene operon that is downregulated in theΔ*pppA/E* mutants including MutT CDS (KGB56_20150) and Flp family type IVb pilin CDS (KGB56_20145). Pink, upregulated; blue, downregulated. The darker the pink, the higher the relative expression level. The darker the blue, the lower the relative expression level. TPM values are indicated below genes. (**B**) Assumption 2: the ∆*pppD* harnesses different pathways than the WT for promoting swarming motility, resulting in the same observable phenotype. Selected motility genes that are up or downregulated in theΔ*pppD* mutant are shown. Upregulated chemotaxis genes (FDR ≤ 0.01) are denoted with pink asterisks. *cheA*, chemotaxis histidine protein kinase; *cheW*, linker protein; *cheY*, chemotaxis response regulator; *cheB*, chemotaxis response regulator protein-glutamate methylesterase; *cheR*, chemotaxis glutamate *O*-methyltransferase; *flbT*, flagellar biosynthesis repressor; *flgA*, flagellar basal body P-ring formation protein; *fliI*, flagellar biosynthesis type III secretory pathway ATPase; *flhB* and *fliQ,* flagellar biosynthesis protein. (**C**) Filters used for selecting DE genes in ∆*pppA*/∆p*ppE* mutants that are reversely regulated in the ∆*pppD* mutant compared to the WT. See Fig. S55 for COG categories. The expression levels (in TPM) of a LuxR family transcriptional regulator CDS (KGB56_18890) are indicated for mutants and the WT. The darker the pink, the higher the expression level. (**D**) Comparison of swarming areas between the WT, the *∆luxR* mutant, the *∆pppE* mutant, and the ∆*pppE ∆luxR* mutant. Individual data points and standard deviation are shown (Table S7). Two-tail *P*-values from *t*-test were used to determine statistical significance; *, *P*-value ≤ 0.05; n.s., not significant. Swarming pictures shown were taken 96  h after inoculation (Fig. S58).

Assuming the first scenario, genes were compiled that were DE in both *∆pppA* and *∆pppE* mutants but also non-differentially expressed (NDE) in the *∆pppD* mutant, each compared to the WT (Fig. 7A). As a result, 12 upregulated genes and five downregulated genes in *∆pppA/E* mutants were identified (Fig. 7A; Fig. S54A; Table S5). According to COG, most upregulated genes, except those encoding hypothetical proteins, are ATP-binding cassette (ABC) transporters belonging to the metabolism category. This pattern generally matches observations from a study in *Pseudomonas aeruginosa* where the authors found that genes related to the transport of small molecules were upregulated in non-swarming cells (30).

There were no genes in the cell motility [N] category. We next investigated signal transduction [T], which could include genes regulating the swarming motility phenotype directly or indirectly. Of the two genes upregulated in the T category (Table S5, Supplementary Results section), one is predicted to encode a nucleotidyltransferase and is located within a five-gene operon (Fig. 7A). The other four genes were not hits due to the stringent cut off we set, but they still show considerable log_2_FC of 0.73–1.78. ORF2 encodes a sodium/solute symporter, and ORF1 encodes its small subunit. These genes are highly expressed in *∆pppA/E* mutants, for example, the TPM value of 3412 for ORF2 ranks as the 25th most highly transcribed gene. An overexpression of this symporter (31) will not only increase the uptake of nutrients but also of sodium. Sodium influx is used to power flagellar rotation (32, 33). It is possible that an imbalance in the sodium gradient in *∆pppA/E* mutants slows down the flagellar motor, leading to reduced but not abolished motility. The remaining one gene upregulated in the signal transduction [T] category is described under Supplementary Results section.

There were only five downregulated genes in *∆pppA/E* mutants but NDE in the *∆pppD* mutant (Fig. 7A; Fig. S54A, Table S5). One of them is predicted to encode a Nudix hyrdolase (34, 35) (Fig. S54B). Inactivation of Nudix hydrolase genes *mutT* from *Escherichia coli* and *PA4400* from *P. aeruginosa* results in a higher mutation rate, and *PA4400* can complement an *E. coli mutT* deficient strain (36, 37). A recent study showed that *P. aeruginosa* ∆*PA4400* possessed severely impaired swarming motility (38). Thus, the downregulation of this gene could help explain the reduced swarming motility of *∆pppA/E* mutants. In addition, the gene downstream of the *mutT* homolog is predicted to encode a type IV pilin subunit (39). A previous study showed that a *P. aeruginosa* type IV pilin mutant was unable to swarm (40). Thus, downregulation of the type IV pilin gene in *∆pppA/E* mutants may contribute to the attenuated swarming motility as well.

To probe the second scenario that the *∆pppD* mutant might harness different pathways than the WT for promoting motility, the direct pairwise differential expression analysis between *∆pppD* mutant and the WT was analyzed (Fig. 6A). In the most obvious category, cell motility [N], there were five genes upregulated in the *∆pppD* mutant (encoding one flagellin protein, one flagellar biosynthesis repressor FlbT, and three chemotaxis proteins CheW, CheB and CheR that are part of a five-gene operon), and nine genes downregulated (encoding five flagella-related proteins; three L,D-transpeptidases, and one type IV secretion system protein) (Fig. 6A and 7B; File S1). The downregulation of genes encoding components of flagella and the upregulation of a flagella biosynthesis repressor gene suggests the *∆pppD* mutant should have reduced motility unless there was a compensatory mechanism, which seems to be the case with the upregulation of chemotaxis genes. Downregulation of chemotaxis genes was observed in a swarming-deficient mutant of *Vibrio parahaemolyticus* (41) conversely, upregulation might promote motility in the *∆pppD* mutant as a compensatory mechanism.

To identify genes that may be implicated in the opposing effects of PA and PB, we searched for genes that were DE in opposite directions in the *∆pppD* and *∆pppA/E* mutants when compared to the WT, respectively, and identified 12 genes (Fig. 7C; Fig. S55; Table S6). One gene caught our attention because it is predicted to encode an orphan LuxR-type transcriptional regulator (KGB56_18890) (Fig. 7C; File S1). The original *luxR* encodes a well-known cell-density-dependent transcriptional regulator involved in quorum sensing in *Vibrio fischeri* and associated with *luxI* that encodes acyl-homoserinelactone synthase to produce the autoinducer (42). Although we identified no *luxI* homolog in the genome of *P. brasiliensis*, in accordance with previous reports of other sequenced *Pseudovibrio* genomes (43, 44), the LuxR-type transcriptional regulator does contain an *N*-terminal autoinducer-binding domain (pfam03472). Interestingly, a type III PKS BGC located in the same plasmid as *ppp* shows sequence similarity to alkylresorcinol BGCs (e.g., 32% identity to type III PKS SrsA from *Streptomyces*, accession code BGC0000282) (45). An orphan LuxR from *Photorhabdus* bacteria, PauR, has been shown to sense alkylresorcinols (46). Although the identity of *P. brasiliensis* LuxR to PauR is low (20% at the amino acid level), the transcription pattern of the type III PKS (KGB56_24430) is the same as that of the LuxR gene, that is, high in the ∆*pppA/E* mutants, moderate in the WT, and low in the ∆*pppD* mutant, opening an avenue for future studies (Table S6).

Many LuxR variants exist, including orphan QscR and VjbR (47, 48) which show sequence identity to the DE LuxR we identified albeit low (26%). Analogously to what we observed with Δ*pppA/E* mutants, upregulation of QscR from *P. aeruginosa* PAO1 resulted in reduced swarming motility (47). Another gene in the signal transduction [T] category is described under Supplementary Results section.

To test whether upregulation of the *luxR* gene (KGB56_18890) contributes to the reduced motility phenotype of the *∆pppE* mutant, we followed two approaches. The first one was overexpression of the *luxR* gene in the WT and in the *∆pppD* mutant. We first attempted to clone the *luxR* gene using the pAM4891 vector that contains a constitutive promoter and we used for the previous complementation experiments. However, all clones we obtained contained mutations, most of which were stop codon mutations that would likely render the protein non-functional, indicating that this LuxR protein is toxic in *E. coli*. Attempting to clone the *luxR* gene under its own promoter led to similar results. Finally, cloning using a l-rhamnose-inducible promoter was successful. However, WT and *∆pppD* mutant containing pYDrhaR showed decreased growth even in the absence of inducer which prevented accurate comparison of their motility phenotypes (Fig. S56 and S57). We observed a similar impact on growth when using another inducible vector, whereas pAM4891 containing a constitutive promoter and used for complementation studies here did not show a growth defect (49). We next deleted the *luxR* gene in the WT and in *∆pppE* backgrounds. Deletion of the *luxR* gene in the WT delayed the onset of swarming as observed at 24 and 48 h (Fig. S58). In contrast, we observed no difference in swarming for the WT and *∆luxR* mutant at 72 and 96 h. However, the average swarming area of the *∆pppE ∆luxR* mutant was statistically larger than that of the *∆pppE* mutant at these later time points (Fig. 7D; Fig. S58; Table S7). The change in motility was unrelated to growth (Fig. S59). This suggests that expression of *luxR* at the levels observed in the WT is important for WT level motility.

Taken together, compensatory mechanisms seem to be at play, resulting in the same observable flagellar motility phenotype between the WT and the *∆pppD* mutant. Moreover, a *luxR* gene was identified that when upregulated contributes to the reduced swarming motility of the *∆pppE* mutant.

## DISCUSSION

The term “holobiont” has been coined to express the crucial relationship between plants and animals and their associated microbes (4). Microbial metabolites are important in establishing and maintaining microbe-host associations. For instance, motility, mediated by bacterial metabolites, is known to be important for host colonization (9). We previously identified a link between flagellar motility and pseudovibriamides (10). The *ppp* gene cluster that encodes pseudovibriamides (Fig. 1) is found not only in bacteria that interact with marine sponges but also in terrestrial bacteria that interact with plants and animals (10).

The main goal of the present work was to reveal how pseudovibriamides affect flagellar motility. We started by obtaining mutant strains with different compositions of pseudovibriamides while gaining insight into pseudovibriamide biosynthesis. We considered two hypotheses to explain the presence of PA, PB and PC in *P. brasiliensis*. Either PA and PC represent hydrolysis products of PB catalyzed by an accessory hydrolase, or PA is directly released from PppC catalyzed by the thioesterase domain using water as the nucleophile (Fig. 1). We considered PppH as a PB hydrolase because it shows sequence similarity to the hydrolase family of enzymes. If true, we would expect the hydrolase-inactive mutant to produce only PB. However, the Δ*pppH* mutant produced only PA (Fig. 2). None of the accessory genes was identified as encoding a PB hydrolase. Instead, the second hypothesis seems plausible that the TE domain in PppC can accept either water as nucleophile resulting in PA or it can accept PC (or pre-PC) resulting in PB. *In vitro* studies are necessary to further test this hypothesis.

Swarming motility assays suggested that only PA is required for WT level motility. Mutants that produced only PA (*∆pppD, ∆pppH, ∆pppI,* and ∆*pppJ*) displayed motility comparable to the WT (Fig. 4), whereas mutants that produced only PC (*∆pppA*) or no pseudovibriamides (*∆pppE*) showed reduced motility (Fig. 4).

We next performed transcriptomic studies of the WT and of mutant strains that produce either only PA (Δ*pppD*), only PC (Δ*pppA*) or no pseudovibriamides (Δ*pppE*) to test whether pseudovibriamides influence gene transcription. Since several RNA-seq normalization methods are available and errors in normalization can result in false positives (50), we first compared normalization methods and performed follow up experiments for validation using a GFP reporter assay to ensure the identified DE genes would have physiological relevance. From the two methods tested—DESeq2 and TPM, representing normalization by distribution and library size, respectively (50)—TPM seemed to eliminate false positives (Fig. 5C and D; Fig. S51). A recent study to evaluate RNA-seq normalization methods also concluded that TPM performed best in preserving biological signal (51).

From the transcriptomic data, we concluded that PC plays no role in modulating transcription since the transcriptomes of Δ*pppA* and Δ*pppE* mutants were equivalent (Fig. 5B). In contrast, PA and PB play major roles as 13% of the total number of genes are differentially expressed when PA and PB are missing (Δ*pppA* vs WT, Fig. 5C) and 29% of the total number of genes are differentially expressed when PB is missing but PA is present (Δ*pppD* vs WT, Fig. 5D). The results also suggested that PA and PB have opposite effects on a subset of 446 DE genes, with PB having a primary role in gene upregulation and PA in downregulation (Fig. 6; File S1). Although the opposite roles of PA and PB seem counterintuitive, a speculation for future studies is that perhaps PB binds preferentially (with higher affinity) to its unidentified receptor in the WT, but given that PA is a fragment of PB, PA can also bind but with lower affinity, and especially in the absence of PB in the Δ*pppD* mutant. Binding of PA may result in a different conformation of the receptor than when PB binds.

Accordingly, a compensatory mechanism appears to be at play in the Δ*pppD* mutant that results in the same observable flagellar motility phenotype as the WT. This is plausible based on the upregulation of chemotaxis genes while flagella component genes are downregulated (Fig. 7B). In addition, 12 genes are inversely regulated between the *∆pppD* mutant and *∆pppA*/*∆pppE* mutants when compared to the WT (Fig. 7C). For instance, the drastic difference in the transcription level of a LuxR-type regulator appears to contribute to the differential swarming motility phenotypes (Fig. 7D).

In conclusion, motility enables bacteria to reach new habitats and to colonize host tissue. Many questions remain to be answered regarding bacterial motility, including identifying which chemical signals mediate motility and how they do so. Here we showed that pseudovibriamides affect motility by modulating transcription, ultimately revealing new signaling molecules. Importantly, the effects of pseudovibriamides appear to extend beyond motility to affect yet-to-be identified phenotypes. Future studies should focus on more detailed biosynthetic investigations such as the timing of hydroxylation by PppK, and the joint role of PppHIJ in propionylation – literature precedence for multi-protein complexes catalyzing acylation does exist (52). Future studies should also elucidate the exact mechanisms by which pseudovibriamides modulate gene transcription.

## MATERIALS AND METHODS

### General cultivation conditions

*P. brasiliensis* Ab134 was cultivated at 30°C on BD Difco Marine Agar 2216 (MA) or in BD Difco Marine Broth 2216 (MB) for 18–20 h unless otherwise noted. Chloramphenicol (8 µg/mL) and kanamycin (200 µg/mL) were used for mutant selection as appropriate. *E. coli* strains were cultured in BD Difco Luria Broth (LB) or on LB agar for 18–20 h. Chloramphenicol (25 µg/mL) and kanamycin (50 µg/mL) were used for mutant selection as appropriate. *E. coli* DH5α(λpir) was used for propagation of pDS132-based vectors and *E. coli* SM10(λpir) for conjugation with Ab134. *E. coli* DH5α was used for propagation of pSEVA227M-based or pAM4891-based vectors and *E. coli* S17-1 for conjugation. All strains were cryo-preserved in 20% glycerol [*v/v*] at −80°C.

### Plasmid construction

Plasmids used in this study are summarized in Table S2. Oligonucleotide primers (Table S8) were synthesized by Sigma-Aldrich. Vector pDS132 was used to construct plasmids for in-frame deletion, pSEVA227M for promoter probe studies, and pAM4891 for genetic complementation (53–55). See Supplementary Information for details.

### In-frame deletion and complementation

Mutants were generated by in-frame deletion via homologous recombination (Fig. S1). pDS132 or pYD004-based suicide vectors were first transformed into *E. coli* SM10(λpir), which was used as the conjugation donor for transferring vectors into Ab134. The detailed conjugation protocol can be found under Supplementary Information. Obtained clones were analyzed by two parallel PCRs to identify single crossover (SCO) colonies (Fig. S1). Confirmed SCO clones were streaked onto non-selective MA plates and incubated overnight at 30°C. MA containing 5% sucrose was used for counterselection of the vector. Chloramphenicol-sensitive clones were analyzed by PCR to confirm the gene replacement (Fig. S2). All pAM4891-based complementation vectors (49, 55) were first electroporated into *E. coli* S17-1, which was used as the conjugation donor for introducing vectors into Ab134 mutants. Except for the pYDcompD, which was electroporated directly into the *∆pppD* mutant. Kanamycin (200 µg/mL) was used to select the incoming plasmid and exconjugants or transformants were confirmed by plasmid extraction and restriction digest.

### Swarming assays

The protocol used was as we previously reported (10) with only minor modifications as detailed under Supplementary Information.

### Pseudovibriamide extraction and analysis

Pseudovibriamides were extracted in two ways based on culture conditions. (A) Swarming agar cultures were extracted with one volume (20 mL) of methanol by sonicating for 1 h, after which the extract was filtered through filter paper. (B) Liquid cultures were extracted by first capturing metabolites using XAD-7HP resin (Sigma-Aldrich), and then extracting the resin with methanol. Methanol extracts were dried under reduced pressure and stored at −20°C until analysis. See Supplementary Information for further details and for pseudovibriamide extraction from cell pellet and supernatant. Extracts were analyzed by either dried droplet MALDI-ToF MS or by UPLC-QToF-MS/MS as described under Supplementary Information.

### RNA extraction and transcriptomics analyses

The overall scheme is summarized in Fig. S60. RNA was isolated from 24 h triplicate cultures using the RiboPure-Bacteria kit (Thermo Fisher Scientific) following the manufacturer’s instructions. Purified RNA (Table S9) with RNA integrity number >8.0 was sent to SeqCenter (Pittsburgh, MA) for bacterial rRNA depletion RNA sequencing using a NextSeq2000 sequencer giving 2 × 51 bp reads. Demultiplexing, quality control, and adapter trimming was performed with bcl-convert (v3.9.3). Raw Illumina reads of each sample were mapped to the Ab134 whole genome (NCBI accession number GCA_018282095.1) using Geneious. Expression levels (FPKM, RPKM, and TPM) were calculated (all contigs at once) with the option that ambiguously mapped reads were counted as partial matches. Pairwise differential expression analysis was performed by using DESeq2 package in Geneious or using TPM values to calculate FC directly (FDR was calculated using Benjamini-Hochberg function in R studio). The eggnog-mapper V2.0 was used to perform functional annotation based on Clusters of Orthologous Genes (COGs) (56). Further analysis of DE gene function was performed using Basic Local Alignment Search Tool (BLAST) (57) Phyre2 protein fold recognition (25) and contrastive learning-enabled enzyme annotation (CLEAN) (26). See Supplementary Information for further details.

## Data Availability

RNA-seq data were deposited at NCBI (accession code GSE263005).

## SUPPLEMENTAL MATERIAL

File S1 mbio.03115-24-s0001.xlsx Raw transcription data.

Supplemental Information mbio.03115-24-s0002.pdf Supplemental methods, results, tables, and figures.

ASM does not own the copyrights to Supplemental Material that may be linked to, or accessed through, an article. The authors have granted ASM a non-exclusive, world-wide license to publish the Supplemental Material files. Please contact the corresponding author directly for reuse.
