# Supplementary material for: Pseudovibriamides from Pseudovibrio marine sponge bacteria promote flagellar motility via transcriptional modulation
Source: mBio. 2024 Dec 27;16(2):e03115-24. doi: 10.1128/mbio.03115-24 (PMC11796379; doi:10.1128/mbio.03115-24)
Supplement: Supplemental Information — Supplemental methods, results, tables, and figures. [file mbio.03115-24-s0002.pdf]

Supplementary Information

Pseudovibriamides from *Pseudovibrio* marine sponge bacteria promote flagellar motility via transcriptional modulation

Yitao Dai,[1] Vitor Lourenzon,[1] Laura P. Ióca,[1,2] Dua Al-Smadi,[1] Lydia Arnold,[1] Ian McIntire,[1] Roberto G. S. Berlinck,[2] Alessandra S. Eustáquio[1*]

[1]Department of Pharmaceutical Sciences and Center for Biomolecular Sciences, College of Pharmacy, University of Illinois at Chicago, Chicago, IL 60607, USA

[2]Instituto de Química de São Carlos, Universidade de São Paulo, CP 780, CEP 13560-970, São Carlos, SP 13565-905, Brazil

Table of Contents

Table of Contents 2
Supplementary Results 7
Identifying DE genes potentially involved with reduced swarming motility 7
Materials and Methods 7
Chemicals and general experimental procedures 7
Plasmid construction. 8
General. 8
Construction of pYD004 to be used as vector to construct deletion plasmids. 8
Construction of pYD00A to generate ΔpppA strain. 9
Construction of pYD00D to generate ΔpppD strain. 9
Construction of pVL00E to generate ΔpppE strain. 10
Construction of pVL00F to generate ΔpppF strain. 10
Construction of pYD00G to generate ΔpppG strain. 10
Construction of pYD00K to generate ΔpppK strain. 11
Construction of pYDORF1 to generate ΔpppL(=ORF1) strain. 11
Construction of pLA002 to generate ΔpppM (=ORF2) strain. 11
Construction of pLI04 to generate ΔpppH strain. 12
Construction of pLI05 to generate ΔpppI strain. 12
Construction of pLI07 to generate ΔpppJ strain. 12
Construction of pYDluxR to generate ΔluxR strain. 13
Construction of pYDproA for the autoregulation test. 13
Construction of pDS00H for pppH trans-complementation. 14
Construction of pDS00I for pppI trans-complementation. 14
Construction of pDS00J for pppJ trans-complementation. 14
Construction of pVL00K for pppK trans-complementation. 14
Construction of pYDcompE for pppE trans-complementation. 14
Construction of pYDcompF for pppF trans-complementation. 15
Construction of pYDcompG for pppG trans-complementation. 15
Construction of pYDcompD for pppD trans-complementation. 15
Construction of pYDrhaR for luxR overexpression. 16
Scarless in-frame deletion in Ab134. 16
Trans-complementation via conjugation. 18
Trans-complementation via electroporation. 18
Swarming assays. 19
Measurement of swarming area using ImageJ. 20
Pseudovibriamides extraction. 20
Pseudovibriamides extraction from cell supernatant and pellet. 20
MALDI-ToF MS sample preparation and analysis. 21
UPLC-QToF-MS/MS sample preparation and analysis. 21
RNA extraction and transcriptomics. 22
Bioinformatics. 24
Visualization of Ab134 using transmission electron microscopy (TEM). 24

Growth curves. **25**
Growth curves of the wild type and ΔpppA/D/E/G/K mutants in Figure 4. 25
Growth curves of the wild type, the ΔpppE ΔluxR mutant, the ΔpppD mutant, and strains with pYDrhaR. 25
Pseudovibriamide autoregulation assay. **26**
Tables 27
Table S1. Putative functions of proteins encoded in the *ppp* BGC. **27**
Table S2. Summary of plasmids used in this study. **29**
Table S3. Potential mutations identified in the Δ*pppG* mutant compared to the wild type. **31**
Table S4. Potential mutations identified in the Δ*pppK* mutant compared to the wild type. **32**
Table S5. List of DE genes in Δ*pppA/E* mutants but NDE in Δ*pppD* mutant compared to the wild type. **33**
Table S6. List of specified DE genes in Δ*pppA/E* mutants but inversely regulated in Δ*pppD* mutant compared to wild type. **34**
Table S7. The swarming areas* of the wild type, the Δ*pppE* mutant, and the Δ*pppE* Δ*luxR* mutant after 96-hour incubation. **35**
Table S8. Oligonucleotide primers used in this study. **36**
Table S9. Summary of RNA sample quality used in this study. **39**
Figures 40
Figure S1. Scheme of in-frame deletion via homologous recombination. **40**
Figure S2. Gel electrophoresis images of scarless in-frame deletion mutants verified by PCR. **42**
Figure S3. MALDI-ToF MS analysis of wild type and Δ*pppA* mutants **44**
Figure S4. Comparison of PC1 production and MS/MS fragmentation between wild type and mutants in triplicates. **45**
Figure S5. Comparison of PC2 or PC3 production and MS/MS fragmentation between wild type and mutants in triplicates. **47**
Figure S6. Comparison of PA, PB, and PC production between wild type and Δ*pppL* mutants. **48**
Figure S7. Comparison of PA, PB, and PC production between wild type and Δ*pppM* mutants. **49**
Figure S8. MALDI-ToF MS analyses of wild type and Δ*pppM* mutant. **51**
Figure S9. Comparison of PA1 production and MS/MS fragmentation between wild type and mutants in triplicates. **53**
Figure S10. Comparison of PB1 production and MS/MS fragmentation between wild type and mutants in triplicates **55**
Figure S11. Comparison of PB2 or PB3 production and MS/MS fragmentation between wild type and mutants in triplicates. **57**

Figure S12. Comparison of PA, PB, and PC production between Δ*pppD* mutant, wild type and genetically complemented Δ*pppD* mutant. 58

Figure S13. Comparison of PA, PB, and PC production between Δ*pppE* mutant, wild type and genetically complemented Δ*pppE* mutant. 59

Figure S14. Comparison of PA, PB, and PC production between Δ*pppF* mutant, wild type and genetically complemented Δ*pppF* mutant. (A) 60

Figure S15. Comparison of PA1 production and MS/MS fragmentation between wild type, Δ*pppH,* Δ*pppI,* Δ*pppJ* mutants, and genetically complemented strains 61

Figure S16. Comparison of PB1 production and MS/MS fragmentation between wild type, Δ*pppH,* Δ*pppI,* Δ*pppJ* mutants, and genetically complemented strains 63

Figure S17. Comparison of PB2 or PB3 production and MS/MS fragmentation between wild type, Δ*pppH,* Δ*pppI,* Δ*pppJ* mutants, and genetically complemented strains. 65

Figure S18. Comparison of PC1 production and MS/MS fragmentation between wild type, Δ*pppH,* Δ*pppI,* Δ*pppJ* mutants, and genetically complemented strains 67

Figure S19. Comparison of PC2 or PC3 production and MS/MS fragmentation between wild type, Δ*pppH,* Δ*pppI,* Δ*pppJ* mutants, and genetically complemented strains. 69

Figure S20. Comparison of depropionylated PBs and PCs production between wild type and Δ*pppK* mutant 70

Figure S21. MALDI-ToF MS analyses of wild type and biological triplicates of Δ*pppK* mutants. 71

Figure S22. Comparison of MS/MS fragmentation between PBs from wild type and depropionylated PBs from Δ*pppK* mutant. 72

Figure S23. Comparison of MS/MS fragmentation between PCs and depropionylated PCs. 74

Figure S24. Comparison of PA, PB, and PC production between Δ*pppK* mutant, wild type and genetically complemented ΔpppK mutant. 75

Figure S25. Comparison of the second adenylation (A) domains from PppB and PppD. 76

Figure S26. The workflow of pseudovibriamides extraction from cell pellet and supernatant 77

Figure S27. Comparison of pseudovibriamide export ratio between wild type and transporter mutants. 78

Figure S28. EIC of PA1 from supernatant and pellet extracts of genetically complemented Δ*pppG* strain, wild type, and Δ*pppG* mutant. 79

Figure S29. EIC of PB1 from supernatant and pellet extracts of genetically complemented Δ*pppG* strain, wild type, and Δ*pppG* mutant. 80

Figure S30. EIC of PB2 or PB3 from supernatant and pellet extracts of genetically complemented Δ*pppG* strain, wild type, and Δ*pppG* mutant. 81

Figure S31. EIC of PC1 from supernatant and pellet extracts of genetically complemented ΔpppG strain, wild type, and Δ*pppG* mutant. 82

Figure S32. EIC of PC2 or PC3 from supernatant and pellet extracts of genetically complemented ∆*pppG* strain, wild type, and ∆*pppG* mutant. 83

Figure S33. EIC of PA1 from supernatant and pellet extracts of wild type and ∆*pppL* mutant. 84

Figure S34. EIC of PB1 from supernatant and pellet extracts of wild type and ∆*pppL* mutant. 85

Figure S35. EIC of PB2 or PB3 from supernatant and pellet extracts of wild type and ∆*pppL* mutant. 86

Figure S36. EIC of PC1 from supernatant and pellet extracts of wild type and ∆*pppL* mutant. 87

Figure S37. EIC of PC2 or PC3 from supernatant and pellet extracts of wild type and ∆*pppL* mutant. 88

Figure S38. A summary of statistical analyses performed for pseudovibriamides export ratio comparison between ∆*pppG* mutant and complemented ∆*pppG* mutant............... 89

Figure S39. A summary of statistical analyses performed for pseudovibriamides export ratio comparison between ∆*pppG* mutant and wild type. 90

Figure S40. A summary of statistical analyses performed for pseudovibriamides export ratio comparison between ∆*pppL* mutant and wild type. 91

Figure S41. Swarming assay results of ∆*pppA* and *pppA::neo* mutants compared to the wild type. 92

Figure S42. Swarming assay results of ∆*pppD* and *pppD::neo* mutants compared to the wild type. 93

Figure S43. Swarming assay results of ∆*pppH,* ∆*pppI,* and ∆*pppJ* mutants compared to the wild type. 94

Figure S44. Triplicate swarming assay results of ∆*pppA,* ∆*pppD,* ∆*pppE,* ∆*pppG,* and ∆*pppK* mutants compared to the wild type. 95

Figure S45. Swarming assay results of ∆*pppE* mutant compared to the wild type and the genetically complemented strain. 96

Figure S46. Swarming assay results of ∆*pppG* and ∆*pppK* mutants compared to the wild type and genetically complemented strains. 97

Figure S47. Swarming assay results of ∆*pppF* mutant compared to the wild type........ 99

Figure S48. Swarming assay results of ∆*pppL* mutant compared to the wild type...... 100

Figure S49. Swarming assay results of ∆*pppM* mutant compared to the wild type. 101

Figure S50. Transmission Electron Microscopy of wild type and mutants. 103

Figure S51. Volcano plots of differentially expressed genes identified between ∆*pppA* and ∆*ppp*D mutants and the wild type using DESeq2.. 105

Figure S52. Identification of P_{pppA} based on RNA-Seq data. 106

Figure S53. P_{pppA} autoregulation assay.. 107

Figure S54. COG classification of DE genes in ∆*pppA/E* mutants filtered using NDE genes of ∆*pppD* mutant compared to the wild type. 108

Figure S55. COG classification of DE genes reversely regulated in Δ*pppA/E* mutants and the Δ*pppD* mutant compared to the wild type. 109

Figure S56. Swarming assay results of strains with inducible vector pYDrhaR. 110

Figure S57. Growth of strains with inducible vector pYDrhaR as measured by OD_{600}. 111

Figure S58. Swarming assay results of the Δ*pppE* Δ*luxR* and Δ*luxR* mutants compared to the wild type and the Δ*pppE* mutant. 112

Figure S59. Growth of the Δ*pppE* mutant, and the Δ*pppE* Δ*luxR* mutant as measured by OD_{600} for 24 hours. 113

Figure S60. Workflow from bacterial culture to transcriptomic analysis. 114

References 115

Supplementary Results

Identifying DE genes potentially involved with reduced swarming motility. Considering scenario 1 (the Δ*pppD* mutant possess the same set of genes unaffected compared to the wild type and regulated inversely in Δ*pppA* and Δ*pppE* mutants compared to the wild type), two of the three genes identified in the signal transduction [T] category not described in the main text are described here. One is predicted to encode YscD (locus tag: KGB56_13360), a type III secretion system (T3SS) inner membrane ring subunit (1). However, it is not near any genes that encode the remaining components of T3SS, and there is no direct relationship reported in the literature between YscD and swarming motility that we could find. The second gene (locus tag: KGB56_13020) upregulated in [T] is predicted to encode an amino acid periplasmic substrate binding protein of an ABC transporter. The substrate could be arginine, lysine, or histidine based on BLAST results. As described in the main text, upregulation of ABC transporters has been observed in non-swarming cells (2).

Materials and Methods

Chemicals and general experimental procedures. Chemicals and enzymes were acquired from Sigma-Aldrich, VWR, BD, and Thermo Fisher Scientific, unless otherwise noted. Restriction enzymes were purchased from New England Biolabs (NEB). Phusion™ high-fidelity DNA polymerase (Thermo Fisher Scientific, referred as PHF polymerase later) and DreamTaq Master Mix 2× (Thermo Fisher Scientific) were used for PCR amplification. T4 DNA ligase (Thermo Fisher Scientific) was used for cloning. Oligonucleotide primers were designed in Geneious and synthesized by Sigma-Aldrich (see **Table S8** for detailed sequences). A Bio-Rad T100 thermal cycler was used for PCR reactions. The general PCR parameters for PHF polymerase reactions were: 60 s at 98°C; 30 cycles of 98°C for 10 s, Ta for 30 s, and 72°C for 30 s/kb; final extension

for 10 mins at 72°C; and an indefinite hold at 10°C, and for DreamTaq polymerase reactions were: 3 mins at 95°C; 30 cycles of 95°C for 30 s, Ta for 30 s, and 72°C for 1 min/kb; final extension for 10 mins at 72°C; and an indefinite hold at 10°C. Genomic DNA was isolated using GenElute™ Bacterial Genomic DNA Kit (Sigma-Aldrich). ZymoPURE Plasmid Miniprep kits were used to extract plasmid DNA. The RiboPure™-Bacteria kit (Thermo Fisher Scientific) was used to extract RNA. Zymo Research Clean and Concentrator kit was used for DNA cleanup. To confirm the accuracy of plasmid constructs, Sanger sequencing was performed by UIC's genome research core and whole plasmid sequencing by Primordium labs.

Plasmid construction.

<u>*General.*</u> Ab134 genomic DNA was used as template for amplification of homology arm pairs (construction of pYD00A/D/G/K, pVL00E/F, pLI04/5/7, pYDORF1 and pLA002), a fragment containing predicted promoter of *pppA* (pYDproA), and fragments containing *pppG/H/I/J/K/E/F* genes respectively (pDS00H/I/J, pYDcompE/F/G, pVL00K). Phusion™ high-fidelity DNA polymerase (Thermo Scientific, referred as PHF polymerase later) was used for PCR amplification using primers listed in **Table S8** at 500 nM each unless otherwise noted. After restriction digests with the appropriate enzymes (**Table S8**), T4 DNA Ligase (Thermo Scientific) was used to ligate inserts and vector backbones. NEBuilder® HiFi DNA Assembly Master Mix was used for building pYDcompE/F/G.

<u>*Construction of pYD004 to be used as vector to construct deletion plasmids.*</u> PHF polymerase and the primer pair lacZ_F / lacZ_R (Ta = 63°C, 30 cycles) were used to amplify the *lacZ*α gene from pSET152 (Bierman, 1992). PCR products were digested with SpeI-HF on both ends and ligated into pDS132 digested with XbaI. LB agar (20 mL) supplemented with chloramphenicol (25 µg/mL) was treated with 5-bromo-4-chloro-3-indolyl-β-D-galactopyranoside (X-Gal, 0.8 mg/mL) and used to select *E. coli* DH5α (λpir) clones (blue colonies) that were further confirmed by using restriction digestion (EcoRV) and Sanger sequencing.

Construction of pYD00A to generate ΔpppA strain. One 895-bp upstream homology arm and one 888-bp downstream homology arm were amplified using PHF DNA polymerase and primer pairs pppA_Up_F / pppA_Up_R (Ta = 62°C, 30 cycles) and pppA_Down_F / pppA_Down_R (Ta = 62°C, 30 cycles) respectively. Both homology arms were assembled using splicing by overlap extension-PCR (SOE-PCR) with the following reaction (50 µL): purified homology arms (~100 ng), 0.2 mM dNTP mix (Thermo Scientific), 3% DMSO, 0.02 U/µL PHF polymerase with 1× PHF buffer, and molecular biology grade water. Extension reactions were performed using following parameters: 60 s at 98°C; 5 cycles of 98°C for 10 s, 60°C for 30 s, and 72°C for 90 s; and a final hold at 10°C. The primer pair pppA_Up_F / pppA_Down_R (Ta = 62°C) was added to the extension reaction before starting amplification: 60 s at 98°C; 30 cycles of 98°C for 10 s, 62°C for 30 s, and 72°C for 90 s; and a final extension for 10 mins at 72°C. The PCR product was digested with XbaI and SalI-HF and ligated into pYD004 digested with the same enzymes (located inside *lacZ*α gene). LB agar (20 mL) supplemented with chloramphenicol (25 µg/mL) was treated with X-Gal (0.8 mg/mL) to select *E. coli* DH5α (λpir) clones (white colonies as *lacZ*α gene was interrupted) that were further confirmed by using restriction digestion (SmaI) and Sanger sequencing.

Construction of pYD00D to generate ΔpppD strain. One 801-bp upstream homology arm and one 799-bp downstream homology arm were amplified using PHF DNA polymerase and primer pairs pppD_Up_F / pppD_Up_R (Ta = 61°C, 30 cycles) and pppD_Down_F_2 / pppD_Down_R (Ta = 61°C, 30 cycles) respectively. Both homology arms were assembled using SOE-PCR as described for pYD00A above. The same parameters of pYD00A were used for the first extension step except for the annealing temperature at 58°C for 30 s. The same parameters were also used for the second, amplification step except that the primer pair added was pppD_Up_F / pppD_Down_R (Ta = 61°C). The same restriction digestion, ligation, screening, and verification procedures of pYD00A were used.

Construction of pVL00E to generate ΔpppE strain. One 740-bp upstream homology arm and one 730-bp downstream homology arm were amplified using PHF DNA polymerase and primer pairs pppE_Up_F / pppE_Up_R (Ta = 62°C, 30 cycles) and pppE_Down_F / pppE_Down_R (Ta = 61°C, 30 cycles) respectively. Both homology arms were assembled using SOE-PCR as described for pYD00A above. The same parameters of pYD00A were used for the first thermal cycler extension. The same parameters were also used for the second amplification step except that the primer pair added was pppE_Up_R / pppE_Down_F (Ta = 61°C). Same restriction digestion, ligation, screening, and verification procedures of pYD00A were used.

Construction of pVL00F to generate ΔpppF strain. One 873-bp upstream homology arm and one 870-bp downstream homology arm were amplified using PHF DNA polymerase and primer pairs pppF_Up_F / pppF_Up_R (Ta = 63°C, 30 cycles) and pppF_Down_F / pppF_Down_R (Ta = 63°C, 30 cycles) respectively. Both homology arms were assembled using SOE-PCR with the reaction mentioned in pYD00A construction. The same parameters of pYD00A were used for the first thermal cycler extension except for the annealing temperature at 61°C for 30 s. The same parameters were also used for the second amplification step except for the primer pair added was pppF_Up_R / pppF_Down_F (Ta = 61°C). The PCR product was digested with XbaI and XhoI (compatible with SalI-HF) due to the presence of a SalI restriction site in the upstream homology arm. The same ligation, screening, and verification procedures of pYD00A were used.

Construction of pYD00G to generate ΔpppG strain. One 892-bp upstream homology arm and one 925-bp downstream homology arm were amplified using PHF DNA polymerase and primer pairs pppG_Up_F / pppG_Up_R (Ta = 61°C, 30 cycles) and pppG_Down_F / pppG_Down_R (Ta = 62°C, 30 cycles) respectively. Both homology arms were assembled using SOE-PCR with the reaction mentioned for pYD00A. The same parameters of pYD00A were used for the first thermal cycler extension except for the annealing temperature at 59°C for 30 s. The same parameters were also used for the second amplification step except for the primer pair added was pppG_Up_R / pppG_Down_F (Ta = 61°C). The same restriction digestion, ligation, and screening

procedures of pYD00A were used. Positive clones were further confirmed by using restriction digestion (NcoI) and Sanger sequencing.

Construction of pYD00K to generate ∆pppK strain. One 977-bp upstream homology arm and one 921-bp downstream homology arm were amplified using PHF DNA polymerase and primer pairs pppK_Up_F / pppK_Up_R (Ta = 64°C, 30 cycles) and pppK_Down_F / pppK_Down_R (Ta = 66°C, 30 cycles) respectively. Both homology arms were assembled using SOE-PCR with the reaction mentioned for pYD00A. The same parameters of pYD00A were used for the first thermal cycler extension except that the annealing temperature was 64°C for 30 s. The same parameters were also used for the second amplification step except for the primer pair added was pppK_Up_R / pppK_Down_F (Ta = 66°C). The same ligation, screening, and verification procedures of pYD00G were used.

Construction of pYDORF1 to generate ∆pppL(=ORF1) strain. One 752-bp upstream homology arm and one 765-bp downstream homology arm were amplified using PHF DNA polymerase and primer pairs ORF1_Up_F_2 / ORF1_Up_R (Ta = 61°C, 30 cycles) and ORF1_Down_F_2 / ORF1_Down_R (Ta = 61°C, 30 cycles) respectively. Both homology arms were assembled using SOE-PCR with the reaction mentioned for pYD00A. The same parameters of pYD00A were used for the first thermal cycler extension except that the annealing temperature at 58°C for 30 s. The same parameters were also used for the second amplification step except for the primer pair added was ORF1_Up_F_2 / ORF1_Down_R (Ta = 61°C). Same ligation, and screening procedures of pYD00A were used. Positive clones were further confirmed by using double restriction digestion (XbaI and SalI-HF) and Sanger sequencing.

Construction of pLA002 to generate ∆pppM (=ORF2) strain. One 919-bp upstream homology arm and one 980-bp downstream homology arm were amplified using PHF DNA polymerase and primer pairs ORF2_Up_F / ORF2_Up_R (Ta = 61°C, 30 cycles) and ORF2_Down_F / ORF2_Down_R (Ta = 61°C, 30 cycles) respectively. Both homology arms were assembled using SOE-PCR with the reaction mentioned for pYD00A. The same parameters of pYD00A were used

for the first thermal cycler extension except for the annealing temperature at 61°C for 30 s. The same parameters were also used for the second amplification step except for the primer pair added was ORF2_Up_F / ORF2_Down_R (Ta = 61°C). Same restriction digestion, ligation, screening, and verification procedures of pYDORF1 were used.

Construction of pLI04 to generate ∆pppH strain. One 951-bp upstream homology arm and one 905-bp downstream homology arm were amplified using PHF DNA polymerase and primer pairs oLI32 / oLI33 (Ta = 57°C, 30 cycles) and oLI34 / oLI35 (Ta = 60°C, 30 cycles) respectively. Both homology arms were assembled using SOE-PCR with the reaction mentioned for pYD00A. The same parameters of pYD00A were used for the first thermal cycler extension except for the annealing temperature at 62°C for 30 s. The same parameters were also used for the second amplification except for the primer pair added was oLI33 / oLI34 (Ta = 59°C). The PCR product was digested with XbaI and ligated into pDS132 digested with XbaI. LB agar (20 mL) supplemented with chloramphenicol (25 µg/mL) was used to select *E. coli* DH5α (λpir) clones that were further confirmed by using double restriction digestion (NcoI and BamHI) and Sanger sequencing.

Construction of pLI05 to generate ∆pppI strain. One 979-bp upstream homology arm and one 980-bp downstream homology arm were amplified using PHF DNA polymerase and primer pairs oLI36 / oLI37 (Ta = 61°C, 30 cycles) and oLI38 / oLI39 (Ta = 61°C, 30 cycles) respectively. Both homology arms were assembled using SOE-PCR with the reaction mentioned for pYD00A. The same parameters of pLI04 were used for the first thermal cycler extension. The same parameters were also used for the second amplification step except that the primer pair added was oLI37 / oLI38 (Ta = 61°C). The same restriction digestion, ligation, screening, and verification procedures of pLI04 were used. Clones were further confirmed by using restriction digestion (EcoRV-HF) and Sanger sequencing.

Construction of pLI07 to generate ∆pppJ strain. One 1014-bp upstream homology arm and one 994-bp downstream homology arm were amplified using PHF DNA polymerase and primer pairs

oLI40 / oLI41 (Ta = 61°C, 30 cycles) and oLI46 / oLI43 (Ta = 61°C, 30 cycles) respectively. Both homology arms were assembled using SOE-PCR with the reaction mentioned for pYD00A. The same parameters of pLI04 were used for the first thermal cycler extension. Theame parameters were also used for the second amplification step except that the primer pair added was oLI41 / oLI46 (Ta = 61°C). The PCR product was digested with XbaI and SacI and ligated into pDS132 also digested with XbaI and SacI. LB agar (20 mL) supplemented with chloramphenicol (25 µg/mL) was used to select *E. coli* DH5α (λpir) clones that were further confirmed by using double restriction digestion (NcoI and BamHI) and Sanger sequencing. The same ligation and screening of pLI04 were used.

Construction of pYDluxR to generate ΔluxR strain. One 838-bp upstream homology arm and one 839-bp downstream homology arm were amplified using PHF DNA polymerase and primer pairs luxR_Up_F / luxR_Up_R (Ta = 61°C, 30 cycles) and luxR_Down_F / luxR_Down_R (Ta = 61°C, 30 cycles) respectively. A 5322-bp fragment excluding the *lacZα* gene was amplified from linearized pYD004 (XbaI digested) using PHF DNA polymerase and the primer pair YD_4_F / YD_4_R (Ta = 61°C, 30 cycles). Modified Gibson assembly was used for cloning (NEBuilder® HiFi DNA Assembly Master Mix) (Gibson, 2009). LB agar (20 mL) supplemented with chloramphenicol (25 µg/mL) was treated with X-Gal (0.8 mg/mL) to select *E. coli* DH5α (λpir) clones (white colonies as *lacZ*α gene was interrupted) that were further confirmed by using restriction digestion (SmaI) and whole plasmid sequencing.

Construction of pYDproA for the autoregulation test. The promoterless pSEVA227M plasmid vector was used as the backbone with a monomeric superfolder green fluorescent protein (msfGFP) inserted as a reporter. One 216-bp upstream fragment of *pppA* gene containing a predicted promoter and a ribosome binding site was amplified using PHF DNA polymerase and the primer pair Pro_pppA_F / Pro_pppA_R (Ta = 57°C, 30 cycles). Both PCR product and pSEVA227M were digested with XbaI and HindIII, and the promoter fragment was inserted into a position 14-bp upstream of the *msfGFP* gene. After ligation, the mixture was chemically

transformed into *E. coli* DH5α, and positive clones were selected by using LB agar (20 mL) supplemented with kanamycin (50 µg/mL) and further confirmed by using restriction digestion (AseI) and Sanger sequencing.

Construction of pDS00H for pppH trans-complementation. The 1141-bp fragment containing the *pppH* gene and ribosomal binding site (RBS) was amplified using PHF DNA polymerase and the primer pair pppH_comp_F / pppH_comp_R (Ta = 60°C, 30 cycles). The PCR product was digested with NdeI and BamHI (compatible with BglII) and ligated into pAM4891 digested with NdeI and BglII (located inside *the GFP* gene; 68% of the *GFP* gene was retained downstream of the insertion site). LB agar (20 mL) supplemented with kanamycin (50 µg/mL) was used to select *E. coli* DH5α clones with lower intensity of green fluorescence and were further confirmed by using restriction digestion (SmaI) and Sanger sequencing.

Construction of pDS00I for pppI trans-complementation. The 831-bp fragment containing the *pppI* gene and RBS was amplified using PHF DNA polymerase and the primer pair pppI_comp_F / pppI_comp_R (Ta = 59°C, 30 cycles). The PCR product was digested with NdeI and BglII and ligated into the same sites of pAM4891. The same screening and verification procedures of pDS00H were used.

Construction of pDS00J for pppJ trans-complementation. The 723-bp fragment containing the *pppJ* gene and RBS was amplified using PHF DNA polymerase and the primer pair pppJ_comp_F / pppJ_comp_R (Ta = 60°C, 30 cycles). The same restriction digestion, ligation, screening, and verification procedures of pDS00I were used.

Construction of pVL00K for pppK trans-complementation. The 1017-bp fragment containing the *pppK* gene and RBS was amplified using PHF DNA polymerase and the primer pair pppK_F / pppK_R (Ta = 64°C, 30 cycles). The same restriction digestion, ligation, and screening procedures of pDS00I were used. Whole plasmid sequencing was used for verification.

Construction of pYDcompE for pppE trans-complementation. The 831-bp fragment containing the *pppE* gene and RBS was amplified using Q5 High-fidelity DNA polymerase and primer pairs

pppE_comp_F / pppE_comp_R (Ta = 69°C, 30 cycles) following the manufacturer's instructions. A 7057-bp fragment excluding the *GFP* gene was amplified from pAM4891 using PHF DNA polymerase and the primer pair pAM4891_GA_F / pAM4891_GA_R (Ta = 61°C, 30 cycles). Gibson assembly was used for cloning (NEBuilder® HiFi DNA Assembly Master Mix) (Gibson, 2009). LB agar (20 mL) supplemented with kanamycin (50 µg/mL) was used to select *E. coli* DH5α clones with no green fluorescence that were further confirmed by using restriction digestion (XhoI and EcoRV) and whole plasmid sequencing.

Construction of pYDcompF for pppF trans-complementation. The 834-bp fragment containing the *pppF* gene and RBS was amplified using Q5 High-fidelity DNA polymerase and primer pairs pppF_comp_F / pppF_comp_R (Ta = 63°C, 30 cycles). The same 7057-bp fragment from pAM4891 as described above for pYDcompE was used for cloning. The same screening procedure was used to pick positive clones, which were further confirmed by using restriction digestion (BamHI and EcoRV) and whole plasmid sequencing.

Construction of pYDcompG for pppG trans-complementation. The 1280-bp fragment containing the *pppG* gene and RBS was amplified using Q5 High-fidelity DNA polymerase and primer pairs pppG_comp_F / pppG_comp_R (Ta = 65°C, 30 cycles). The same 7057-bp fragment from pAM4891 as described above for pYDcompE was used for cloning. The same screening procedure was used to pick positive clones, which were further confirmed by using restriction digestion (SmaI) and whole plasmid sequencing.

Construction of pYDcompD for pppD trans-complementation. The 8994-bp fragment containing the *pppD* gene and RBS was amplified using PHF DNA polymerase and primer pairs pppD_GA_F / pppD_GA_R (Ta = 64°C, 30 cycles). The same 7057-bp fragment from pAM4891 as described above for pYDcompE was used for cloning. The same screening procedure was used to pick positive clones, which were further confirmed by using restriction digestion (XmnI) and whole plasmid sequencing.

Construction of pYDrhaR for *luxR* overexpression. The 843-bp fragment containing the *luxR* gene (KGB56_18890) and RBS was amplified using PHF DNA polymerase and the primer pair luxR_rha_F / luxR_rha_R (Ta = 61°C, 30 cycles). The PCR product was digested with NdeI and HindIII and ligated into pJeM1 digested with NdeI and HindIII (the whole *GFP* gene was lost). LB agar (20 mL) supplemented with kanamycin (50 µg/mL) was used to select *E. coli* DH5α clones, after which clones were replica plated onto LB agar with kanamycin and LB agar with both kanamycin (50 µg/mL) and 0.1% *w/v* L-rhamnose. Because we previously attempted to clone this *luxR* gene with a constitutive promoter and only got clones with stop codon mutations, we expected the correct construct would only grow on the plate without L-rhamnose due to the toxicity of LuxR to *E. coli* DH5α. Positive clones were further confirmed by using restriction digestion (AseI) and whole plasmid sequencing.

Scarless in-frame deletion in Ab134. Before conjugation, all pDS132 or pYD004 based suicide vectors were chemically transformed into *E. coli* SM10(λpir) using heat shock at 42°C for 45 s. For conjugation, fresh cultures of Ab134 or *E. coli* SM10(λpir) with suicide vectors were started by inoculating 400 µL of overnight seed culture into 20 mL of MB or 20 mL of LB containing chloramphenicol (25 µg/mL), respectively. The cultures were incubated at 30°C, 200 rpm, until an Optical Density at 600nm (OD_{600nm}) between 0.4 and 0.6 was reached. Cells were harvested by centrifugation at 4°C, 4000 rpm. Ab134 cells were resuspended into 2 mL of MB. *E. coli* SM10(λpir) cells were washed twice with 20 mL of LB to remove antibiotics prior to resuspension in 2 mL of LB. Cell suspensions of Ab134 and *E. coli* SM10(λpir) (500 µL each) were mixed in a 1.5-mL Eppendorf tube, and then plated evenly onto two MA plates. Negative control plates were prepared by plating only *E. coli* SM10(λpir) (250 µL) or only Ab134 (250 µL) onto MA plates. Plates were incubated at 30°C for 16-20 hours. A loopfull (10 µL loop) of cells were then streaked onto MA (20 mL) containing chloramphenicol (8 µg/mL) to select for the incoming plasmid and carbenicillin (50 µg/mL) to selectively kill *E. coli*. The wild type Ab134 strain was used as parent

to generate mutants, except for Δ*pppD* only, for which fresh culture of Ab134 *pppD::neo* was used as the conjugation recipient (MB containing kanamycin 200 µg/mL). A loopfull of cells were then streaked onto MA (20 mL) containing kanamycin (200 µg/mL), chloramphenicol (8 µg/mL) to select for the incoming plasmid, and carbenicillin (50 µg/mL) to selectively kill *E. coli*. Positive clones (sticky single colonies) were analyzed by two parallel PCRs to identify single crossover (SCO) colonies using two primer pairs P1 / P3 and P4 / P2 (**Fig. S1**). The primer pairs (**Table S8**) used for each mutant were P1: TP_pppA_F / P3: pppA_Down_R (Ta = 50°C) and P4: pppA_Up_F / P2: TP_pppA_R (Ta = 51°C) (*pppA* SCO); TP_pppD_F_2 / pppD_Down_R (50°C) and pppD_Up_F / TP_pppD_R_4 (49°C) (*pppD* SCO); E_TP1_F / E_TP1_R (52°C) and E_TP2_F / E_TP2_R (52°C) (*pppE* SCO); F_TP1_F / F_TP1_R (52°C) and F_TP2_F / F_TP2_R (52°C) (*pppF* SCO); TP_pppG_F / pppG_Up_R (48°C) and pppG_Down_F / TP_pppG_R (49°C) (*pppG* SCO); TP_pppK_F / pppK_Up_R (53°C) and pppK_Down_F / TP_pppK_R (55°C) (*pppK* SCO); TP_ORF1_F_2 / ORF1_Down_R (49°C) and ORF1_Up_F_2 / TP_ORF1_R (51°C) (*pppL* SCO); and TP_ORF2_F / ORF2_Down_R (47°C) and ORF2_Up_F / TP_ORF2_R (50°C) (*pppM* SCO). For testing *pppH/I/J* SCO, P3 (pDS132_R) and P4 (pDS132_F) were located on pDS132. P1 and P2 were: oLI38 and JD_P2 (*pppH SCO*); oLI45 and oLI33 (*pppI SCO*); and oLI45 and oLI37 (*pppJ SCO*). For testing *luxR* SCO, luxR_DCO_R (located on genome) and luxR_SCO_F (located on ligation junction) were used for only one PCR reaction. All SCO PCR reactions were performed using the reaction mixture (20 µL): genomic DNA (~100 ng), 10 µL DreamTaq PCR Master Mix 2× (Thermo Scientific), both primers (250 nM each), and molecular biology grade water. Reactions were performed using following parameters: 60 s at 95°C; 30 cycles of 95°C for 30 s, Ta °C for 30s, and 72°C for 1min/kb; and a final extension for 10 mins at 72°C.

Once SCO colonies were confirmed, they were streaked evenly onto non-selective MA plates to allow double crossover (DCO) events to happen. Plates were incubated overnight at 30°C. A loopful of cells were then streaked for single colonies onto MA containing 5% sucrose (pDS132-based vectors have a *sacB* counterselection marker) and incubated for one to two days

at 30°C. Obtained single colonies were replica plated onto MA or MA containing chloramphenicol (8 µg/mL) except for potential Δ*pppD* colonies, which were replica plated onto MA or MA containing kanamycin (200 µg/mL), or MA containing chloramphenicol (8 µg/mL).

Chloramphenicol-sensitive clones (or kanamycin / chloramphenicol-sensitive clones for Δ*pppD*) were analyzed by colony PCR or PCR using genomic DNA (in case of poor colony PCR result) to confirm the gene replacement using the primer pair P1 / P2 (20 µL reaction, DreamTaq polymerase) for SCO verification. Thermocycling conditions were the same as those of SCO except that for the colony PCR the initial denaturation was extended from 60 s to 5 min at 95°C.

Trans-complementation via conjugation. All pAM4891-based complementation vectors except pYDcompD were first electroporated into *E. coli* S17-1, which was then used as the conjugation donor for introducing vectors into Ab134. The same conjugation protocol as for gene deletions was used, except the recipient strain is not Ab134 but the corresponding mutants. Selection of exconjugants was carried out on MA (20 mL) containing kanamycin (200 µg/mL) to select for the incoming plasmid and carbenicillin (50 µg/mL) to selectively kill *E. coli*. Positive clones (sticky single colonies) were inoculated into MB (5 mL) containing kanamycin (200 µg/mL) for plasmid extraction after overnight growth at 30°C. Plasmids from complemented strains were confirmed using the same restriction digestion protocol used in the plasmid construction step.

Trans-complementation via electroporation. To prepare the Δ*pppD* electro-competent cells, fresh cultures of the Δ*pppD* mutant were started by inoculating 400 µL of overnight seed cultures into 20 mL of marine broth in a 250 mL Erlenmeyer flask. Cultures were incubated at 30°C, 200 rpm, until an optical density at 600 nm (OD_{600}) between 0.4 and 0.6 was reached. Cells were harvested by centrifugation at 4°C, 4000 rpm. Cells were washed four times with 20 mL of ice-cold, filter-sterilized 250 mM sucrose solution. Washed cells were resuspended in the leftover sucrose solution, and 100 µL of the cell suspension were aliquoted for electroporation.

Fresh electro-competent cells were used. For each electroporation, 100 ng of the plasmid pYDcompD was added to the cell suspension and mixed gently. The electroporation was carried out in a 0.2 cm ice-cold electroporation cuvette using an electroporator set to 1.8 kV. The expected time constant is around 5.2 – 5.4ms. Electro-shocked cells were immediately mixed with 1 mL ice-cold marine broth and recovered at 30°C for 1 – 2 hrs. Marine agar plates containing kanamycin (200 µg/mL) were used to select positive clones. Clones were confirmed using the same restriction digestion (XmnI) protocol used in the plasmid construction step.

The same protocol was also applied to introduce pYDrhaR into the ∆*pppD* mutant and the wild type. Marine agar plates containing kanamycin (150 µg/mL) were used to select positive clones. Clones were confirmed using the same restriction digestion (AseI) protocol used in the plasmid construction step.

Swarming assays. The protocol used was as we previously reported (3) with slight modifications (the OD_{600} of cultures used was 0.4 instead of 1.0). For convenience, we provide details here. A swarming agar with lower surface tension was prepared by mixing MB (LOT: 2124239) and 0.5% (*w/v*) of Eiken agar (Eiken Chemical CO., Japan). The autoclaved agar medium was equilibrated at 50°C for 2 h and aliquoted onto Petri dishes (exactly 20 mL), which were air dried for 20 mins in a laminar flow hood. Fresh cultures of Ab134 and mutants with OD_{600nm} of 0.4-0.6 were normalized to OD_{600nm} of 0.4. To perform the swarming assays, 5 µL of normalized cultures were carefully dropped on the center of the plate. Plates were air dried again for 10 min and incubated at 30°C (a water dish was placed in the incubator to maintain humidity. Pictures were taken daily for 4 days (when the swarming front had reached the plate edge). The swarming phenotype was apparent after two days. For the induction of pYDrhaR, 0.1% L-rhamnose (200 µL of a 10% *w/v* L-rhamnose stock solution) was added to 20 mL of swarming agar. The induction was verified by detecting GFP using pJeM1.

Measurement of swarming area using ImageJ. The diameter of Petri dishes (8.5 cm) was used as the internal scale to normalize measurements. Thus, pixels were converted to units in centimeters. Due to the irregular shape of the swarming pattern, the freehand selection option was used to select the swarming area.

Pseudovibriamides extraction. Pseudovibriamides were extracted in two ways based on culture conditions. (A) swarming agar plates were broken into pieces and extracted with one volume (20 mL) of methanol by sonicating for 1 h, after which the extract was filtered through filter paper. These extracts from WT, Δ*pppK*, and Δ*pppM* were analyzed with MALDI-ToF MS. (B) For all mutants and complemented Δ*pppH/I/J* strains, 1 mL of normalized seed culture based on OD_{600nm} readings was added to 250 mL Erlenmeyer flasks containing 25 mL of MB in triplicates. After a 24-hour incubation at 30˚C, 200 rpm, exactly 10 mL of liquid cultures were extracted by first adding 5% w/v XAD-7HP resins (Sigma-Aldrich) directly to the culture, and shaking for 1 h at 500 rpm. The resin was collected by filtration and mixed with one volume methanol (10 mL), after which is was shaken for 1 h at 500 rpm. Methanol extracts were dried by the SpeedVac concentrator (Thermo Scientific) and stored at -20°C.

Pseudovibriamides extraction from cell supernatant and pellet. In addition to the whole culture extraction of Δ*pppG* and Δ*pppL* mutants, which were predicted to encode membrane transport proteins, supernatant and cell pellet were separated for extraction as shown in **SI Figure S26**. For the comparison between Δ*pppG* and WT, 1 mL of normalized seed culture based on OD_{600nm} readings was added to 250 mL Erlenmeyer flasks with 50 mL of MB in triplicates and grown for 48 hours, 200 rpm. For the comparison between Δ*pppL* and WT, the same inoculation procedure was used but in quadruplicates. The same method as extracting liquid culture was used to extract exactly 30 mL of the supernatant portion, and the same method as extracting swarming agar plates was used to extract the corresponding cell pellet portion.

MALDI-ToF MS sample preparation and analysis. An Autoflex Speed LRF mass spectrometer (Bruker Daltonics) equipped with a SmartBeamTM-II laser (355 nm) was used for analysis. Dried-droplet analyses were performed in positive reflectron mode (Laser intensity: 70%-80%; Reflector: 15-18×; RepRate: 2000 Hz; ion source 1 voltage: 19 kV; ion source 2 voltage: 16.55 kV; lens voltage: 8.3 kV; mass range: 700 to 1500 Da). Samples (dissolved in methanol) and the calibrant were mixed in 1:1 ratio with universal matrix and 1 µL of the mixture was applied onto a designated spot on a MALDI ground-steel target plate (Bruker Daltonics). The universal matrix was prepared by mixing 1:1 of α- cyano-4-hydroxycinnamic acid (CHCA) and 2,5-dihydroxybenzoic acid (2,5-DHB) (Sigma-Aldrich) solubilized in 78:22 MeCN in water with 0.1% TFA. External calibration was made with a Bruker Daltonics peptide calibration standard using "PeptideCalibStandard mono" as the mass control list. Data was acquired by flexControl software v. 3.4.135.0 and analyzed using flexAnalysis software v. 3.4.

UPLC-QToF-MS/MS sample preparation and analysis. UPLC-MS/MS analyses were performed on a Bruker Elute LC system coupled to a Bruker Compact QToF mass spectrometer with electrospray ionization (ESI), using positive mode with following parameters: capillary voltage of 4500 V, nebulizer of 4.0 Bar, dry gas (N_2) flow of 12.0 L/min, source temperature of 225°C, scanning range from 50 to 3000 Da, and collision energy of 10 eV. Internal calibration was conducted with 5 mM sodium formate solution (Sigma-Aldrich) charged by the lock-mass probe at a flow rate of 40 µL/min. UPLC was performed at 0.5 mL/min through an Agilent Poroshell 120 EC-C18 UPLC column (2.1 × 50 mm × 1.9 µm) at 40°C. A solvent phase of aqueous 10% acetonitrile for 1 min (equilibration), 10% to 100% acetonitrile for 6 min (gradient), 100% acetonitrile for 2 min (wash), and 100% to 10% acetonitrile (re-equilibration) for 1 min was used. Both acetonitrile and water were LC-MS grade supplemented with 0.1% formic acid (v/v). Crude extracts were resuspended in LC-MS grade methanol and diluted to a final concentration of 1 mg/mL. Diluted samples were filtered using 4mm PTFE syringe filter 0.2 µm (Thermo Scientific)

and injected into the system as 2 µL. Data was acquired and analyzed using Bruker Compass DataAnalysis software v. 5.1.

The complementation of the ∆*pppD* mutant was verified using equipments from the Integrated Molecular Structure Education and Research Center (IMSERC) at Northwestern. Samples were injected on a 1290 Infinity II UHPLC System (Agilent Technologies Inc., Santa Clara, CA, USA) on a Poroshell 120 EC-C18 column (1.9 µm, 50 × 2.1 mm) (Agilent Technologies Inc., Santa Clara, CA, USA). The column was maintained at 30 °C, with a constant flow rate of 0.500 mL/min, and C-18 chromatography was performed using a gradient of mobile phase A (water with 0.1% formic acid) and mobile phase B (100% acetonitrile with 0.1% formic acid). The gradient program was as follows: 0–1 min, 10% B; 1–6 min, 10–99% B; 6–8 min, hold at 99% B; 8–8.10 min, 99–10% B; 8.10–11 min, hold at 10% B. Auto MS/MS acquisition in positive ion mode was performed using an Agilent 6545 quadrupole time-of-flight mass spectrometer with a JetStream ionization source. The source conditions were: gas temperature, 300 °C; drying gas flow, 12 L/min; nebulizer pressure, 45 psi; sheath gas temperature, 400 °C; sheath gas flow, 11 L/min; VCap voltage, 3500 V; fragmentor voltage, 150 V; and skimmer voltage, 65 V. Data was acquired and analyzed using Agilent MassHunter Qualitative Analysis Navigator B.08.00.

For analyzing supernatant and cell pellet extracts, the area under curve (AUC) of each pseudovibriamide was obtained by using the extracted ion chromatogram (EIC) function. The ratio of AUC of pseudovibriamides in supernatant to AUC of pseudovibriamides in pellet was compared between Ab134 and ∆*pppG/L* mutants. The equation used for calculating export ratio is shown below:

$$Export\ ratio_{PA/B/C} = \frac{AUC_{supernatant}}{AUC_{pellet}}$$

RNA extraction and transcriptomics. We first attempted to perform transcriptomics analysis using cells grown on swarming agar plates, but we observed too much variation in the RNAseq

data because cells collected from the swarm front or the swarm middle, for example, will be at different growth stages (and have different transcriptomes). Because the Δ*pppA* and Δ*pppE* mutants have reduced swarming it is very difficult to discern the different swarming regions and obtain cells from swarming regions that are equivalent to the wild type. Thus, the RNAseq results from the triplicate samples were not tight, precluding meaningful comparisons between the different groups. We then switched to liquid cultures, with the overall scheme summarized in **Fig. S60**. Using liquid cultures not only helped with the technical issues of working with agar cultures but it is also physiologically relevant to the effect of pseudovibriamides because both flagella-mediated motility phenotypes – swarming and swimming – are affected by pseudovibriamides as we reported in our previous paper (3).

Triplicate cultures of Ab134 and Δ*pppA/D/E* mutants were started by adding 1 mL of standardized overnight seed cultures (OD_{600nm} ~ 1.0) to 50 mL of MB in 250 mL Erlenmeyer flasks and incubating at 30°C, 200 rpm. After 24 h or 48 h, 5 mL of culture of each triplicate was taken for metabolite analysis (extracted with 5% XAD-7HP resin [w/v]). Meanwhile, another 0.25 mL of culture, standardized based on OD_{600nm} between samples so that an equivalent number of cells was collected, was centrifuged at 16,000 ×g for 2 min. Cell pellets were mixed with 0.25 mL of RNA-later® (Sigma-Aldrich) and stored at -20°C. RNA was isolated using the RiboPure™-Bacteria kit (Thermo) following manufacturer's instructions. The purified RNA was pre-screened by using a NanoDrop® ND-1000 UV-Vis Spectrophotometer. Although both 24 h and 48 h samples had all pseudovibriamides produced based on LC-MS/MS chromatograms, the RNA of 24 h samples had better quality and quantity than those of 48 h samples based on Nanodrop readings (**SI Table S9**). Thus, 24 h RNA samples were submitted to UIC's Genome Research Core for further quality control using Qubit® dsDNA HS (High Sensitivity) and Qubit® RNA BR (Broad-Range) assay kits (Thermo Fisher Scientific), and TapeStation 4200 (Agilent) (**SI Table S9**). Qualified samples (RIN, RNA integrity number > 8.0) were sent to SeqCenter (Pittsburgh, MA) for bacterial rRNA depletion

RNA Sequencing. At SeqCenter, samples were DNAse treated with Invitrogen DNAse kit (Thermo Fisher Scientific). Library preparation was performed using Illumina's Stranded Total RNA Prep Ligation with Ribo-Zero Plus kit and 10 bp IDT for Illumina indices. Sequencing was done on a NextSeq2000 instrument giving 2×51bp reads. Demultiplexing, quality control, and adapter trimming was performed with bcl-convert (v3.9.3). Raw Illumina reads of each sample were mapped to the Ab134 whole genome (NCBI accession number GCA_018282095.1) by using Geneious. Expression levels (FPKM, RPKM, and TPM) were calculated (all contigs at once) by using Geneious with the option that ambiguously mapped reads were count as partial matches. Pairwise differential expression analysis was performed by using DESeq2 package in Geneious or using TPM values to calculate FC directly (FDR was calculated using Benjamini-Hochberg function in R studio). The principal component analysis (PCA) plot, volcano plot, and heatmap were generated by using ggplot2 and ComplexHeatmap functions in R studio.

Bioinformatics. To check Δ*pppK* and Δ*pppG* strains for mutations, whole genome sequencing data (Illumina reads) were trimmed by BBDuk Trimmer and mapped to reference in Geneious Prime. Low coverage regions were anotated using Geneious Prime. Single nucleotide polymorphisms (SNPs) are found using Geneious Prime with the cutoff that the minimum vairant frequency is 0.90. SNPs that fell into low coverage regions were excluded.

Visualization of Ab134 using transmission electron microscopy (TEM). TEM sample preparation was modified from a previous study (Grossart, 2000). Seed cultures were prepared by growing 50 µL of cryo-preserved culture in 5 mL of MB at 30°C overnight (18-20 h). Cultures were preserved by adding 4% paraformaldehyde to a final concentration of 2%. 300-mesh copper TEM grids with Carbon-stabilized Formvar supports (FCF-300CU, Electron Microscopy Science) were used. Bacteria cells were adsorbed to TEM grids by floating the grids Formvar side down

on an undiluted drop of culture (30 µL) for 5 min. Grids loaded with bacteria were stained by submerging into 1% phosphotungstic acid (30 µL) for 30 s and then were submerged in aliquots of Milli-Q water for rinsing three times (10 s each). Extra liquid of each step was removed gently by using filter paper. The grids were examined with a JEOL JEM-1400 Flash TEM (accelerating voltage of 80kV) at UIC's Electron Microscopy Core the same day of preparation.

Growth curves.

Growth curves of the wild type and ΔpppA/D/E/G/K mutants in Figure 4. The growth of each strain was monitored for 48 h using a microtiter plate method. Seed cultures were prepared by growing 50 µL of cryo-preserved culture in 5 mL of MB at 30°C overnight (18-20 h). Seed cultures were diluted in MB to OD_{600nm} of 0.05. Standardized cultures (200 µL each well, 12 replicates for each strain) were aliquoted into BIOFLOAT® 96-well plates (faCellitate, U-bottom, inert, cell-repelling surface, sterile) using a multi-channel pipettor. Border wells were filled with MB (blank). Mutants were divided into two plates due to limited wells (Ab134 was in both plates as the control). BioTek Synergy HTX multimode reader (Agilent) was used to incubate plates at 30°C and shake the plate orbitally at speed of 425 cycle per minute (3 mm). OD_{600nm} reading of each well was taken every 30 min (98 reads in total over 48 h). All readings were normalized by subtracting the readings from the MB blank wells. Half of the replicates without outlier readings were used to calculate average values and standard deviations for plotting the final growth curve.

Growth curves of the wild type, the ΔpppE ΔluxR mutant, the ΔpppD mutant, and strains with pYDrhaR. The growth of each strain was monitored with the same method but within different plates and a different plate reader. Falcon™ 96-well, non-treated, flat-bottom microplates were used. Plates were incubated at 30°C and shaken linearly at a speed of 887 revolutions per minute (amplitude 1 mm) using an Infinite M Plex plate reader (Tecan). OD_{600} readings of each well were taken every 30 min. All readings were normalized by subtracting the readings from the MB blank wells.

Pseudovibriamide autoregulation assay. All strains (8 in total) were generated by introducing pYDproA (pSEVA227M based) or pSEVA227M (negative control) vectors into wild type/Δ*pppA*/Δ*pppD*/Δ*pppE* via using *E. coli* S17-1 as the conjugation donor. Before conjugation, both vectors were respectively transformed into *E. coli* S17-1 using electroporation (100 ng plasmid, 2.5kV, time constant 5 ms, 200Ω). For conjugation, the same protocol as conjugation of in-frame deletion vectors was used. Kanamycin (200 µg/mL) was used to select for the incoming plasmid and carbenicillin (50 µg/mL) to selectively kill *E. coli*. Plasmid DNA from kanamycin-resistant, single colonies were isolated and confirmed by using restriction digestion (AseI). Cultures of the eight strains were started by adding enough of overnight seed cultures (based on OD_{600nm} readings) to 50 mL of MB with kanamycin (200 µg/mL) in 250 mL Erlenmeyer flasks so that the starting OD_{600nm} was standardized to 0.05. Cultures were incubated at 30°C, 200 rpm. Starting from T0, 300 µL of each culture was diluted into 2700 µL of salted PBS buffer (extra 8 g NaCl per liter added), and aliquoted into Corning Costar® 96-well plate (black plate, clear bottom with lid, 200 µL of diluted culture per well, 12 wells for each strain). Measurements of both OD_{600nm} and GFP fluorescence were taken every 2 hours in a Tecan infinite® 200Pro plate reader (488 nm excitation and 520 nm emission). After cultures reached OD_{600nm} 0.5 of (~6 hours), measurements were taken every one hour until 14 hours. The dilution ratio was raised from 1:10 to 1:50 (60 µL of each culture added to 3940 µL salted PBS buffer) for measurements taken at 24 hours, 26 hours, 28 hours, 38 hours, and 48 hours due to overly high fluorescence levels. All flasks were kept shaking for 48 hours except when samples were taken. The data was plotted using Excel.

Tables

Table S1. Putative functions of proteins encoded in the *ppp* BGC.

Gene	Protein size (a. a.)	BLAST analysis	Phyre2 analysis	Notes
pppA<br>*pppB*<br>*pppC*	2766<br>3468<br>1373	Non-ribosomal peptide synthetase		Biosynthesis of peptides as modular enzymes. (4)
pppD	2993	Polyketide synthase/ non-ribosomal peptide synthetase hybrid		Biosynthesis of hybrid polyketide-peptides as modular enzymes. (5)
pppE	256	4'-phosphopantetheinyl transferase		Transfer of phosphopantetheine from coenzyme A to ACP/PCP. (6)
pppF	258	Type II thioesterase		Cleavage of aberrant intermediates or regenerating mis-acylated thiol groups. (7, 8)
pppG	414	Major Facilitator Superfamily (MFS) transporter; PTR2[a]		Translocation of small solutes (di/tripeptides)[a] across the membrane. (9)
pppH	349	SGNH hydrolase superfamily	Acetyltransferase or acyltransferase	The SGNH hydrolase family includes esterases and acetyltransferases. (10)
pppI	238	Hypothetical protein; SGNH domain containing protein[a]	*O*-acetyltransferase AlgX/J (11)	
pppJ	211	Hypothetical protein	Hydrolase (low confidence)	
pppK	304	Fe(II)-oxoglutarate dependent dioxygenase		Reactions include hydroxylation. (12)
pppL	411	MFS transporter; drug/proton antiporter[a]		Same family as PppG; efflux of a variety of drugs and toxic compounds using proton gradient. (13)
pppM	158	GCN5-related *N*-acetyltransferase		Acetyl transfer from acetyl-CoA to a substrate. (14)
pppN	346	ABC transporter periplasmic substrate-binding protein; AfuA[a]		Recognition and delivery of a specific substrate for a specific ABC transporter. (15)
pppO	462	Sensor histidine kinase		Part of a two-component system (TCS);

			Phosphorylation of response regulator transcription factor in response to signals. (16)
pppP	222	DNA-binding response regulator	Part of TCS. DNA binding and transcriptional control upon phosphorylation. (17)

[a]Predicted by Conserved Domains in BLAST: PTR2, di/tripeptide permease; and AfuA, periplasmic component of Fe^{3+} transport system. Low confidence, <20%; MFS, major facilitator superfamily; ABC, ATP-binding cassette; AlgJ/X, alginate *O*-acetyltransferase; and ACP/PCP, acyl/peptidyl carrier protein.

Table S2. Summary of plasmids used in this study.

Plasmid	Description of features	Application	Reference
pDS132	*ori* R6K (replication only in *E. coli* λpir); RP4 (conjugation system); Cm^R; *sacB* (a counter selectivion marker utilizing sucrose); MCS	Vector used to construct pYD004.	(18)
pYD004	Based on pDS132*; ori* R6K; RP4; Cm^R; *lacZ*α (blue-white screening); *sacB*; MCS	pYD004 was constructed by introducing *lacZ*α from pSET152 into pDS132. Vector used to construct in-frame deletion plasmids.	pSET152 (19) pYD004 (this study)
pYD00A<br>pYD00D<br>pYD00G<br>pYD00K	Based on pYD004; upstream and downstream homology arms of *pppA/D/G/K* cloned inside *lacZ*α	Delivery of replacement alleles for in-frame deletion into Ab134 cells.	This study
pVL00E<br>pVL00F	Based on pYD004; upstream and downstream homology arms of *pppE/F* cloned inside *lacZ*α	Delivery of replacement alleles for in-frame deletion into Ab134 cells.	This study
pLI04<br>pLI05<br>pLI07	Based on pDS132; upstream and downstream homology arms of *pppH/I/J* ligated in the MCS	Delivery of replacement alleles for in-frame deletion into Ab134 cells.	This study
pYDORF1	Based on pYD004; upstream and downstream homology arms of *pppL* cloned inside *lacZ*α	Delivery of replacement alleles for in-frame deletion into Ab134 cells.	This study
pLA002	Based on pYD004; upstream and downstream homology arms of *pppM* cloned inside *lacZ*α	Delivery of replacement alleles for in-frame deletion into Ab134 cells.	This study
pYDluxR	Based on pYD004; upstream and downstream homology arms of *luxR* cloned inside *lacZ*α	Delivery of replacement alleles for in-frame deletion into Δ*pppE* and WT cells.	This study
pSEVA227M	*oriT* RK2 (*oriV, trfA;* broad host range); Km^R; *msfGFP* (promoterless)	Source of the GFP reporter gene and negative control in promoter-probe studies.	(20)
pYDproA	Based on pSEVA227M but with *msfGFP* under the control of the *pppA* promoter)	Test pseudovibriamide autoregulation of the P_{pppA} promoter.	This study
pAM4891	*Ori* RSF1010 (*oriV/T*, *repA/B,* broad host range); Km^R; *gfp; tac* promoter	Source of the backbone in construction of conjugatable, genetic complementation plasmids.	(21)
pVL00K	Based on pAM4891 but with *pppK* (native RBS included) inserted into *gfp*	Genetic complementation of the Δ*pppK* mutant.	This study
pYDcompD<br>pYDcompE<br>pYDcompF<br>pYDcompG	Based on pAM4891; *gfp* replaced with *pppD/E/F/G* (native RBS included)	Genetic complementation of Δ*pppD/E/F/G* mutants, respectively.	This study
pDS00H<br>pDS00I	Based on pAM4891 but with *pppH/I/J* (native RBS included) inserted into *gfp*	Genetic complementation of Δ*pppH/I/J* mutants, respectively.	This study

pDS00J			
pJeM1	*ori* pBBR1 (*oriV, rep, mob;* broad host range); Km^R; *gfp*; *rhaR, rhaS, rhaB* inducible promoter (L-rhamnose)	Source of the backbone in construction of the LuxR overexpression plasmid.	(22)
pYDrhaR	Based on pJeM1 but with *luxR* (native RBS included inserted into *gfp*	Overexpression of LuxR in the wild type and the Δ*pppD* mutant	This study

**ori*, origin of replication; *rep*, replication gene; R, resistance; Cm, chloramphenicol; Km, kanamycin; *msfGFP*, monomeric superfolder green fluorescent protein gene; and RBS, ribosome binding site.

Table S3. Potential mutations identified in the Δ*pppG* mutant compared to the wild type.

Start (bp)	End (bp)	Nucleotide Change	Polymorphism Type	Variant %	Amino Acid Change	Locus tag	Protein	Protein Effect
**Δ*pppG* chromosome**								
3018287	3018286	+C	Insertion	100.0%			Intergenic<br><br>Upstream: Hypothetical protein (KGB56_ 13510)<br><br>Downstream: Hypothetical protein (KGB56_ 13515)	
3018285	3018285	A -> G	SNP (transition)	100.0%				
3018282	3018282	A -> T	SNP (transversion)	100.0%				
3018280	3018280	A -> C	SNP (transversion)	100.0%				
3018275	3018278	AAAC -> TCTG	Substitution	100.0%				
3018272	3018273	AA -> GC	Substitution	100.0%				
3018261	3018261	A -> T	SNP (transversion)	100.0%				
3018251	3018254	AATA -> GCAT	Substitution	100.0%				
3018247	3018247	A -> T	SNP (transversion)	100.0%				
3018244	3018245	AA -> GC	Substitution	100.0%				
3018209	3018209	T -> G	SNP (transversion)	100.0%				
3018270	3018270	G -> T	SNP (transversion)	99.5%				
3018256	3018259	ACAA -> TGGC	Substitution	99.5%				
3018249	3018249	A -> T	SNP (transversion)	99.5%				
3018288	3018290	AAA -> TCT	Substitution	99.4%				
3018263	3018263	A -> T	SNP (transversion)	99.0%				
3018265	3018268	TATC -> GCAT	Substitution	97.3%				
550563	550563	T -> C	SNP (transition)	99.6%	T -> A	KGB56 _02520	Hypothetical protein	Substitution
3960649	3960649	G -> T	SNP (transversion)	99.3%	T -> K	KGB56 _17835	CheB	Substitution
**Plasmid #4**								
3951	3951	A -> G	SNP (transition)	100.0%	K -> R	KGB56 _26455	PAPS	Substitution
54820	54819	+GCTTGGG	Insertion	98.6%		KGB56 _26670	Hypothetical protein	Frame Shift

*SNP, single nucleotide polymorphism; Variant %, frequency of variant; only SNPs with frequency of variant ≥ 90% are included; CheB, chemotaxis response regulator protein-glutamate methylesterase; PAPS, phosphoadenosine phosphosulphate reductase; SNPs without protein effect are in intergenic spaces; T, threonine; A, alanine; K, lysine; R, arginine.

Table S4. Potential mutations identified in the ∆*pppK* mutant compared to the wild type.

Start (bp)	End (bp)	Nucleotide Change	Polymorphism Type	Variant %	Amino Acid Change	Locus tag	Protein	Protein Effect
**∆*pppK* chromosome**								
3018287	3018286	+C	Insertion	100.0%			Intergenic<br><br>Upstream: Hypothetical protein (KGB56_ 13510)<br><br>Downstream: Hypothetical protein (KGB56_ 13515)	
3018285	3018285	A -> G	SNP (transition)	100.0%				
3018280	3018280	A -> C	SNP (transversion)	100.0%				
3018263	3018263	A -> T	SNP (transversion)	100.0%				
3018251	3018254	AATA -> GCAT	Substitution	100.0%				
3018247	3018247	A -> T	SNP (transversion)	99.7%				
3018244	3018245	AA -> GC	Substitution	99.7%				
3018270	3018270	G -> T	SNP (transversion)	99.6%				
3018265	3018268	TATC -> GCAT	Substitution	99.6%				
3018261	3018261	A -> T	SNP (transversion)	99.6%				
3018249	3018249	A -> T	SNP (transversion)	99.6%				
3018209	3018209	T -> G	SNP (transversion)	99.5%				
3018275	3018278	AAAC -> TCTG	Substitution	99.2%				
3018272	3018273	AA -> GC	Substitution	99.2%				
3018288	3018290	AAA -> TCT	Substitution	99.1%				
3018282	3018282	A -> T	SNP (transversion)	99.1%				
3018256	3018259	ACAA -> TGGC	Substitution	98.9%				
2746853	2746864	GTGGTCGTGGT C -> none	Deletion (tandem repeat)	100.0%	HDHD -> none	KGB56 _12280	CobW	Deletion
550563	550563	T -> C	SNP (transition)	99.7%	T -> A	KGB56 _02520	Hypothetical protein	Substitution
3960649	3960649	G -> T	SNP (transversion)	99.4%	T -> K	KGB56 _17835	CheB	Substitution

*SNP, single nucleotide polymorphism; Variant %, frequency of variant; only SNPs with frequency of variant ≥ 90% are included; CobW, cobalamin biosynthesis protein; CheB, chemotaxis response regulator protein-glutamate methylesterase; SNPs without protein effect are in intergenic spaces; H, histidine; D, aspartic acid; T, threonine; A, alanine; K, lysine.

Table S5. List of DE genes in Δ*pppA/E* mutants but NDE in Δ*pppD* mutant compared to the wild type.

Description	Locus tag	A log$_2$FC	A FDR	E log$_2$FC	E FDR	D log$_2$FC	$\lvert\Delta$log$_2$FC$\rvert$ A_D	$\lvert\Delta$log$_2$FC$\rvert$ E_D	COG
**Upregulation**									
hypothetical protein CDS	KGB56_25290	2.06	0.0053	1.57	0.0039	0.90	1.16	0.66	S
VOC family protein CDS	KGB56_25440	1.93	0.0062	1.89	0.0012	-0.55	2.48	2.44	E
FAD-dependent oxidoreductase CDS	KGB56_25450	1.78	0.0051	1.70	0.0034	-0.61	2.39	2.31	CH
TRAP transporter substrate-binding protein CDS	KGB56_25435	1.78	0.0057	1.59	0.0056	-0.91	2.69	2.50	G
cupin domain-containing protein CDS	KGB56_07995	1.57	0.0035	2.15	0.0017	-0.32	1.90	2.47	S
ABC transporter substrate-binding protein CDS	KGB56_22975	1.53	0.0061	1.16	0.0073	0.64	0.89	0.52	P
cyclic nucleotide-binding/CBS domain-containing protein CDS	KGB56_23400	1.41	0.0053	1.28	0.0067	-0.22	1.63	1.50	T
amino acid ABC transporter ATP-binding protein CDS	KGB56_12375	1.27	0.0077	1.16	0.0070	0.55	0.72	0.61	E
SIS domain-containing protein CDS	KGB56_04810	1.25	0.0029	1.27	0.0055	0.34	0.91	0.92	M
hypothetical protein CDS	KGB56_25995	1.19	0.0028	1.13	0.0040	0.39	0.80	0.74	S
cadherin-like domain-containing protein CDS	KGB56_13365	1.07	0.0041	1.28	0.0037	0.49	0.58	0.79	Q
type III export protein YscD CDS	KGB56_13360	1.02	0.0063	1.21	0.0023	0.22	0.80	0.99	T
**Downregulation**									
transglutaminase-like cysteine peptidase CDS	KGB56_14760	-3.04	0.0044	-2.93	0.0039	-0.49	2.55	2.43	S
peptidoglycan DD-metalloendopeptidase family protein CDS	KGB56_11125	-2.12	0.0009	-2.13	0.0009	-0.51	1.61	1.61	M
methyltransferase domain-containing protein CDS	KGB56_20140	-2.11	0.0010	-1.85	0.0010	-0.96	1.15	0.89	Q
argininosuccinate synthase CDS	KGB56_20790	-1.93	0.0012	-1.12	0.0094	-0.43	1.50	0.69	E
mutT CDS	KGB56_20150	-1.35	0.0087	-1.33	0.0054	-0.56	0.79	0.77	L

* A, Δ*pppA* mutant; E, Δ*pppE* mutant; D, Δ*pppD* mutant; FC, fold change when compared to wild type; $\lvert\Delta$log$_2$FC$\rvert$, absolute values obtained from log$_2$FC of Δ*pppA/E* mutants minus log$_2$FC of Δ*pppD* mutant; COG, cluster of orthologous groups.

Table S6. List of specified DE genes in ∆*pppA/E* mutants but inversely regulated in ∆*pppD* mutant compared to wild type.

Description	Locus tag	A log_2FC	A FDR	E log_2FC	E FDR	D log_2FC	COG
**Upregulation**							
hypothetical protein CDS	KGB56_00075	2.20	0.0079	2.19	0.0029	-3.90	S
HlyD family type I secretion periplasmic adaptorsubunit CDS	KGB56_04835	2.08	0.0030	2.33	0.0046	-2.39	M
basic amino acid ABC transporter substrate-binding protein CDS	KGB56_13020	1.95	0.0041	1.24	0.0021	-1.31	ET
NUDIX hydrolase CDS	KGB56_03310	1.68	0.0073	1.10	0.0023	-1.17	L
type I secretion system permease/ATPase CDS	KGB56_04840	1.68	0.0008	2.26	0.0048	-2.67	V
LuxR family transcriptional regulator CDS	KGB56_18890	1.54	0.0050	1.48	0.0006	-2.78	K
YifB family Mg chelatase-like AAA ATPase CDS	KGB56_21835	1.48	0.0037	1.85	0.0006	-3.28	O
hypothetical protein CDS	KGB56_24120	1.48	0.0067	2.04	0.0021	-2.30	S
lipid-A-disaccharide synthase N-terminal domain-containing protein CDS	KGB56_12395	1.25	0.0084	1.47	0.0031	-3.88	S
PaaI family thioesterase CDS	KGB56_22365	1.13	0.0027	1.51	0.0073	-3.84	Q
type III polyketide synthase CDS	KGB56_24430	1.04	0.0070	1.57	0.0096	-4.24	Q
MoxR family ATPase CDS	KGB56_16960	1.02	0.0061	1.12	0.0028	-2.24	S

* A, ∆*pppA* mutant; E, ∆*pppE* mutant; D, ∆*pppD* mutant; FC, fold change when compared to wild type; COG, cluster of orthologous groups.

Table S7. The swarming areas* of the wild type, the Δ*pppE* mutant, and the Δ*pppE* Δ*luxR* mutant after 96-hour incubation.

Strain replicate	Area (cm^2)	Average (cm^2)	Standard deviation (cm^2)
Δ*pppE 1*	40.846	40.443	0.349
Δ*pppE 2*	40.239		
Δ*pppE 3*	40.245		
Δ*pppE* Δ*luxR 1*	54.433	51.209	5.501
Δ*pppE* Δ*luxR 2*	54.337		
Δ*pppE* Δ*luxR 3*	44.857		
WT 1	55.613	55.868	0.780
WT 2	56.744		
WT 3	55.247		
Δ*luxR* 1	56.391	56.147	0.269
Δ*luxR* 2	55.859		
Δ*luxR* 3	56.192		

* An internal scale was set based on the diameter (8.5 cm) of plate.

Table S8. Oligonucleotide primers used in this study.

Primer name	Sequence from 5' to 3'	Additional Info
lacZ gene amplification from pSET152		
lacZ_F	*GG*ACTAGTTCTCCGACCTGATGCAGC	SpeI
lacZ_R	*GG*ACTAGTGCCTTTGAGTGAGCTGATACC	SpeI
pppA gene in-frame deletion and verification		
pppA_Up_F	*ACGC*GTCGACAGCTACCTTCAACAACCTCG	SalI
pppA_Up_R	TACGTTGGAAAGACTAAACTCACC	
pppA_Down_F	GGTGAGTTTAGTCTTTCCAACGTAGCCGAAGAATTTTCCTGACG	
pppA_Down_R	*GC*TCTAGACTCACATGCAAACTCTCTTACC	XbaI
TP_pppA_F	AGGAAGAACGTGAGAGGC	
TP_pppA_R	ATCTCTTCCAGCTCTTTACGG	
pppD gene in-frame deletion and verification		
pppD_Up_F	*ACGC*GTCGACAGATGCACAAGACAACCTG	SalI
pppD_Up_R	ATGTGAGATAGTGCTGTTCATG	
pppD_Down_F_2	CATGAACAGCACTATCTCACATGTGCTCAGTGTCATGGAC	
pppD_Down_R	*GC*TCTAGAGATGTCTCAACCCGCTAC	XbaI
TP_pppD_F_2	TAAACCCAACAATCGAAGCG	
TP_pppD_R_4	AGCAAAGTGGAAGAATGGAC	
pppE gene in-frame deletion and verification		
pppE_Up_F	CCAGCGAAGAGGAACATCAAAATGTTGCCACTTCCCTCTC	
pppE_Up_R	*GC*TCTAGATTGGTGATCAGGTGAACGTG	XbaI
pppE_Down_F	*ACGC*GTCGACTGACCCTCACCACAGTAAC	SalI
pppE_Down_R	TTTGATGTTCCTCTTCGCTGG	
E_TP1_F	TGCCGTAACTGTACATGAGTG	
E_TP1_R	TTGGTGATCAGGTGAACGTG	
E_TP2_F	TGACCCTCACCACAGTAAC	
E_TP2_R	TGAGTTTTCCGATGCCTTCC	
pppF gene in-frame deletion and verification		
pppF_Up_F	CGTCCATTCTTCCACTTTGCTTGGCATCACGTTCACCTG	
pppF_Up_R	*GC*TCTAGAAGCAAAGCCCGTCTCAAC	XbaI
pppF_Down_F	*CCG*CTCGAGAGCTGTAACACCTGGATCAAC	XhoI
pppF_Down_R	AGCAAAGTGGAAGAATGGACG	
F_TP1_F	AACTGGCTGGGAGTTCTTC	
F_TP1_R	AGCAAAGCCCGTCTCAAC	
F_TP2_F	AGCTGTAACACCTGGATCAAC	
F_TP2_R	ACGGCACTGTTCTGGTTG	
pppG gene in-frame deletion and verification		
pppG_Up_F	GGTATACACAGCATCGCAGAAAGCATCCGGAATTGACG	
pppG_Up_R	*GC*TCTAGACAGTACTCGTGCATTCTCG	XbaI
pppG_Down_F	*ACGC*GTCGACTGCTTCCAACTGACCAGC	SalI
pppG_Down_R	TCTGCGATGCTGTGTATACC	
TP_pppG_F	AGGATGGAAAGTACTCAACG	
TP_pppG_R	TTCAGATATGGATGCGTCG	
pppH gene in-frame deletion and verification		
oLI32	GTCTTCTTCAGCAGTTTTTTGTTGGTATGGGTTCATTTTACACC	
oLI33	*AACTG*TCTAGACAGTCATCATCGTGATGTC	XbaI; Pairs with oLI32
oLI34	*AACTG*TCTAGACATCTGAGCGGCTTCTAT	XbaI

oLI35	CAACAAAAAACTGCTGAAGAGA	Pairs with oLI34
pDS132_F	AGTGAACGGCAGGTATATGTG	
pDS132_R	TGTGGAATTGTGAGCGGATAAC	
JD_P2	CAACTTGCTCTATTCGGGTC	Pairs with oLI38 below
pppI gene in-frame deletion and verification		
oLI36	CTGACCAAACGCTACGTTCCCATGGGCATGAGAATCTCCAAGCA	
oLI37	*AACTG*TCTAGAGATCTCCAAAGATGCTACGATC	XbaI; Pairs with oLI36
oLI38	*AACTG*TCTAGACGTAGCTCTCCAAACTTTCG	XbaI
oLI39	CATGGGAACGTAGCGTTTG	Pairs with oLI38
oLI45	GCAGATTGCGAGTGTAAGGAAAG	Pairs with oLI33 (*pppI*) or oLI37 (*pppJ*)
pppJ gene in-frame deletion		
oLI40	AAGGCTGAGTAAAGCAACCTCGTCGGTTTCAAGCTGAGGTCG	
oLI41	*AACTG*TCTAGAGACAGCCTCTACTTACACCA	XbaI; Pairs with oLI40
oLI46	*AACTG*GAGCTCTGGTCATCAACCTAGAACTCC	SacI
oLI43	GACGAGGTTGCTTTACTCAG	Pairs with oLI46
pppK gene in-frame deletion and verification		
pppK_Up_F	GGATGCGGCAGTTCAGAGCAAGATCTACACCACAAATTTCGGC	
pppK_Up_R	*GC*TCTAGATCGTATGGGCTTGAAAACTCTGG	XbaI
pppK_Down_F	*ACGC*GTCGACAGCCAACAAAGGAAGCGTGG	SalI
pppK_Down_R	GCTCTGAACTGCCGCATCC	
TP_pppK_F	TGATCGTCTCTATGGGTATCAAAGC	
TP_pppK_R	GCTGTTTCACGACCTTATCTGCG	
pppL gene in-frame deletion and verification		
ORF1_Up_F_2	*ACGC*GTCGACCAACGGAACAGCTCAACG	SalI
ORF1_Up_R	AGAATAGCGGTCAATCAACG	
ORF1_Down_F_2	CGTTGATTGACCGCTATTCTGTGCTCCAGAAACTCGTC	
ORF1_Down_R	*GC*TCTAGACTATGAGATGTTATCCCCTGC	XbaI
TP_ORF1_F_2	GCTAGAAATCTCCGATGCC	
TP_ORF1_R	AAGTCTCGTTTTGTAGCCG	
pppM gene in-frame deletion and verification		
ORF2_Up_F	*ACGC*GTCGACAGGTGAAACTGGCAAACG	SalI
ORF2_Up_R	GTAGTAATCCTTCGCACCG	
ORF2_Down_F	CGGTGCGAAGGATTACTACTCAGACATCAGCCTTGCC	
ORF2_Down_R	*GC*TCTAGAGATGCCCAGTTTCAAAATGC	XbaI
TP_ORF2_F	GTTTCCATCGATTACACCG	
TP_ORF2_R	CAGGCAAAACAGGTGAGC	
pYD004 backbone (Gibson assembly)		
YD_4_F	GGTATCAGCTCACTCAAAGG	
YD_4_R	TCCTACATCGAAGCTGAAAG	
luxR gene in-frame deletion and verification (Gibson assembly)		
luxR_Up_F	TTCAGCCACCAACGGATTCATTTTATACACCC	
luxR_Up_R	TTTGAGTGAGCTGATACCGCTCTGGAATGAATAAAGACG	
luxR_Down_F	TTCAGCTTCGATGTAGGAACCGATCTCCAGAGCAAG	
luxR_Down_R	AATCCGTTGGTGGCTGAAGGTTTCCG	
luxR_SCO_F	TCAGCCACCAACGGATT	
luxR_DCO_F	TCTTTGATCACGCGCATAC	
luxR_DCO_R	GGCTGGGATACTGAAAATGC	
Native promoter of *pppA* gene amplification		
Pro_pppA_F	*GC*TCTAGAATGAACAAAACAACCTGAG	XbaI
Pro_pppA_R	*CCC*AAGCTTACAACTACCGATGATTGAG	HindIII

pppH trans-complementation		
pppH_comp_F	*GGAATTC*CATATGTCTCCAAGCAGCAAGAAC	NdeI
pppH_comp_R	*CG*GGATCCGAGGAGGTCGCATGATATC	BamHI
pppI trans-complementation		
pppI_comp_F	*GGAATTC*CATATGGTCATACATGCCGCTAAG	NdeI
pppI_comp_R	*GA*AGATCTAAACTGCTGAAGAAGACAC	BglII
pppJ trans-complementation		
pppJ_comp_F	*GGAATTC*CATATGATGCATGACGTTTCCTCC	NdeI
pppJ_comp_R	*GA*AGATCTCTCGATGCATTCACCAAC	BglII
pppK trans-complementation		
pppK_F	*GGAATTC*CATATGTCAACCTAGAACTCCCACC	NdeI
pppK_R	*CGG*GGATCCAGCAGCTCACTTGTACATCAG	BamHI
pAM4891 backbone (Gibson assembly)		
pAM4891_GA_F	AGAAGGCCATCCTGACGG	
pAM4891_GA_R	AGATCTGGGTACCATTATACGAG	
pppD trans-complementation (Gibson assembly)		
pppD_GA_F	ATAATGGTACCCAGATCTGCTTAGAGGTCATCATGAACAGC	
pppD_GA_R	CCGTCAGGATGGCCTTCTCTCACCCTTCCATTTTTATCAACTCG	
pppG trans-complementation (Gibson assembly)		
pppG_comp_F	ATAATGGTACCCAGATCTCTCAGGTTGTTTTGTTCATGTCTG	
pppG_comp_R	CCGTCAGGATGGCCTTCTTCACATGACGCGAACTAGATATC	
pppE trans-complementation (Gibson assembly)		
pppE_comp_F	ATAATGGTACCCAGATCTTGGCGTAAGACACAATGCCG	
pppE_comp_R	CCGTCAGGATGGCCTTCTGAGGGGTGAGGCGCTAGAT	
pppF trans-complementation (Gibson assembly)		
pppF_comp_F	ATAATGGTACCCAGATCTTTTTAAATGAGTTTTCCGATGCCT	
pppF_comp_R	CCGTCAGGATGGCCTTCTTGTGTCTTACGCCAGCTG	
luxR overexpression (restriction digestion; pJeM1 backbone)		
luxR_rha_F	CCCAAGCTTGGCAAATTTTACTCACTCGC	HindIII
luxR_rha_R	GGAATTCCATATGAAGGTTGTTGGAGGCATAATA	NdeI

*SOE-PCR or Gibson assembly overhangs are shown in blue, restriction sites in red and underlined. Random bases inserted to allow restriction of PCR fragments are in *italics*.

Table S9. Summary of RNA sample quality used in this study.

Sample	Nanodrop conc. (ng/µL)	260nm /280nm	260nm /230nm	Qubit conc. (ng/µL)	dsDNA conc. (ng/µL)	%dsDNA	RIN
**24 h samples**							
W1	54.8	2.15	2.28	176	3.48	1.98%	8.3
W2	50.9	2.06	2.44	159	3.04	1.91%	8.5
W3	48.8	2.07	2.35	154	2.26	1.47%	8.1
A1	68.7	2.35	3.45	228	6.34	2.78%	8.4
A2	47.2	2.33	1.22	180	3.2	1.78%	8.3
A3	43.2	2.53	0.4	190	3.14	1.65%	8.6
D1	68.3	2.29	3.09	226	1.21	0.54%	8.5
D2	29.8	2.6	2.67	126	1.41	1.12%	8.6
D3	46.5	2.43	4.01	173	3.2	1.85%	8.6
E1	40.7	2.54	5.41	168	2.12	1.26%	8.6
E2	37.5	2.46	0.93	149	1.92	1.29%	8.8
E3	78.4	2.28	3.28	268	7.22	2.69%	8.8
**48 h samples (Nanodrop readings only)**							
W1	42.2	2.23	2.14	N/A			
W2	17.1	2.38	0.17				
W3	9.6	1.99	1.39				
A1	35.2	2.20	2.20				
D1	22.1	2.14	1.84				
E1	14.0	2.23	1.15				

dsDNA: double-stranded DNA; RIN: RNA integrity number.

Figures

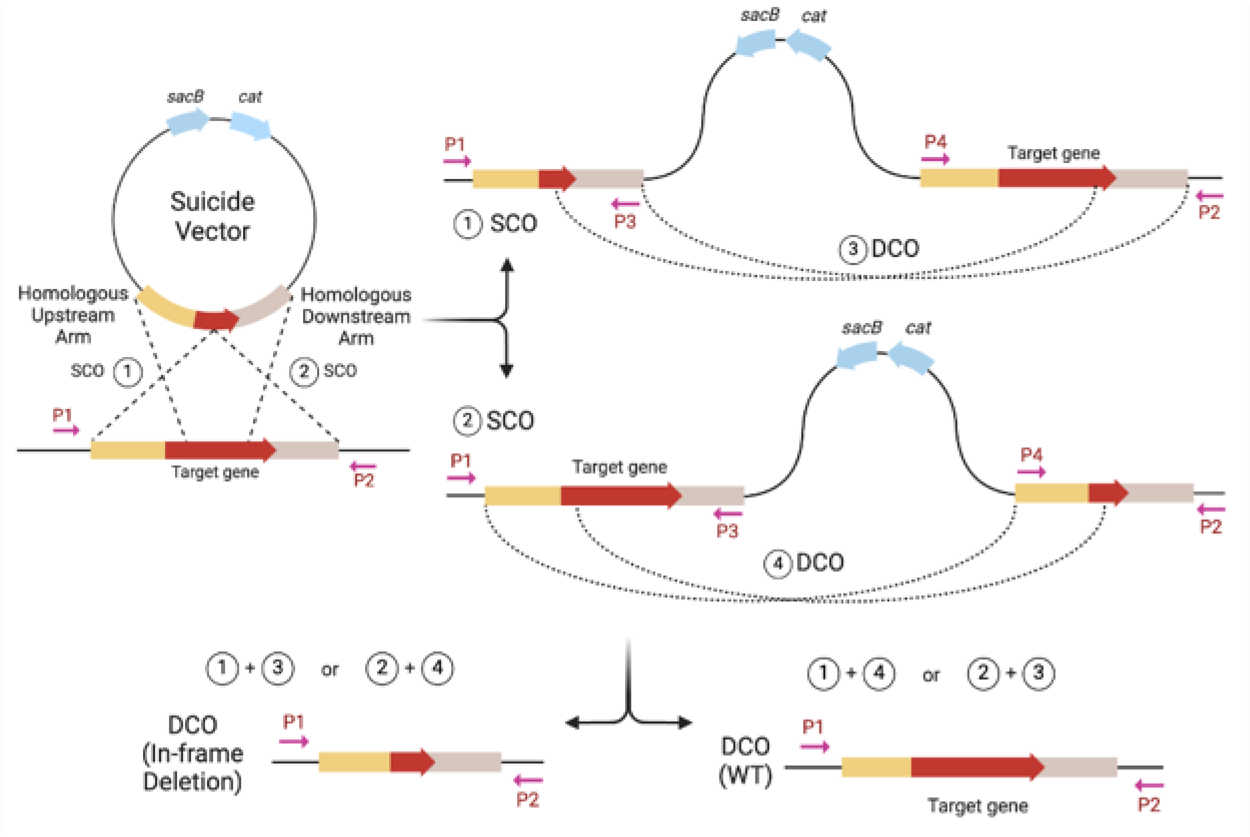

Figure S1. Scheme of in-frame deletion via homologous recombination. The first crossover event can happen at each of the two homologous sequences as indicated (single crossover, SCO 1 and 2). SCO mutants are obtained by selection on chloramphenicol and confirmed by PCR (not shown). The obtained SCO mutants are then grown on agar media without antibiotics but containing sucrose for counterselection of the vector backbone. The second crossover event (double crossover, DCO) can again happen at either arm resulting either in the desired in-frame deletion or in reversion to the wild type as indicated. P1, P2, P3, and P4 are primers used to verify SCO and DCO mutants by PCR. *sacB*, gene encoding levansucrase; *cat*, chloramphenicol acetyltransferase resistance gene.

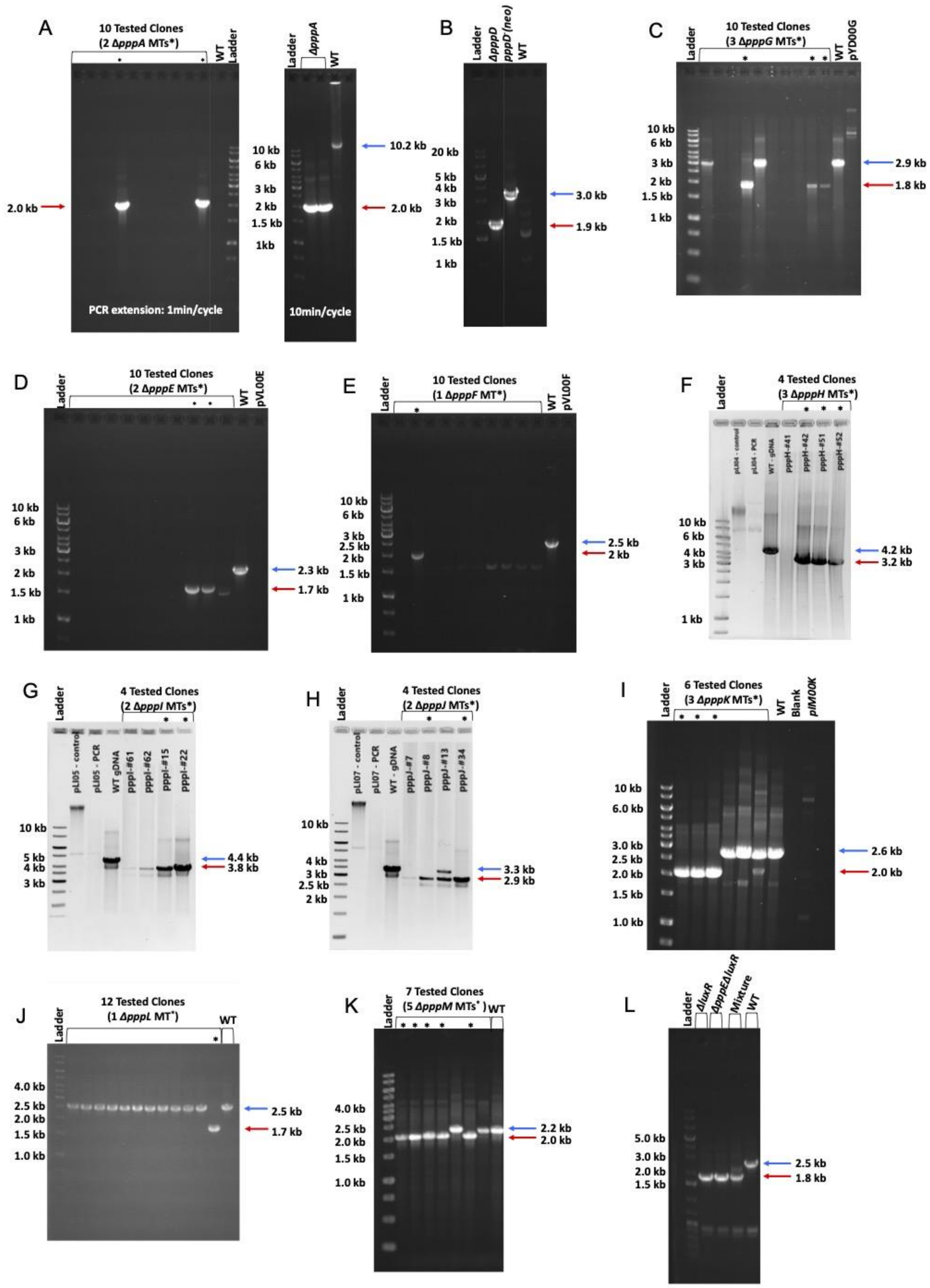

A
10 Tested Clones
(2 ΔpppA MTs*)
WT
Ladder
2.0 kb
PCR extension: 1min/cycle
Ladder
ΔpppA
WT
10 kb
6 kb
3 kb
2 kb
1.5 kb
1kb
10.2 kb
2.0 kb
10min/cycle
B
Ladder
ΔpppD
pppD⁻(neo)
WT
20 kb
5 kb
4 kb
3 kb
2 kb
1.5 kb
1 kb
3.0 kb
1.9 kb
C
Ladder
10 Tested Clones
(3 ΔpppG MTs*)
WT
pYD00G
10 kb
6 kb
3 kb
2 kb
1.5 kb
1 kb
2.9 kb
1.8 kb
D
Ladder
10 Tested Clones
(2 ΔpppE MTs*)
WT
pVL00E
10 kb
6 kb
3 kb
2 kb
1.5 kb
1 kb
2.3 kb
1.7 kb
E
Ladder
10 Tested Clones
(1 ΔpppF MT*)
WT
pVL00F
10 kb
6 kb
3 kb
2.5 kb
2 kb
1.5 kb
1 kb
2.5 kb
2 kb
F
Ladder
4 Tested Clones
(3 ΔpppH MTs*)
pppH-#41
pppH-#42
pppH-#51
pppH-#52
10 kb
6 kb
4 kb
3 kb
1 kb
4.2 kb
3.2 kb
G
Ladder
4 Tested Clones
(2 ΔpppI MTs*)
pLI05 - control
pLI05 - PCR
WT gDNA
pppI-#61
pppI-#62
pppI-#15
pppI-#22
10 kb
5 kb
4 kb
3 kb
4.4 kb
3.8 kb
H
Ladder
4 Tested Clones
(2 ΔpppJ MTs*)
pLI07 - control
pLI07 - PCR
WT - gDNA
pppJ-#7
pppJ-#8
pppJ-#13
pppJ-#34
10 kb
4 kb
3 kb
2.5 kb
2 kb
3.3 kb
2.9 kb
I
Ladder
6 Tested Clones
(3 ΔpppK MTs*)
WT
Blank
pIM00K
10 kb
6.0 kb
3.0 kb
2.5 kb
2.0 kb
1.5 kb
1.0 kb
2.6 kb
2.0 kb
J
Ladder
12 Tested Clones
(1 ΔpppL MT*)
WT
4.0 kb
2.5 kb
2.0 kb
1.5 kb
1.0 kb
2.5 kb
1.7 kb
K
Ladder
7 Tested Clones
(5 ΔpppM MTs*)
WT
4.0 kb
2.5 kb
2.0 kb
1.5 kb
1.0 kb
2.2 kb
2.0 kb
L
Ladder
ΔluxR
ΔpppEΔluxR
Mixture
WT
5.0 kb
3.0 kb
2.0 kb
1.5 kb
2.5 kb
1.8 kb

Figure S2. Gel electrophoresis images of scarless in-frame deletion mutants verified by PCR. Comparison of PCR amplification products between in-frame deletion mutants (MTs) and the wild type (WT) (**A**) Δ*pppA*. Here two PCRs were performed, one with a shorter extension (left) and another with a longer extension time required for amplifying the WT *pppA* (right). (**B**) Δ*pppD* . The Δ*pppD* mutant was generated from the *pppD::neo* mutant as parent. No band would show up for the wild type due to the short extension time used. (**C**) Δ*pppG*. (**D**) Δ*pppE*. (**E**) Δ*pppF*. (**F**) Δ*pppH*. (**G**) Δ*pppI*. (**H**) Δ*pppJ*. (**I**) Δ*pppK*. (**J**) Δ*pppL*. (**K**) Δ*pppM*. (**L**) Δ*pppE* Δ*luxR* and WT Δ*luxR*. Mixture means a mixture of the wild type and mutants. An asterisk (*) indicates positive in-frame deletion clones. The blue arrows indicate the size of wild type bands, and the red arrows the size of expected mutant bands.

748

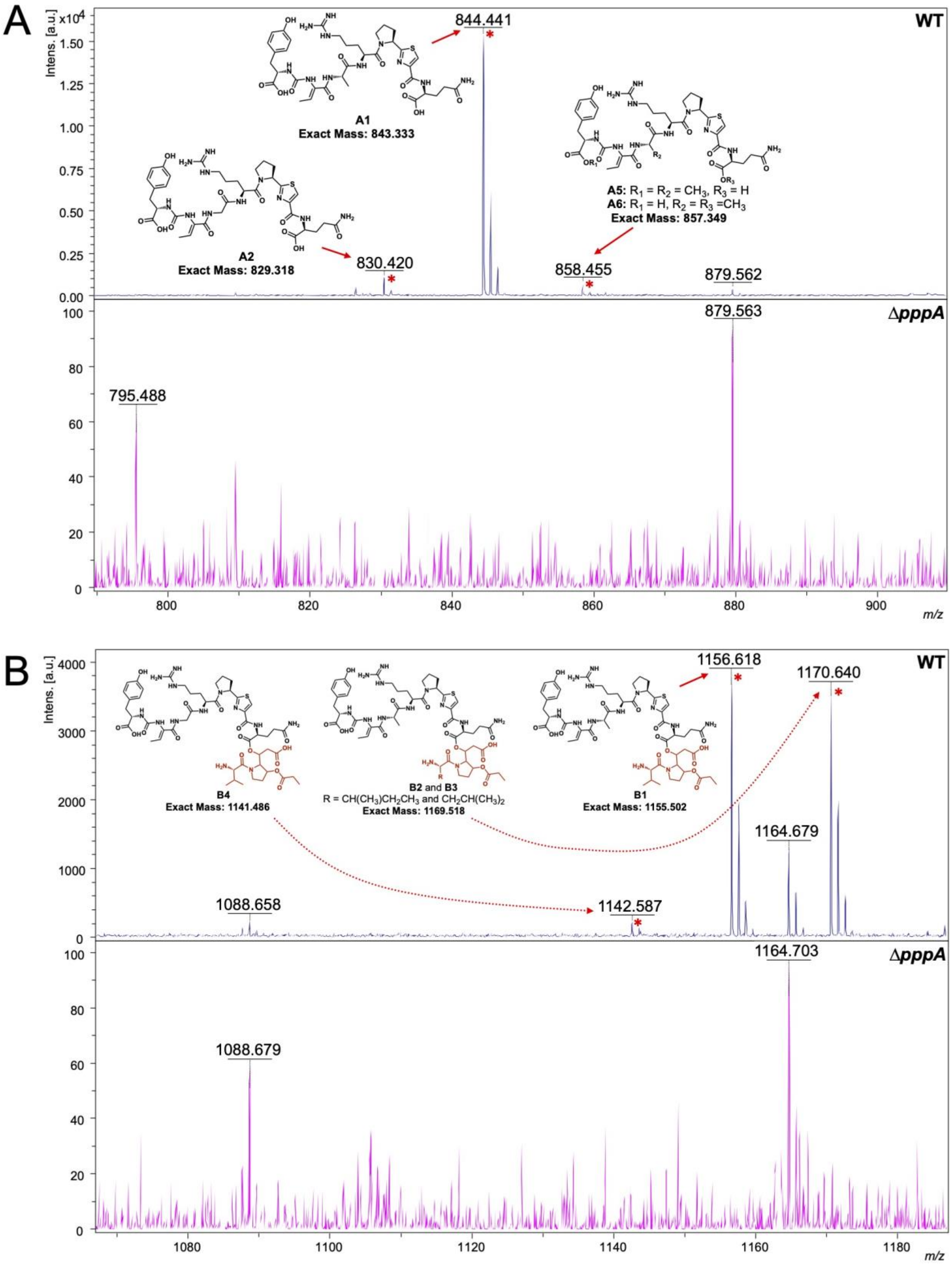
A
x10^4
Intens. [a.u.]
WT
844.441
A1
Exact Mass: 843.333
A2
Exact Mass: 829.318
830.420
A5: R1 = R2 = CH3, R3 = H
A6: R1 = H, R2 = R3 =CH3
Exact Mass: 857.349
858.455
879.562
ΔpppA
879.563
795.488
m/z
B
WT
1156.618
1170.640
B4
Exact Mass: 1141.486
B2 and B3
R = CH(CH3)CH2CH3 and CH2CH(CH3)2
Exact Mass: 1169.518
B1
Exact Mass: 1155.502
1164.679
1088.658
1142.587
ΔpppA
1164.703
1088.679
m/z

Figure S3. MALDI-ToF MS analysis of wild type and Δ*pppA* mutants. (**A**) The spectrum is zoomed in the range from *m/z* 790 to *m/z* 910. Molecular features of four PA congeners are indicated with red asterisks, that is, *m/z* 830.4 (PA3 $[M+H]^+$), *m/z* 844.4 (PA1 $[M+H]^+$), and *m/z* 858.4 (PA5 or PA6 $[M+H]^+$). (**B**) The spectrum is zoomed in the range from *m/z* 1068 to *m/z* 1183. Molecular features of four PB congeners are indicated with red asterisks, that is, *m/z* 1142.6 (PB4 Val, $[M+H]^+$), *m/z* 1156.6 (PB1 Val, $[M+H]^+$), *m/z* 1170.6 (PB2 or PB3 Ile or Leu, $[M+H]^+$).

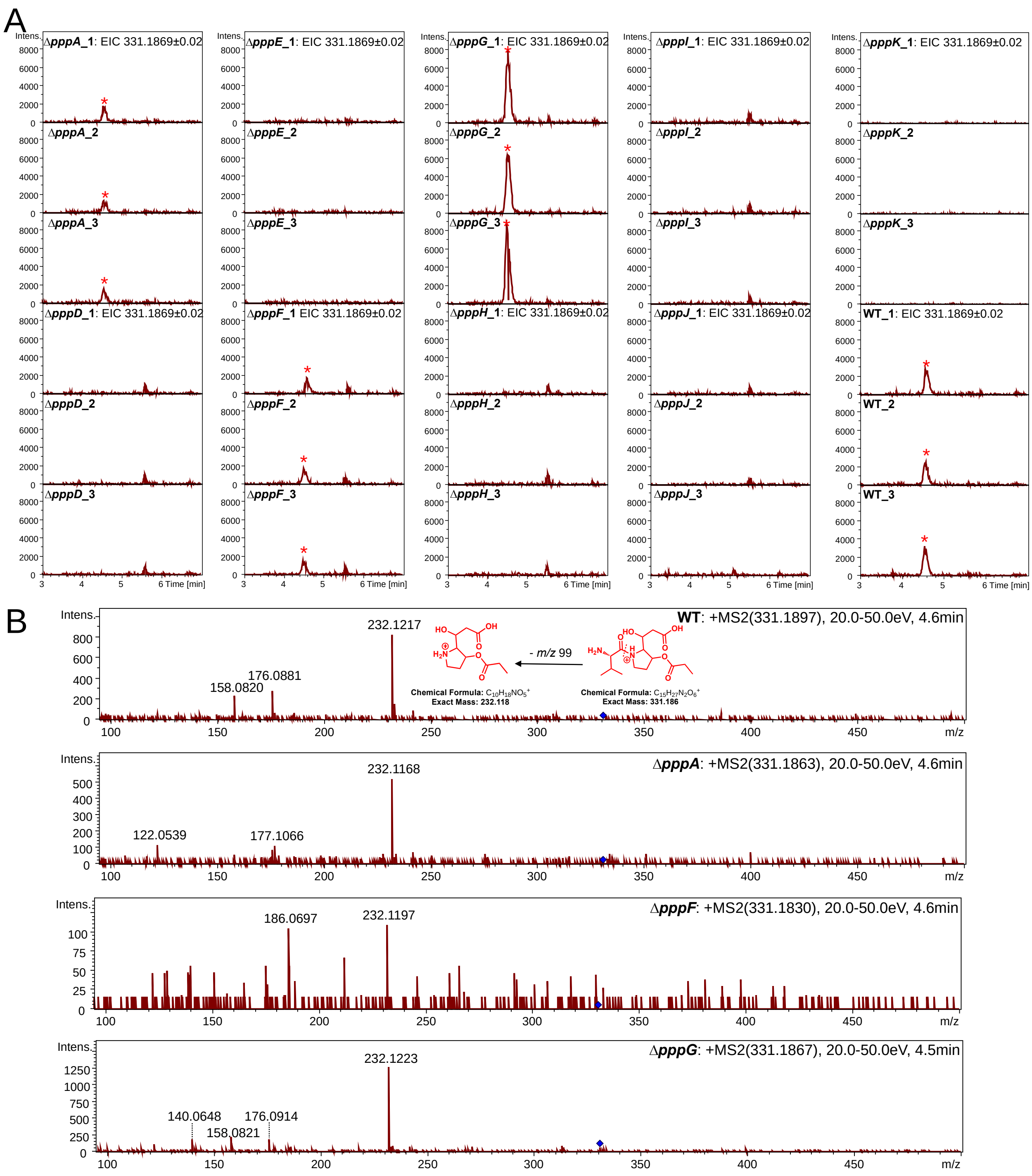

Figure S4. Comparison of PC1 production and MS/MS fragmentation between wild type and mutants in triplicates. UPLC-QToF-MS analyses were performed for both WT and mutants in triplicates. (**A**) Extracted Ion Chromatograms (EIC) of singly charged PC1 (Val, $[M+H]^+$). The same mass filter (the expected *m/z* ratio ± *m/z* 0.02) was applied to all samples. PC1 peaks are

highlighted with a red asterisk. (**B**) Fragmentation pattern of PC1 produced from WT compared to Δ*pppA/F/G* mutants*.* The proposed structures of parent and fragment peaks are shown. The upper right numbers from left to right are the *m/z* of the fragmentated compound, the collision energy, and the retention time. The blue diamond pinpoints the parent peak.

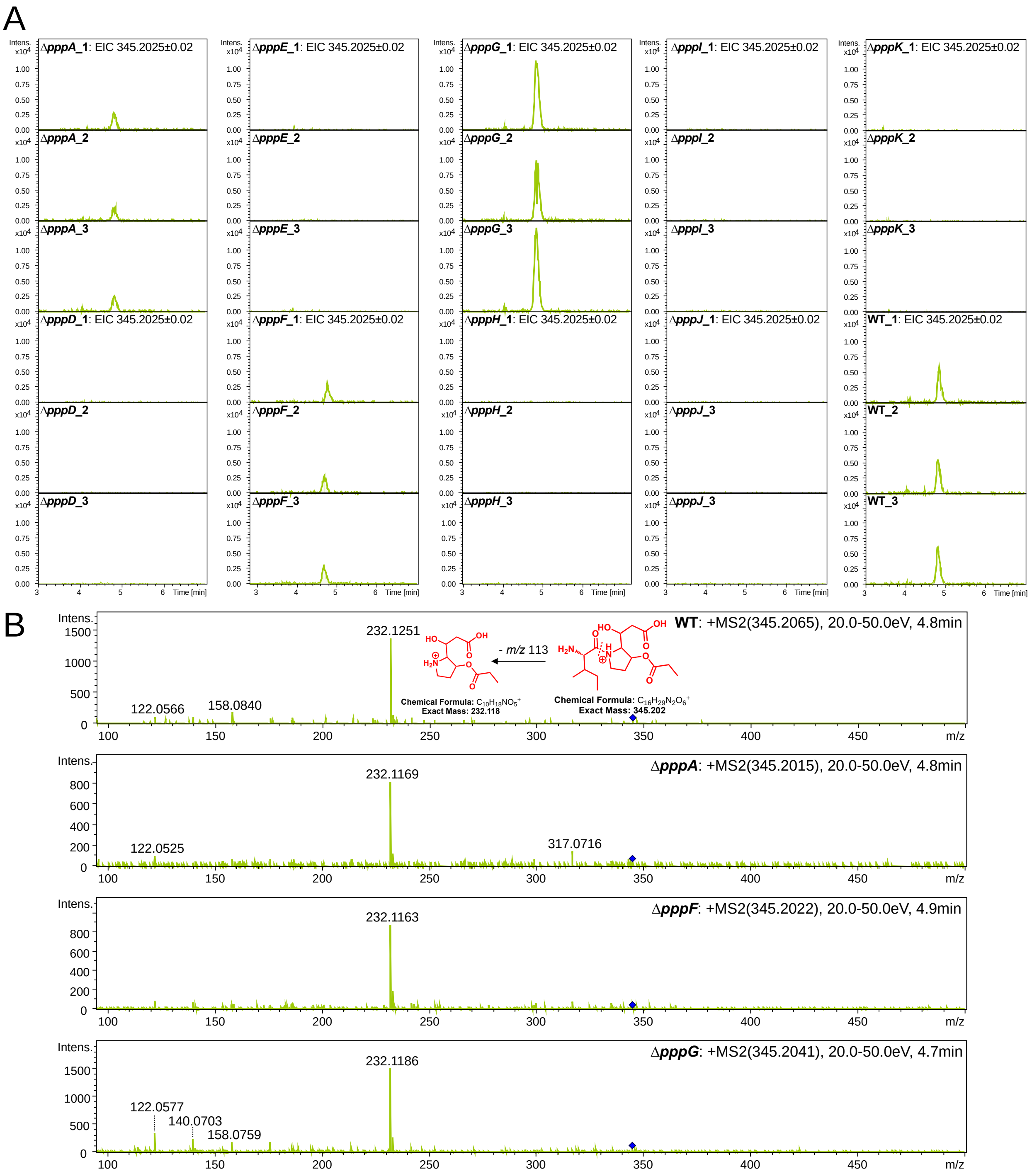

Figure S5. Comparison of PC2 or PC3 production and MS/MS fragmentation between wild type and mutants in triplicates. UPLC-QToF-MS analyses were performed for both WT and mutants in triplicates. (**A**) Extracted Ion Chromatograms (EIC) of singly charged PC2 or PC3 (Ile or Leu, $[M+H]^+$). The same mass filter (the expected *m/z* ± 0.02) was applied to all samples. (**B**) The

fragmentation pattern of PC2 or PC3 produced from WT compared to Δ*pppA/F/G* mutants. The proposed structures of parent and fragment peaks are shown. The blue diamond pinpoints the parent peak.

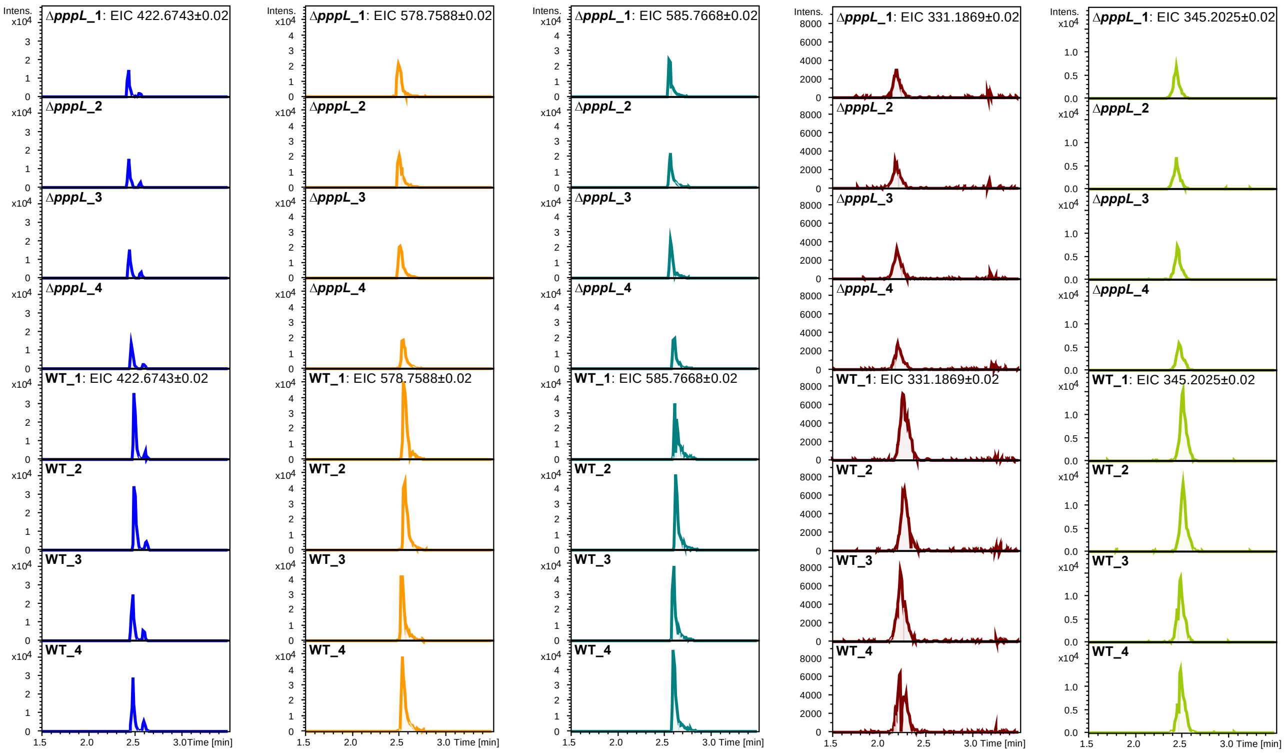

Figure S6. Comparison of PA, PB, and PC production between wild type and Δ*pppL* mutants. UPLC-QToF-MS analyses were performed for both WT and Δ*pppL* in quadruplicates. Extracted Ion Chromatograms (EIC) of (from left to right) doubly charged PA1 (*m/z* 442.6743, $[M+2H]^{2+}$), doubly charged PB1 (*m/z* 578.7588, Val, $[M+2H]^{2+}$) and PB2 or PB3 (*m/z* 585.7668, Ile or Leu, $[M+2H]^{2+}$), and singly charged PC1 (*m/z* 331.1869, Val, $[M+H]^{+}$) and PC2 or PC3 (*m/z* 345.2025, Ile or Leu, $[M+H]^{+}$). The same mass filter (the expected *m/z* ± 0.02) was applied to all samples.

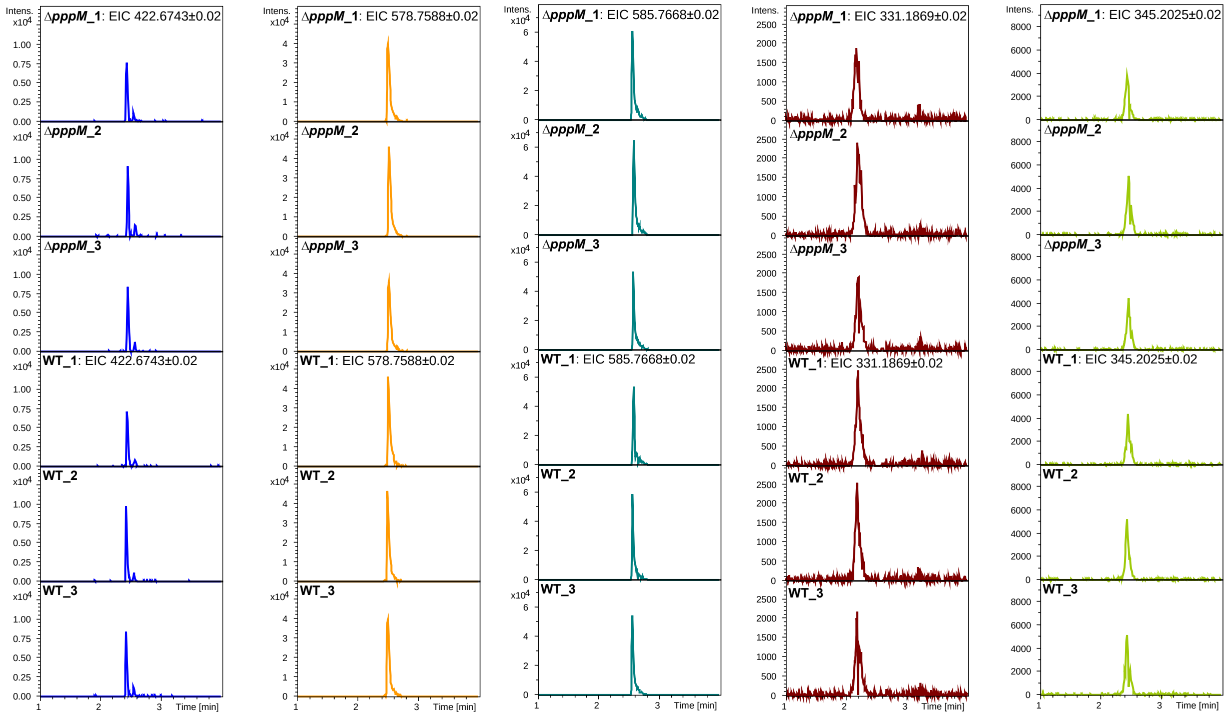

Figure S7. Comparison of PA, PB, and PC production between wild type and Δ*pppM* mutants. UPLC-QToF-MS analyses were performed for both WT and Δ*pppM* in triplicates. Extracted Ion Chromatograms (EIC) of (from left to right) doubly charged PA1 (*m/z* 442.6743, $[M+2H]^{2+}$), doubly charged PB1 (*m/z* 578.7588, Val, $[M+2H]^{2+}$) and PB2 or PB3 (*m/z* 585.7668, Ile or Leu, $[M+2H]^{2+}$), and singly charged PC1 (*m/z* 331.1869, Val, $[M+H]^{+}$) and PC2 or PC3 (*m/z* 345.2025, Ile or Leu, $[M+H]^{+}$). The same mass filter (the expected *m/z* ± 0.02) was applied to all samples.

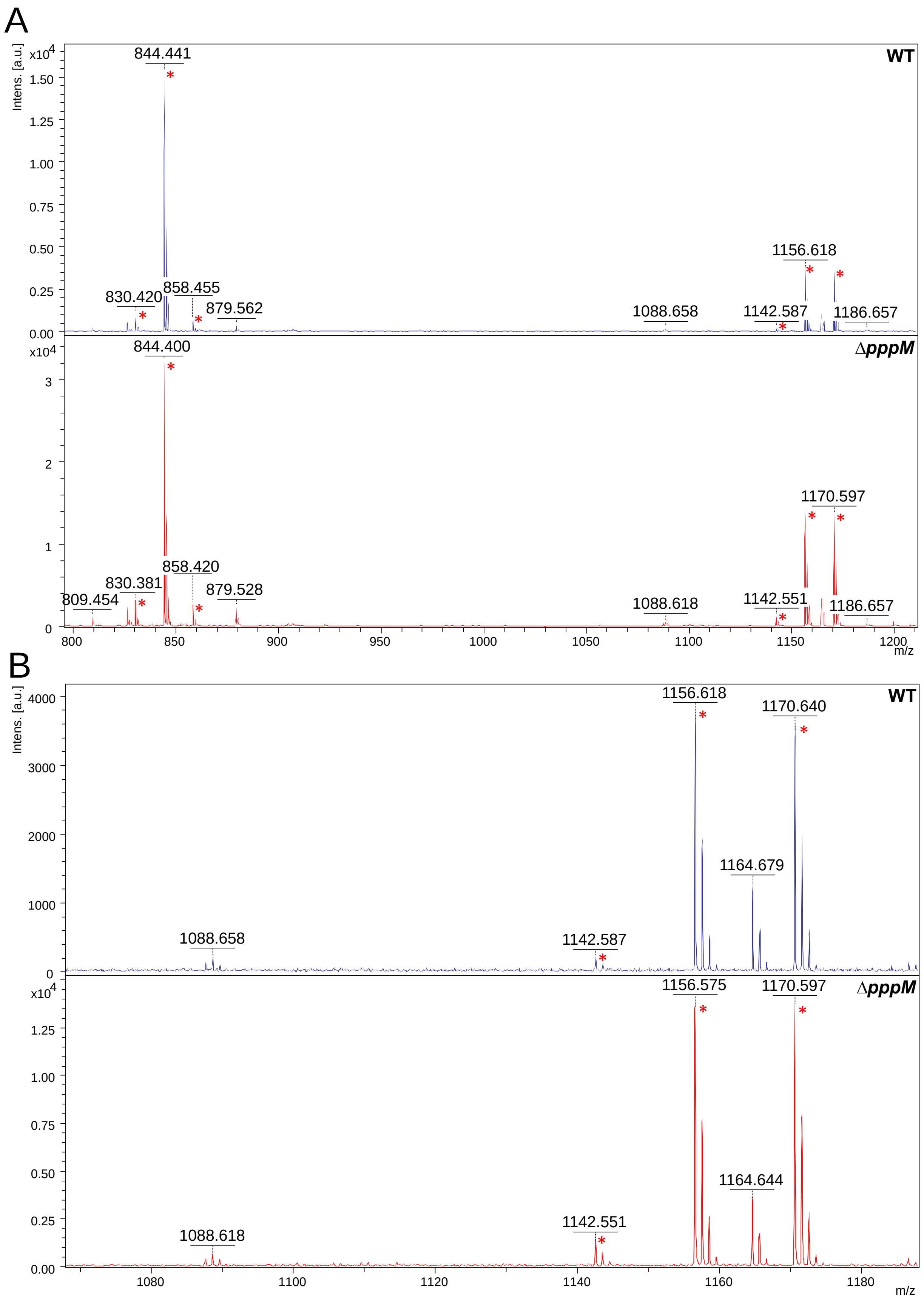
A
WT
ΔpppM
Intens. [a.u.]
x10^4
844.441
830.420
858.455
879.562
1088.658
1142.587
1156.618
1186.657
844.400
809.454
830.381
858.420
879.528
1088.618
1142.551
1170.597
1186.657
m/z
B
1156.618
1170.640
1164.679
1142.587
1088.658
1156.575
1170.597
1164.644
1142.551
1088.618

790

Figure S8. MALDI-ToF MS analyses of wild type and ∆*pppM* mutant. MALDI-ToF MS analyses were performed for both WT and ∆*pppM* mutant. (**A**) The molecular features for both PA and PB are indicated with red asterisks. *m/z* 830.4, PA3 ($[M+H]^+$); *m/z* 844.4, PA1 ($[M+H]^+$); and *m/z* 858.4, PA5 or PA6 ($[M+H]^+$). *m/z* 1142.6, PB4 (Val, $[M+H]^+$); *m/z* 1156.6, PB1 (Val, $[M+H]^+$); *m/z* 1170.6, PB2 or PB3 (Ile or Leu, $[M+H]^+$). (**B**) The spectrum is zoomed in the range from *m/z* 1068 to *m/z* 1183 to highlight that no modified PB was observed in the ∆*pppM* mutant.

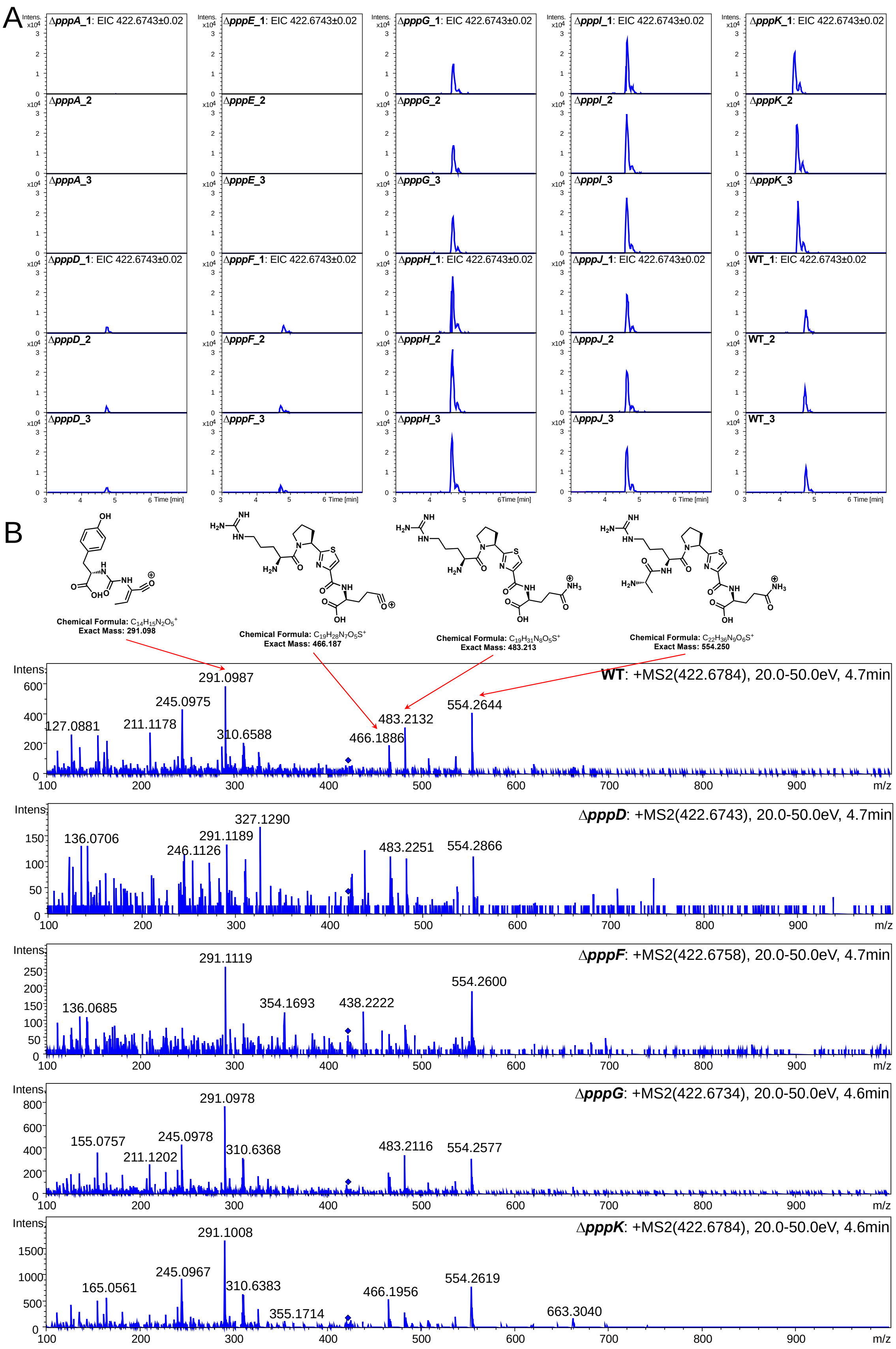

A
ΔpppA_1: EIC 422.6743±0.02
ΔpppA_2
ΔpppA_3
ΔpppE_1: EIC 422.6743±0.02
ΔpppE_2
ΔpppE_3
ΔpppG_1: EIC 422.6743±0.02
ΔpppG_2
ΔpppG_3
ΔpppI_1: EIC 422.6743±0.02
ΔpppI_2
ΔpppI_3
ΔpppK_1: EIC 422.6743±0.02
ΔpppK_2
ΔpppK_3
ΔpppD_1: EIC 422.6743±0.02
ΔpppD_2
ΔpppD_3
ΔpppF_1: EIC 422.6743±0.02
ΔpppF_2
ΔpppF_3
ΔpppH_1: EIC 422.6743±0.02
ΔpppH_2
ΔpppH_3
ΔpppJ_1: EIC 422.6743±0.02
ΔpppJ_2
ΔpppJ_3
WT_1: EIC 422.6743±0.02
WT_2
WT_3
Intens. x10^4
Time [min]
B
Chemical Formula: C14H15N2O5+
Exact Mass: 291.098
Chemical Formula: C19H28N7O5S+
Exact Mass: 466.187
Chemical Formula: C19H31N8O5S+
Exact Mass: 483.213
Chemical Formula: C22H36N9O6S+
Exact Mass: 554.250
WT: +MS2(422.6784), 20.0-50.0eV, 4.7min
127.0881
211.1178
245.0975
291.0987
310.6588
466.1886
483.2132
554.2644
ΔpppD: +MS2(422.6743), 20.0-50.0eV, 4.7min
136.0706
246.1126
291.1189
327.1290
483.2251
554.2866
ΔpppF: +MS2(422.6758), 20.0-50.0eV, 4.7min
136.0685
291.1119
354.1693
438.2222
554.2600
ΔpppG: +MS2(422.6734), 20.0-50.0eV, 4.6min
155.0757
211.1202
245.0978
291.0978
310.6368
483.2116
554.2577
ΔpppK: +MS2(422.6784), 20.0-50.0eV, 4.6min
165.0561
245.0967
291.1008
310.6383
355.1714
466.1956
554.2619
663.3040
Intens.
m/z

Figure S9. Comparison of PA1 production and MS/MS fragmentation between wild type and mutants in triplicates. UPLC-QToF-MS analyses were performed for both WT and mutants in triplicates. (**A**) Extracted Ion Chromatograms (EIC) of doubly charged PA1 ($[M+2H]^{2+}$). The same mass filter (the expected *m/z* ± 0.02) was applied to all samples. (**B**) The fragmentation pattern of PA1 produced from WT is compared to those of ∆*pppD/F/G/K* mutants. Proposed structures of major peaks are shown. The upper right numbers from left to right are the *m/z* of the fragmentated compound, the collision energy, and the retention time. The blue diamond pinpoints the parent peak.

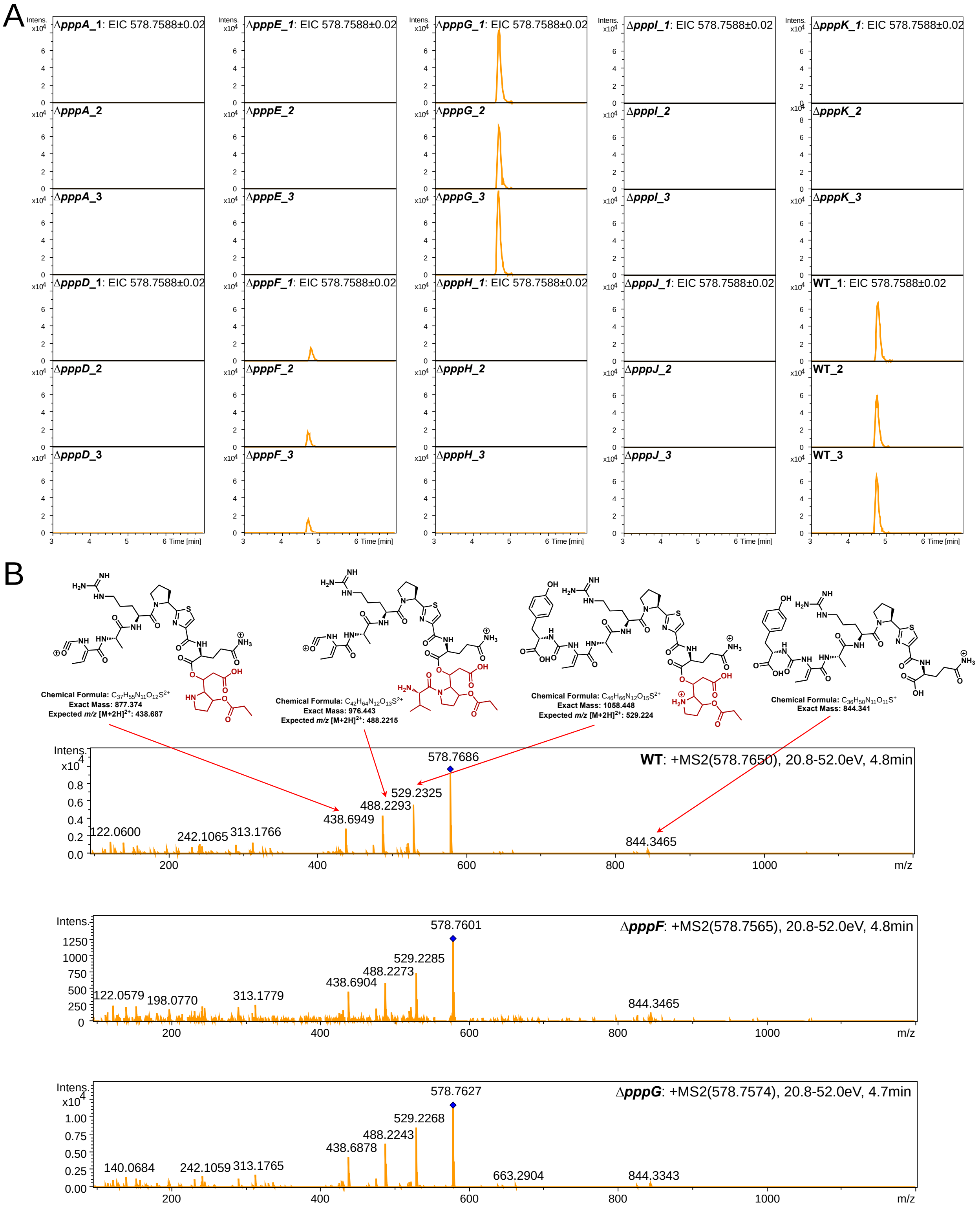
A
ΔpppA_1: EIC 578.7588±0.02
ΔpppA_2
ΔpppA_3
ΔpppD_1: EIC 578.7588±0.02
ΔpppD_2
ΔpppD_3
ΔpppE_1: EIC 578.7588±0.02
ΔpppE_2
ΔpppE_3
ΔpppF_1: EIC 578.7588±0.02
ΔpppF_2
ΔpppF_3
ΔpppG_1: EIC 578.7588±0.02
ΔpppG_2
ΔpppG_3
ΔpppH_1: EIC 578.7588±0.02
ΔpppH_2
ΔpppH_3
ΔpppI_1: EIC 578.7588±0.02
ΔpppI_2
ΔpppI_3
ΔpppJ_1: EIC 578.7588±0.02
ΔpppJ_2
ΔpppJ_3
ΔpppK_1: EIC 578.7588±0.02
ΔpppK_2
ΔpppK_3
WT_1: EIC 578.7588±0.02
WT_2
WT_3
Intens. x10^4
Time [min]
B
Chemical Formula: C37H55N11O12S2+
Exact Mass: 877.374
Expected m/z [M+2H]2+: 438.687
Chemical Formula: C42H64N12O13S2+
Exact Mass: 976.443
Expected m/z [M+2H]2+: 488.2215
Chemical Formula: C46H66N12O15S2+
Exact Mass: 1058.448
Expected m/z [M+2H]2+: 529.224
Chemical Formula: C36H50N11O11S+
Exact Mass: 844.341
WT: +MS2(578.7650), 20.8-52.0eV, 4.8min
578.7686
529.2325
488.2293
438.6949
122.0600
242.1065
313.1766
844.3465
ΔpppF: +MS2(578.7565), 20.8-52.0eV, 4.8min
578.7601
529.2285
488.2273
438.6904
122.0579
198.0770
313.1779
844.3465
ΔpppG: +MS2(578.7574), 20.8-52.0eV, 4.7min
578.7627
529.2268
488.2243
438.6878
140.0684
242.1059
313.1765
663.2904
844.3343
Intens.
m/z

Figure S10. Comparison of PB1 production and MS/MS fragmentation between wild type and mutants in triplicates. UPLC-QToF-MS analyses were performed for both WT and mutants in triplicates. (**A**) Extracted Ion Chromatograms (EIC) of doubly charged PB1 (Val, $[M+2H]^{2+}$). The same mass filter (the expected *m/z* ± 0.02) was applied to all samples. (**B**) The fragmentation pattern of PB1 produced from WT compared to those of Δ*pppF/G* mutants. Proposed structures of major peaks are shown. The upper right numbers from left to right are the *m/z* of the fragmentated compound, the collision energy, and the retention time. The blue diamond pinpoints the parent peak.

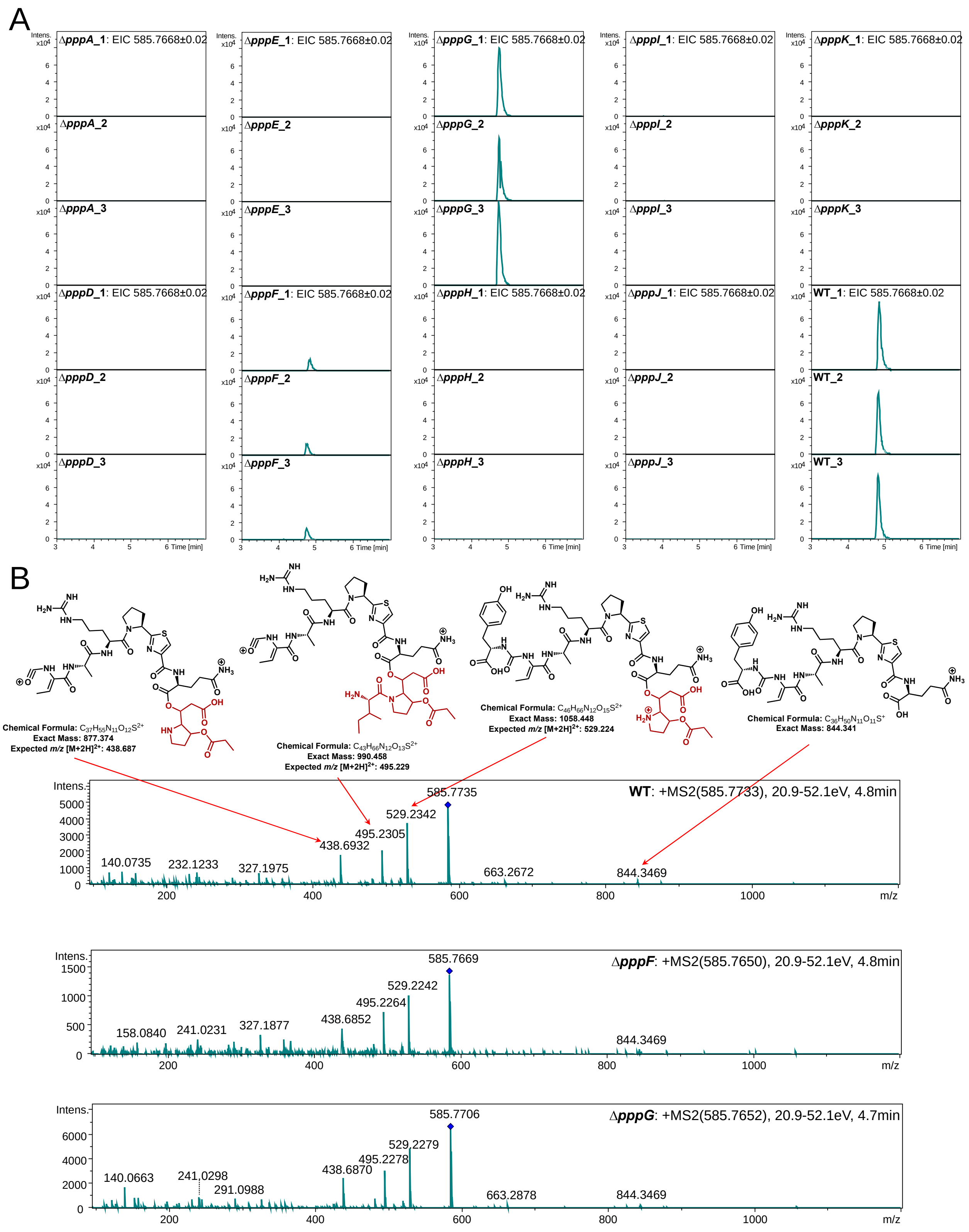
A
ΔpppA_1: EIC 585.7668±0.02
ΔpppA_2
ΔpppA_3
ΔpppD_1: EIC 585.7668±0.02
ΔpppD_2
ΔpppD_3
ΔpppE_1: EIC 585.7668±0.02
ΔpppE_2
ΔpppE_3
ΔpppF_1: EIC 585.7668±0.02
ΔpppF_2
ΔpppF_3
ΔpppG_1: EIC 585.7668±0.02
ΔpppG_2
ΔpppG_3
ΔpppH_1: EIC 585.7668±0.02
ΔpppH_2
ΔpppH_3
ΔpppI_1: EIC 585.7668±0.02
ΔpppI_2
ΔpppI_3
ΔpppJ_1: EIC 585.7668±0.02
ΔpppJ_2
ΔpppJ_3
ΔpppK_1: EIC 585.7668±0.02
ΔpppK_2
ΔpppK_3
WT_1: EIC 585.7668±0.02
WT_2
WT_3
Intens. x10^4
Time [min]
B
Chemical Formula: C37H55N11O12S2+
Exact Mass: 877.374
Expected m/z [M+2H]2+: 438.687
Chemical Formula: C43H66N12O13S2+
Exact Mass: 990.458
Expected m/z [M+2H]2+: 495.229
Chemical Formula: C46H66N12O15S2+
Exact Mass: 1058.448
Expected m/z [M+2H]2+: 529.224
Chemical Formula: C36H50N11O11S+
Exact Mass: 844.341
WT: +MS2(585.7733), 20.9-52.1eV, 4.8min
585.7735
529.2342
495.2305
438.6932
140.0735
232.1233
327.1975
663.2672
844.3469
ΔpppF: +MS2(585.7650), 20.9-52.1eV, 4.8min
585.7669
529.2242
495.2264
438.6852
158.0840
241.0231
327.1877
844.3469
ΔpppG: +MS2(585.7652), 20.9-52.1eV, 4.7min
585.7706
529.2279
495.2278
438.6870
140.0663
241.0298
291.0988
663.2878
844.3469
Intens.
m/z

Figure S11. Comparison of PB2 or PB3 production and MS/MS fragmentation between wild type and mutants in triplicates. UPLC-QToF-MS analyses were performed for both WT and mutants in triplicates. (**A**) Extracted Ion Chromatograms (EIC) of doubly charged PB2 or PB3 (Ile or Leu, $[M+2H]^{2+}$). The same mass filter (the expected *m/z* ± 0.02) was applied to all samples. (**B**) The fragmentation pattern of PB2 or PB3 produced from WT compared to Δ*pppF/G* mutants. Proposed structures of major peaks are shown. The structure of PB2 is shown as the representative of peak at *m/z* 495.23. The upper right numbers from left to right are the *m/z* of the fragmentated compound, the collision energy, and the retention time. The blue diamond pinpoints the parent peak.

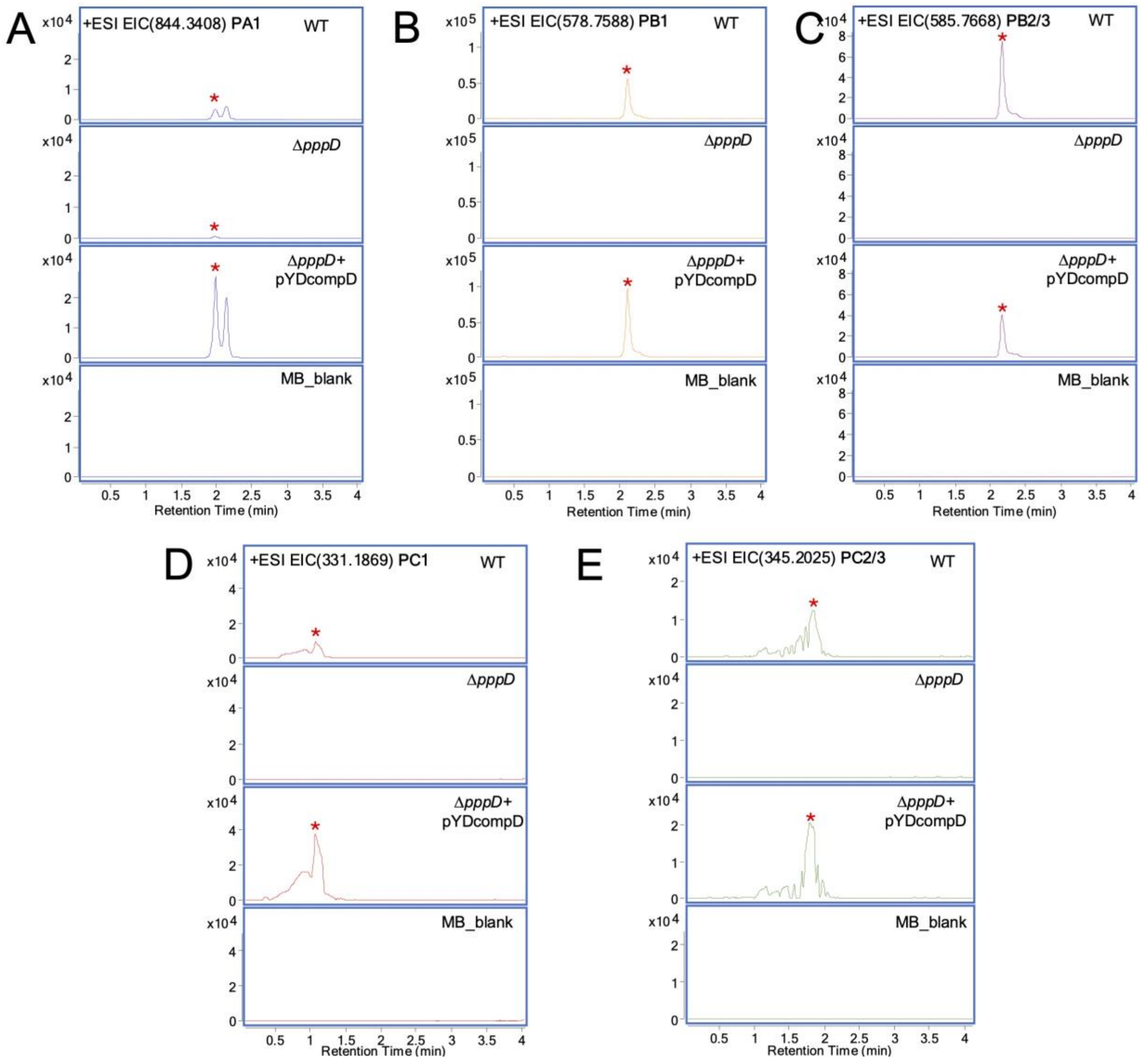

Figure S12. Comparison of PA, PB, and PC production between Δ*pppD* mutant, wild type and genetically complemented Δ*pppD* mutant. UPLC-QToF-MS analyses were performed for the Δ*pppD* mutant, the complemented Δ*pppD* mutant, the wild type, and the marine broth blank. Extracted Ion Chromatograms (EIC) of (**A**) singly charged PA1 (*m/z* 844.3408, [M+H]$^{1+}$), (**B**) doubly charged PB1 (*m/z* 578.7588, Val, [M+2H]$^{2+}$), (**C**) doubly charged PB2 or PB3 (*m/z* 585.7668, Ile or Leu, [M+2H]$^{2+}$), (**D**) singly charged PC1 (*m/z* 331.1869, Val, [M+H]$^{+}$), and (**E**) singly charged PC2 or PC3 (*m/z* 345.2025, Ile or Leu, [M+H]$^{+}$). The same mass filter (the expected *m/z* ± 10 ppm) was applied to all samples. Pseudovibriamides are indicated with red asterisks.

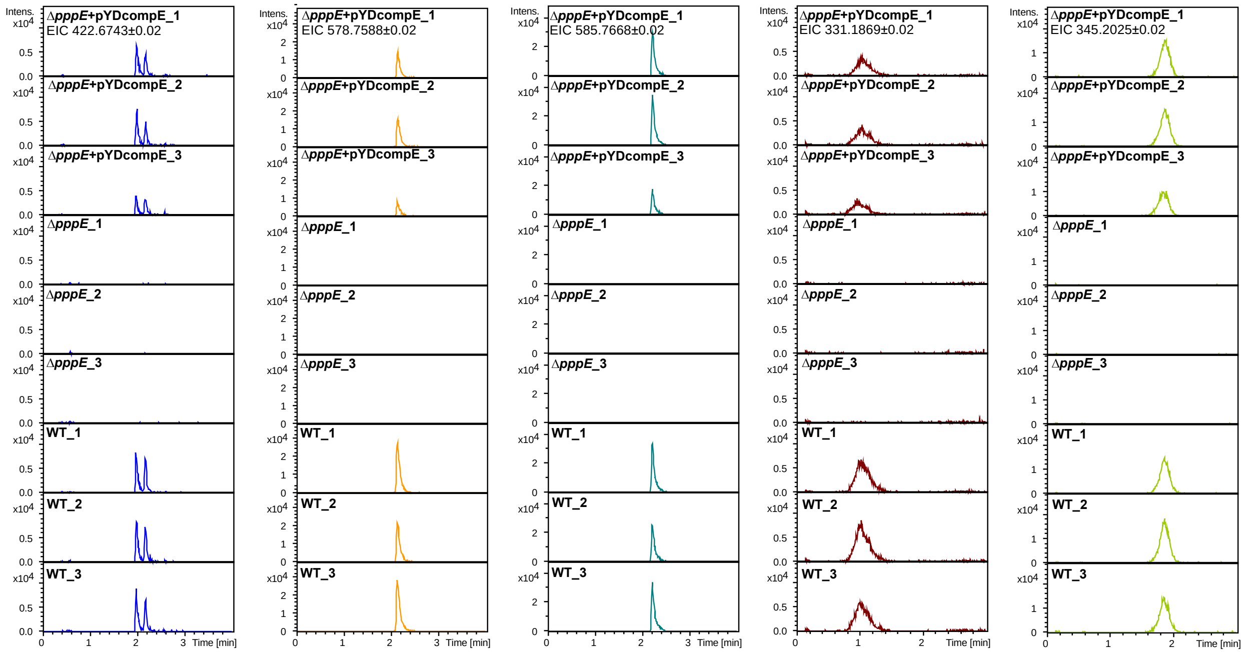

Figure S13. Comparison of PA, PB, and PC production between Δ*pppE* mutant, wild type and genetically complemented Δ*pppE* mutant. UPLC-QToF-MS analyses were performed for wild type (bottom), Δ*pppE* mutant (middle), and complemented Δ*pppE* mutant (top) in triplicates. Extracted Ion Chromatograms (EIC) of (from left to right) doubly charged PA1 (*m/z* 442.6743, $[M+2H]^{2+}$), doubly charged PB1 (*m/z* 578.7588, Val, $[M+2H]^{2+}$) and PB2 or PB3 (*m/z* 585.7668, Ile or Leu, $[M+2H]^{2+}$), and singly charged PC1 (*m/z* 331.1869, Val, $[M+H]^{+}$) and PC2 or PC3 (*m/z* 345.2025, Ile or Leu, $[M+H]^{+}$). The same mass filter (the expected *m/z* ± 0.02) was applied to all samples.

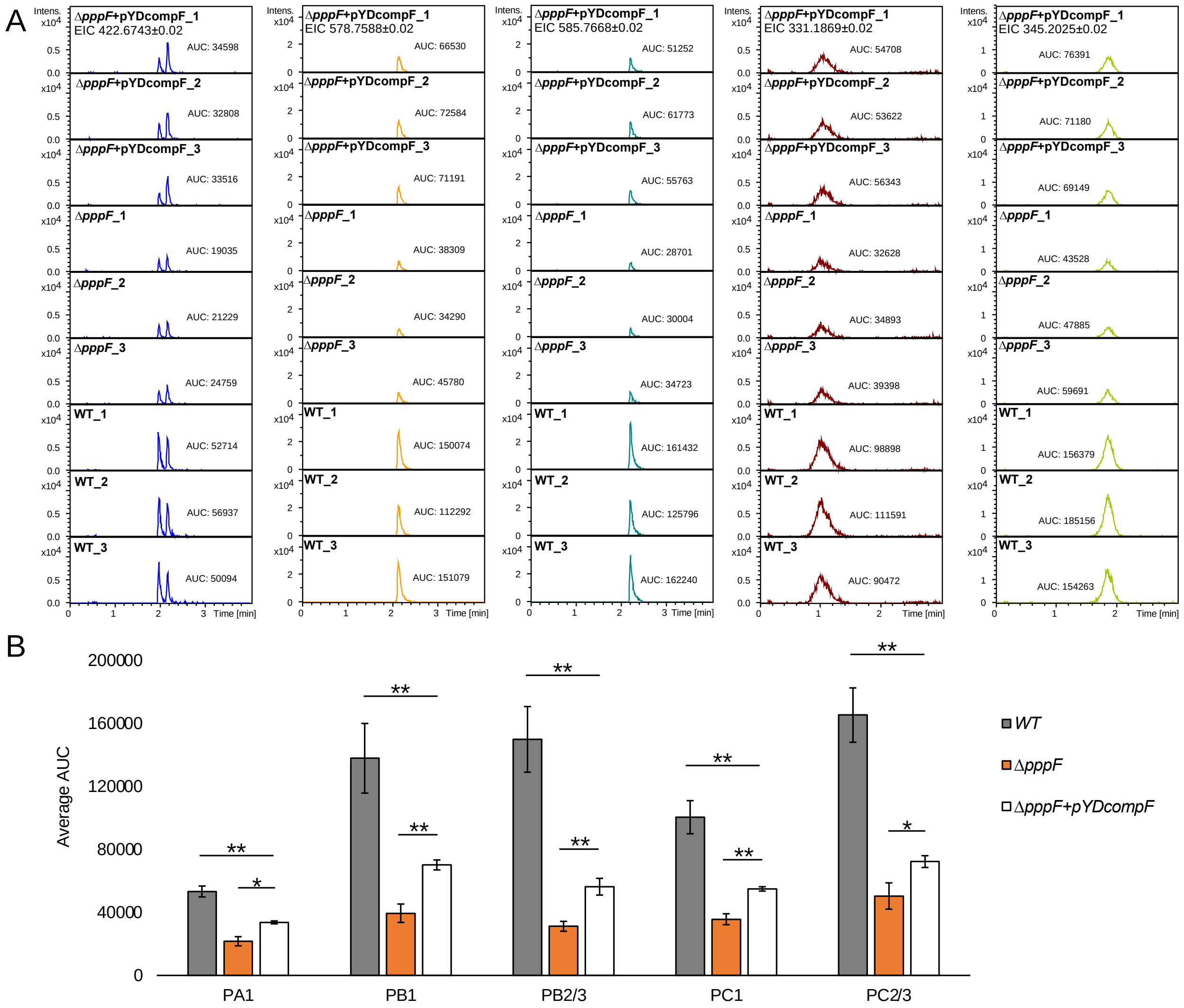

Figure S14. Comparison of PA, PB, and PC production between Δ*pppF* mutant, wild type and genetically complemented Δ*pppF* mutant. (**A**) UPLC-QToF-MS analyses were performed for the complemented Δ*pppF* mutant (top), wild type (bottom), and the Δ*pppF* mutant (middle) in triplicates. Extracted Ion Chromatograms (EIC) of (from left to right) doubly charged PA1 (*m/z* 442.6743, $[M+2H]^{2+}$), doubly charged PB1 (*m/z* 578.7588, Val, $[M+2H]^{2+}$) and PB2 or PB3 (*m/z* 585.7668, Ile or Leu, $[M+2H]^{2+}$), and singly charged PC1 (*m/z* 331.1869, Val, $[M+H]^{+}$) and PC2 or PC3 (*m/z* 345.2025, Ile or Leu, $[M+H]^{+}$). The same mass filter (the expected *m/z* ± 0.02) was applied to all samples. AUC, area under curve. (**B**) The average AUC of each pseudovibriamide from the wild type, the Δ*pppF* mutant, and the genetically complemented Δ*pppF* mutant was used

to assess the relative amount of each pseudovibriamide produced. Two-tail *P*-values from t-Test were used to determine statistical significance; *, *P*-value ≤ 0.05; and **, *P*-value ≤ 0.01.

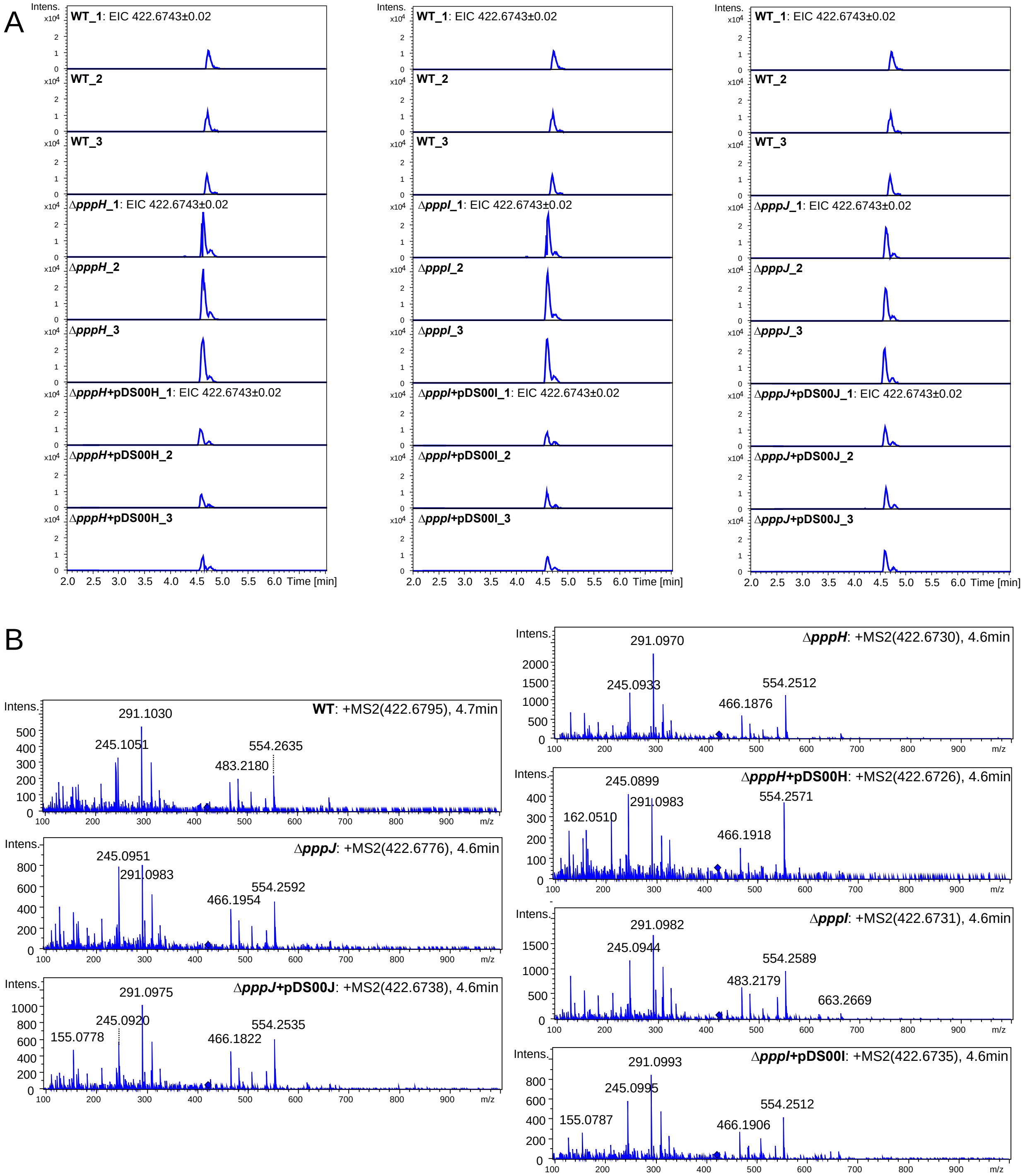

Figure S15. Comparison of PA1 production and MS/MS fragmentation between wild type, Δ*pppH*, Δ*pppI*, Δ*pppJ* mutants, and genetically complemented strains. UPLC-QToF-MS

analyses were performed in triplicates. (**A**) Extracted Ion Chromatograms (EIC) of doubly charged PA1 ($[M+2H]^{2+}$). The same mass filter (the expected *m/z* ± 0.02) was applied to all samples. (**B**) Fragmentation pattern of PA1 produced from WT compared to Δ*pppH,* Δ*pppI,* and Δ*pppJ* mutants and complemented Δ*pppH,* Δ*pppI,* and Δ*pppJ* mutants. Expected fragmentation peaks of PA1 are *m/z* 554.25, *m/z* 483.21, *m/z* 466.19, and *m/z* 291.10 (**Fig. S9**). The upper right numbers from left to right are the *m/z* ratio of the fragmentated compound, and the retention time. The blue diamond pinpoints the parent peak.

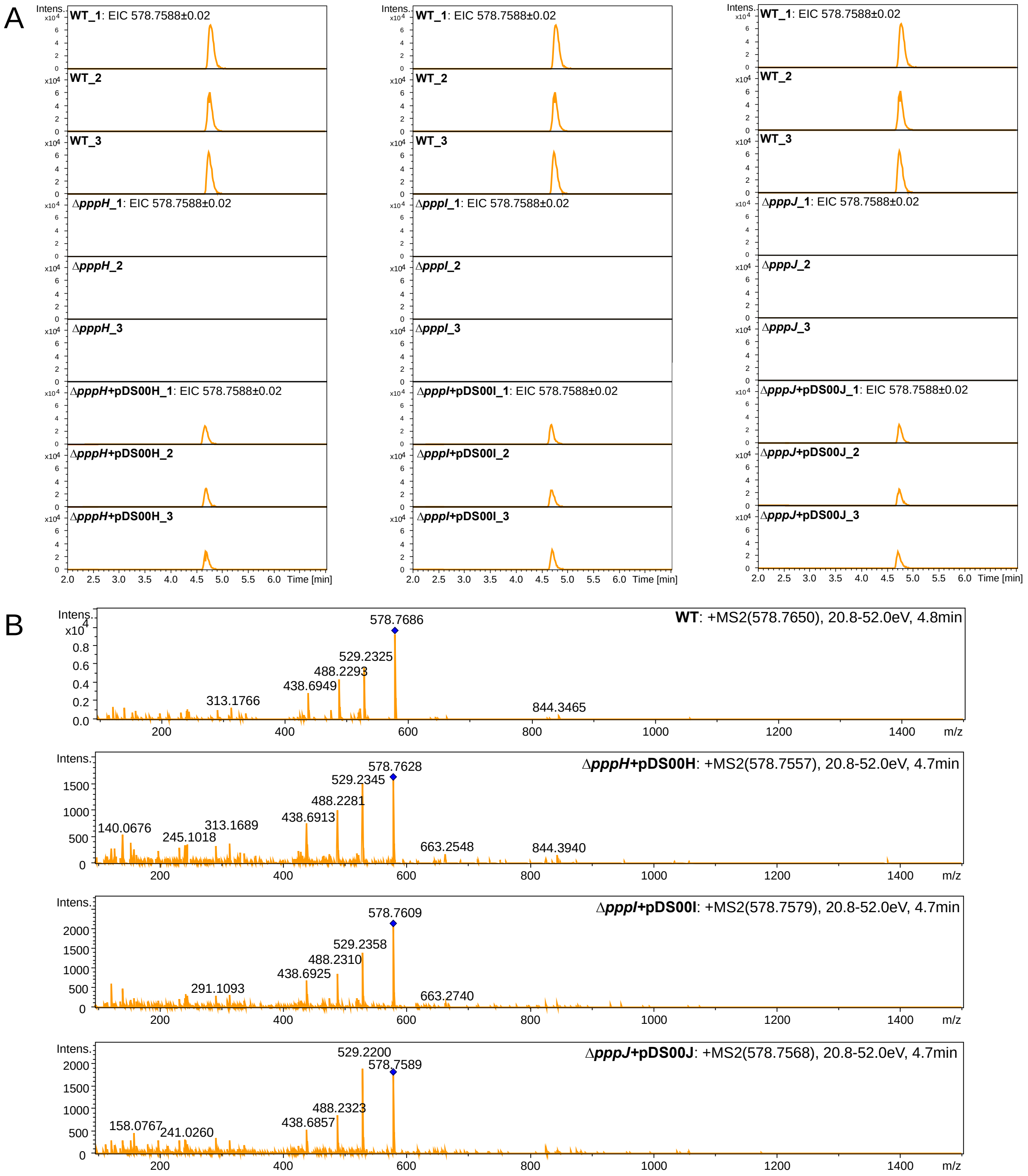

Figure S16. Comparison of PB1 production and MS/MS fragmentation between wild type, Δ*pppH*, Δ*pppI*, Δ*pppJ* mutants, and genetically complemented strains. UPLC-QToF-MS analyses were performed in triplicates. (**A**) Extracted Ion Chromatograms (EIC) of doubly charged PB1 (Val, $[M+2H]^{2+}$). The same mass filter (the expected *m/z* ± 0.02) was applied to all samples.

(**B**) The fragmentation pattern of PB1 produced from WT compared to those of complemented Δ*pppH,* Δ*pppI,* and Δ*pppJ* mutants*.* Expected fragmentation peaks of PB1 are *m/z* 844.34, *m/z* 529.22, *m/z* 488.22, and *m/z* 438.69 (**Fig. S10**). The upper right numbers from left to right are the *m/z* of the fragmentated compound, the collision energy, and the retention time. The blue diamond pinpoints the parent peak.

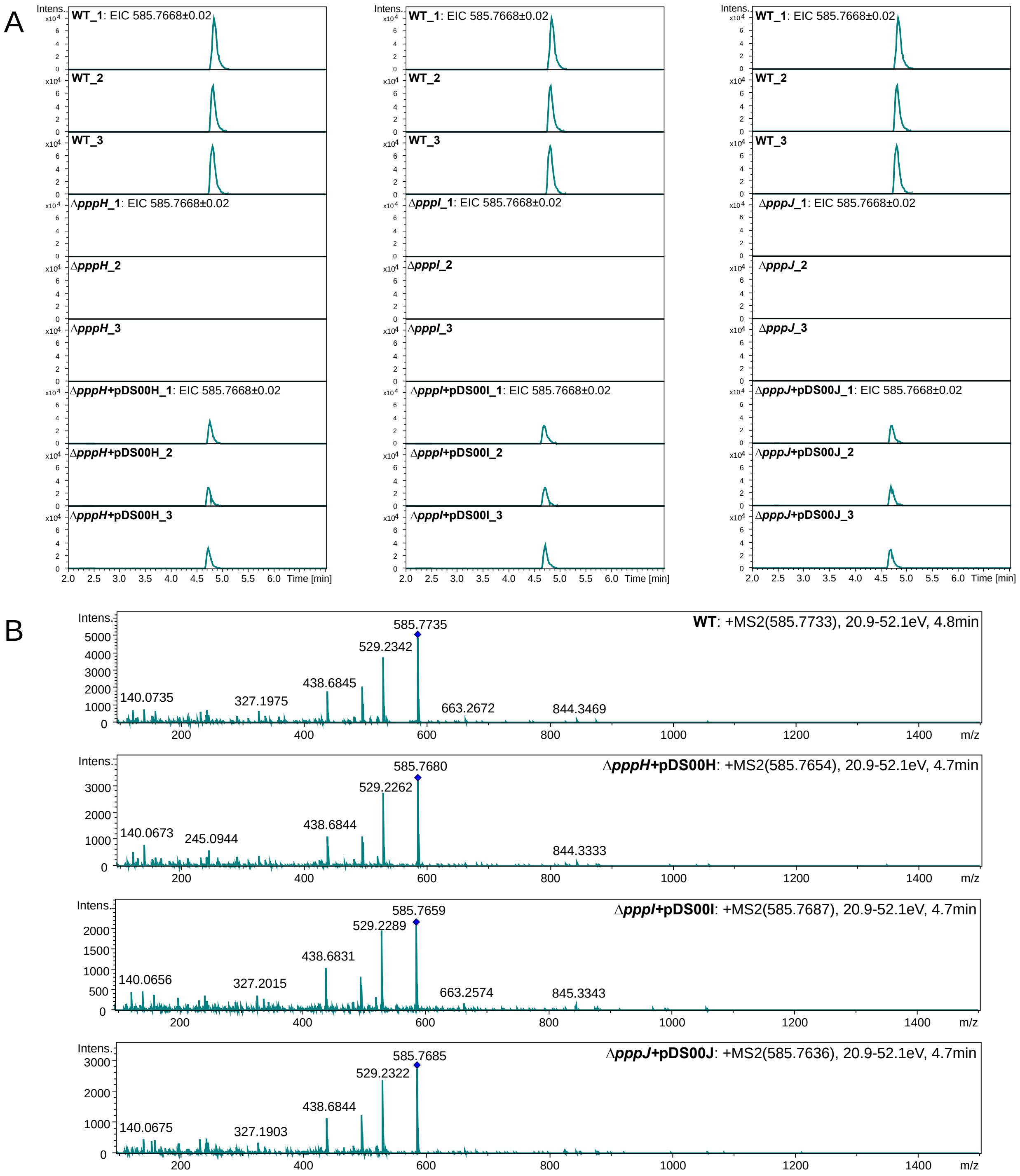

Figure S17. Comparison of PB2 or PB3 production and MS/MS fragmentation between wild type, Δ*pppH,* Δ*pppI,* Δ*pppJ* mutants, and genetically complemented strains. UPLC-QToF-MS analyses were performed in triplicates. (**A**) Extracted Ion Chromatograms (EIC) of doubly charged PB2 or PB3 (Ile or Leu, $[M+2H]^{2+}$). The same mass filter (the expected *m/z* ± 0.02) was

applied to all samples. (**B**) Fragmentation pattern of PB2 or PB3 produced from WT compared to those of complemented ∆*pppH,* ∆*pppI,* and ∆*pppJ* mutants. Expected fragmentation peaks of PB2 or PB3 are *m/z* 844.34, *m/z* 529.22, *m/z* 495.23, and *m/z* 438.69 (**Fig. S11**).The upper right numbers from left to right are the *m/z* of the fragmentated compound, the collision energy, and the retention time. The blue diamond pinpoints the parent peak.

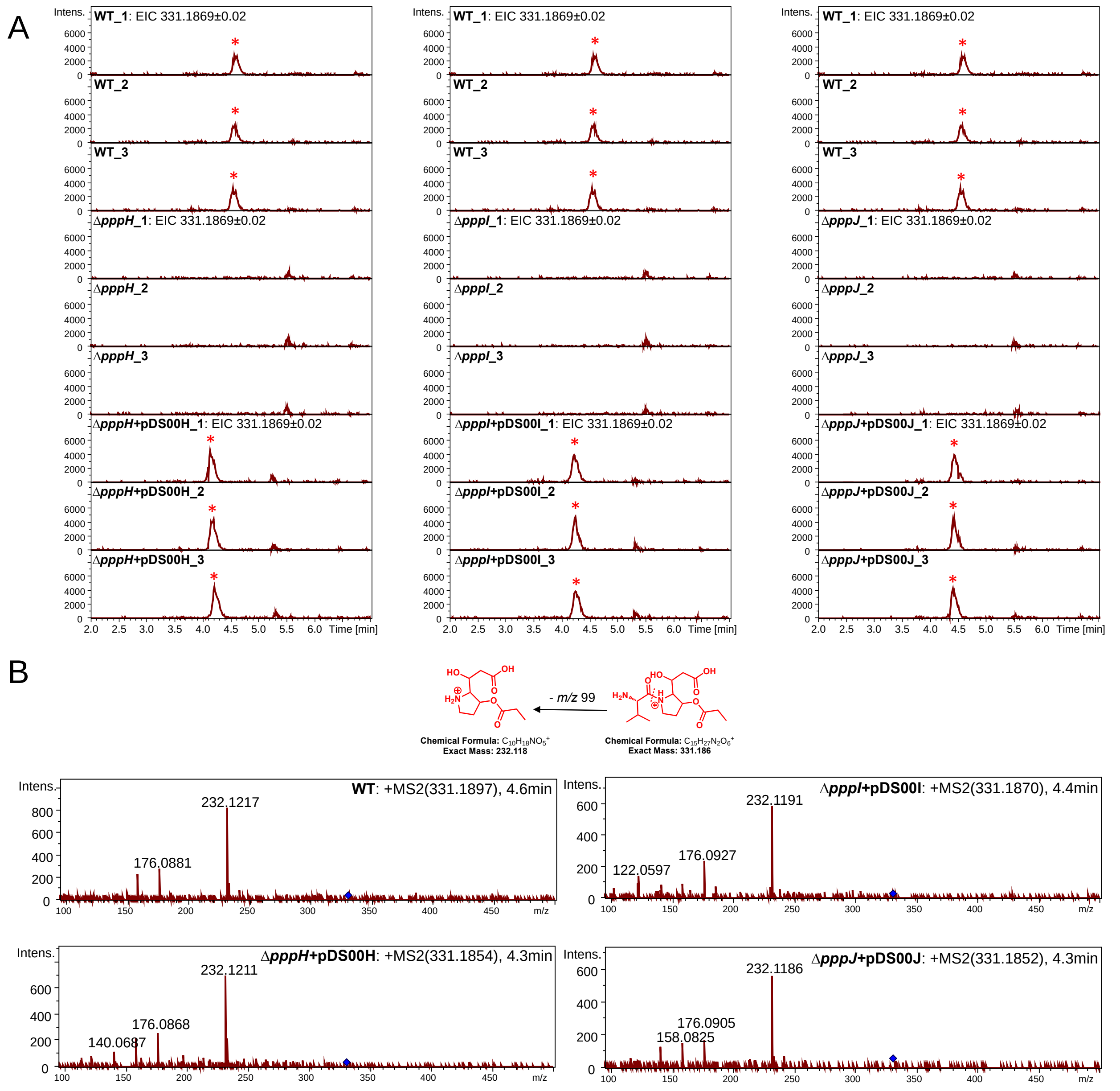

Figure S18. Comparison of PC1 production and MS/MS fragmentation between wild type, Δ*pppH*, Δ*pppI*, Δ*pppJ* mutants, and genetically complemented strains. UPLC-QToF-MS analyses were performed in triplicates. (**A**) Extracted Ion Chromatograms (EIC) of singly charged PC1 (Val, $[M+H]^+$). The same mass filter (the expected *m/z* ± 0.02) was applied to all samples. PC1 peaks are highlighted with a red asterisk. (**B**) Fragmentation pattern of PC1 produced from WT compared to those of Δ*pppH*, Δ*pppI*, and Δ*pppJ* mutants and complemented Δ*pppH*, Δ*pppI*, and Δ*pppJ* mutants. The proposed structures of parent and fragment peaks are shown. The upper

right numbers from left to right are the *m/z* of the fragmentated compound and the retention time. The blue diamond pinpoints the parent peak.

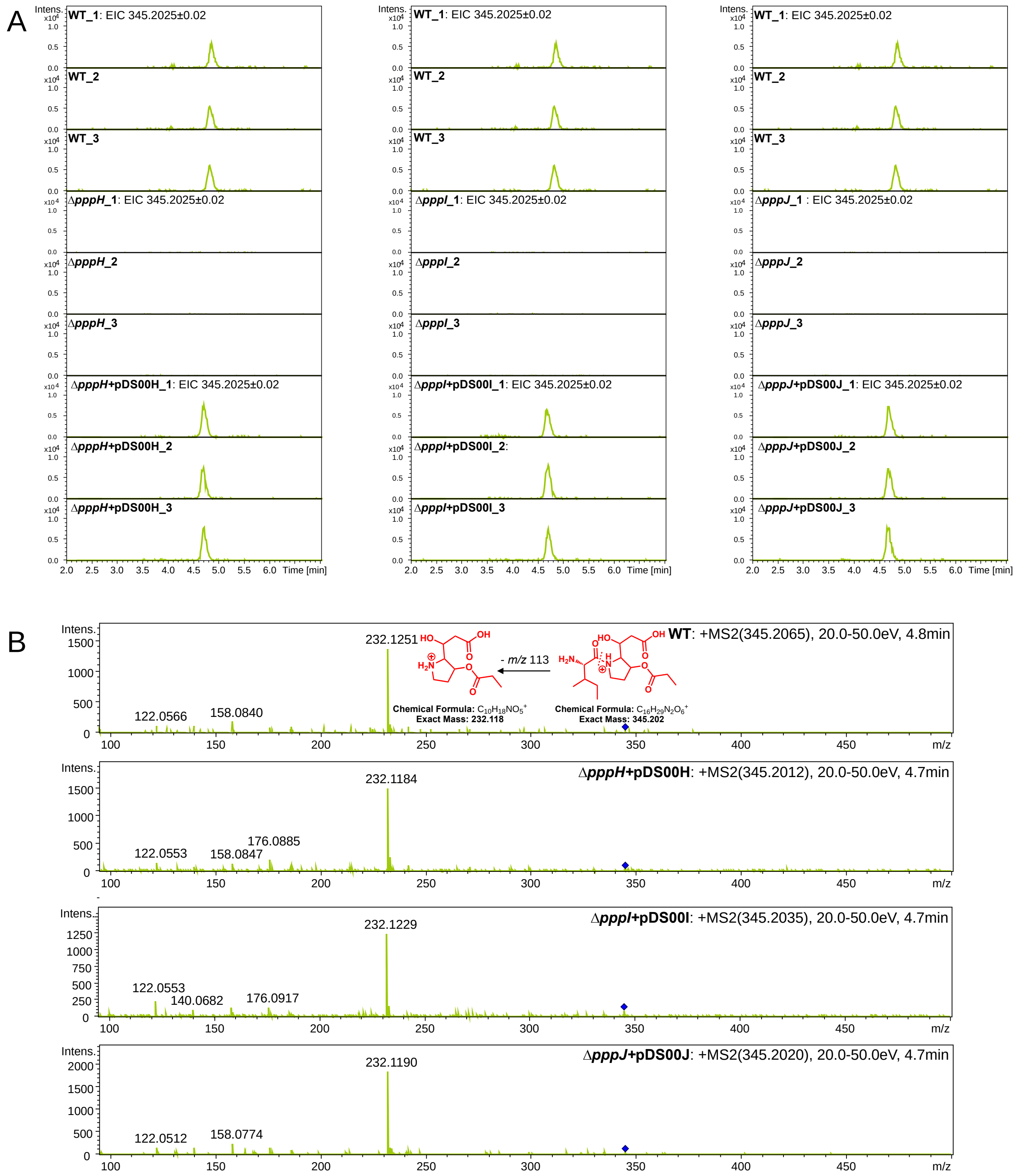

Figure S19. Comparison of PC2 or PC3 production and MS/MS fragmentation between wild type, Δ*pppH,* Δ*pppI,* Δ*pppJ* mutants, and genetically complemented strains. UPLC-QToF-MS analyses were performed in triplicates. (**A**) Extracted Ion Chromatograms (EIC) of singly charged PC2 or PC3 (Leu or Ile, $[M+H]^+$). The same mass filter (the expected *m/z* ± 0.02) was

applied to all samples. (**B**) Fragmentation pattern of PC2 or PC3 produced from WT compared to those of complemented Δ*pppH,* Δ*pppI,* and Δ*pppJ* mutants*.* The proposed structures of parent and fragment peaks are shown. The upper right numbers from left to right are the *m/z* of the fragmentated compound and the retention time. The blue diamond pinpoints the parent peak.

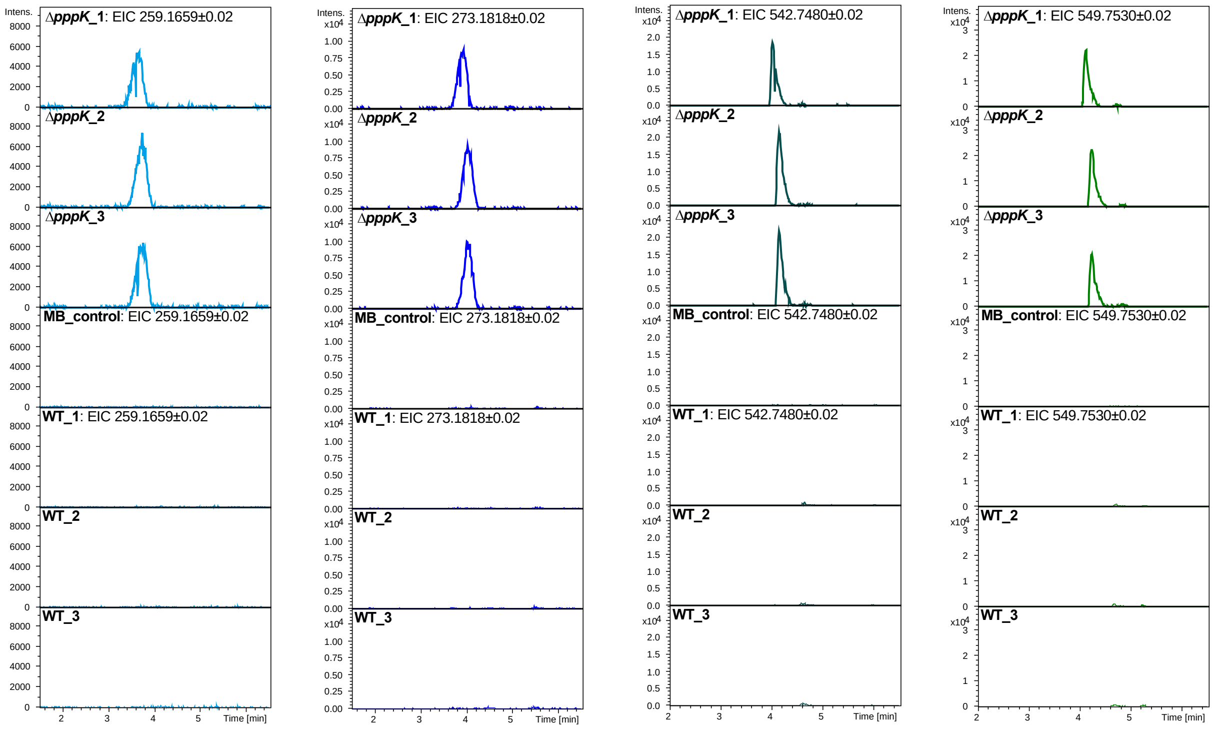

Figure S20. Comparison of depropionylated PBs and PCs production between wild type and Δ*pppK* mutant. UPLC-QToF-MS analyses were performed in triplicates. Extracted Ion Chromatograms (EIC) of (from left to right) depropionylated PCs (Val, $[M+H]^+$ and Ile or Leu, $[M+H]^+$), and depropionylated PBs (Val, $[M+2H]^{2+}$ and Leu or Ile, $[M+2H]^{2+}$). The same mass filter (the expected *m/z* ± 0.02) was applied to all samples.

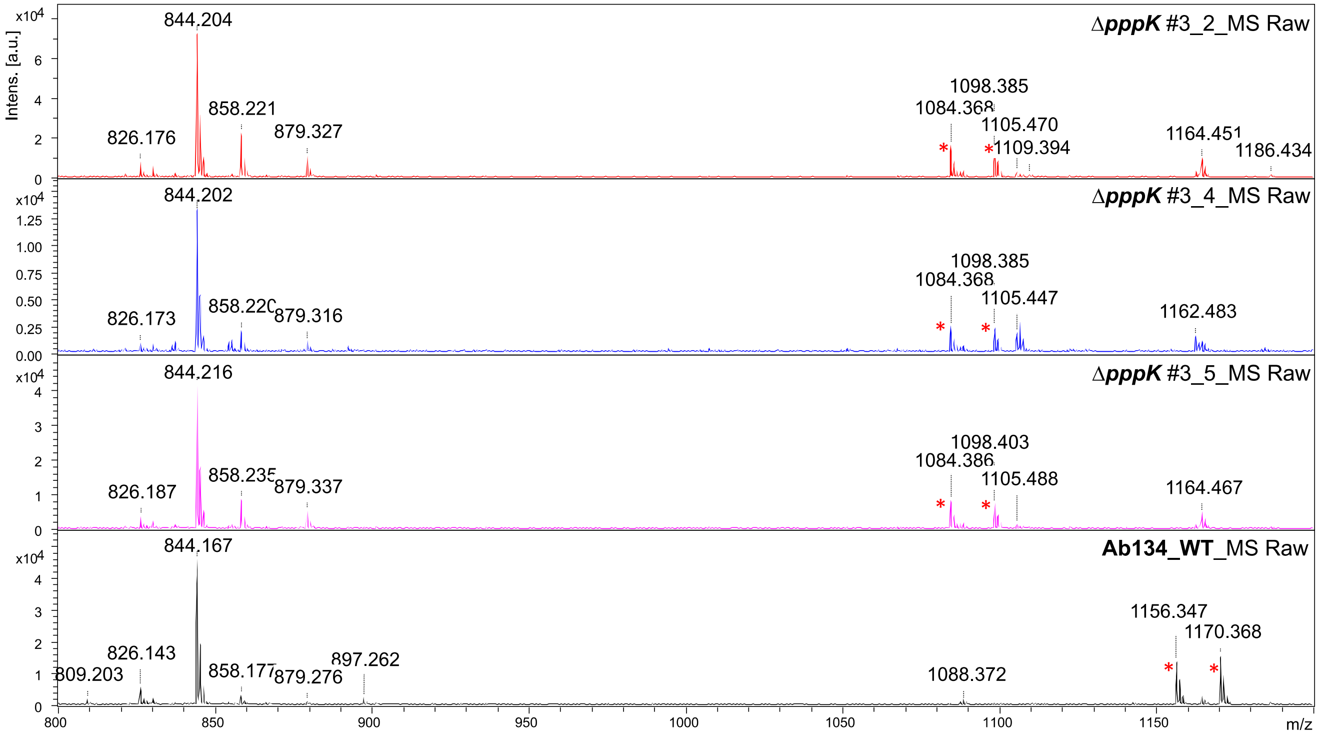

Figure S21. MALDI-ToF MS analyses of wild type and biological triplicates of Δ*pppK* mutants. Mass spectra of WT and Δ*pppK* biological triplicates (#3_2, #3_4, and #3_5). The molecular features representing PBs are indicated with red asterisks. The peak at *m/z* 844.2 represents PA1 ($[M+H]^+$); the peak at *m/z* 1156.3, PB1 (Val, $[M+H]^+$); the peak at *m/z* 1170.4, PB2 or PB3 (Leu or Ile, $[M+H]^+$). The peak at *m/z* 1084.4 represents depropionylated PB1 (Val, $[M+H]^+$); and the peak at *m/z* 1098.4, depropionylated PB2 or PB3 (Leu or Ile, $[M+H]^+$).

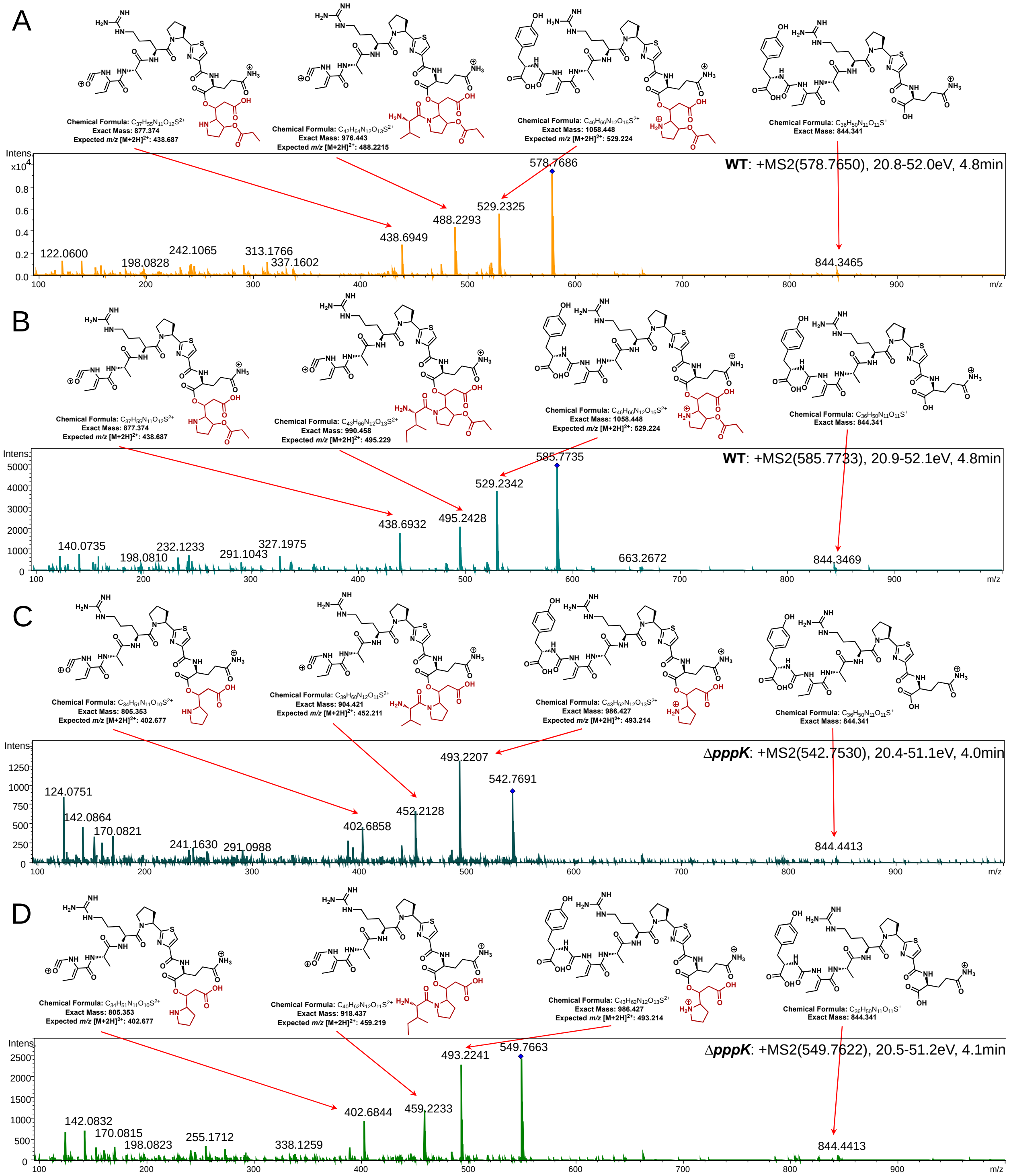

Figure S22. Comparison of MS/MS fragmentation between PBs from wild type and depropionylated PBs from *ΔpppK* mutant. UPLC-QToF-MS/MS analyses were performed for comparing the fragmentation pattern of (**A**) PB1 (Val, $[M+2H]^{2+}$) and (**B**) PB2 or PB3 (Ile or Leu,

$[M+2H]^{2+}$) produced from WT and (**C**) depropionylated PB1 (dPB1, Val, $[M+2H]^{++}$) and (**D**) depropionylated PB2 or PB3 (dPB2, Leu, $[M+2H]^{2+}$ or dPB3, Ile, $[M+2H]^{2+}$) from Δ*pppK*. Proposed structures of major peaks are shown. Structures of PB2 and dPB2 are shown as representatives in (**B**) at *m/z* 495.23 and in (**D**) at *m/z* 459.22. The upper right numbers from left to right are the *m/z* of the fragmentated compound, the collision energy, and the retention time. The blue diamond pinpoints the parent peak.

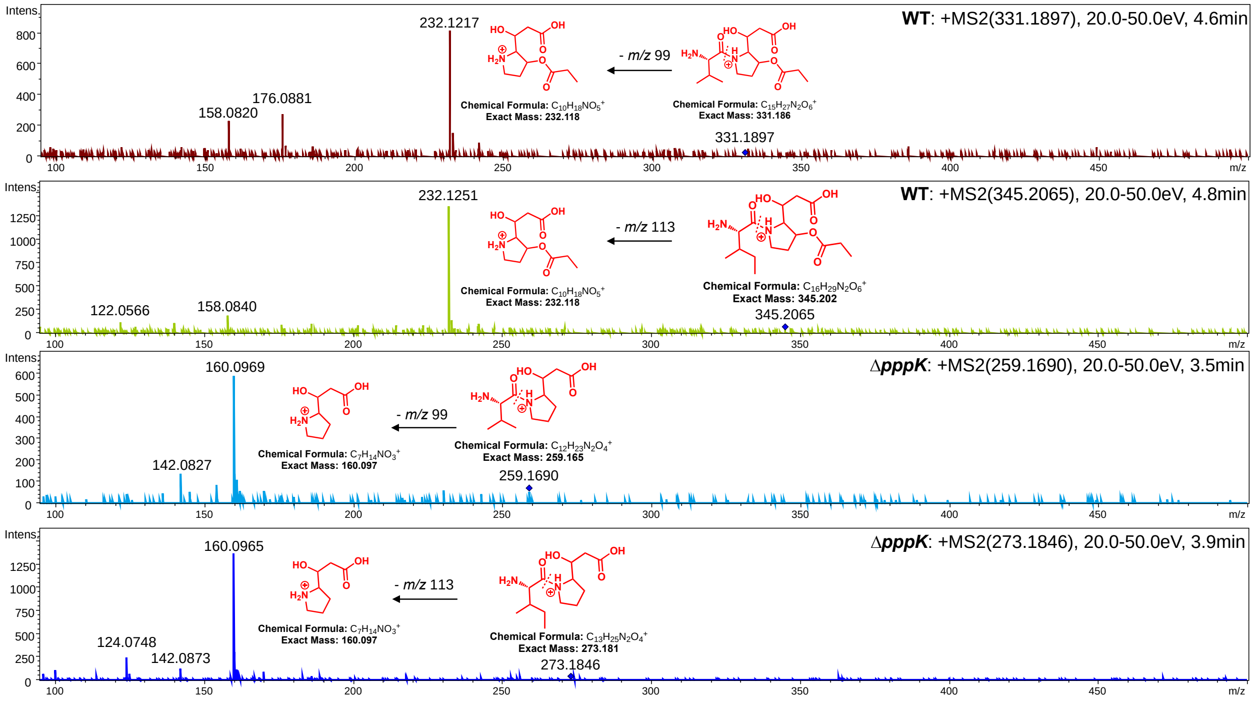

Figure S23. Comparison of MS/MS fragmentation between PCs and depropionylated PCs. UPLC-QToF-MS/MS analyses were performed for comparing the fragmentation pattern (from top to bottom) of PC1 (Val, $[M+H]^+$) and PC2 or PC3 (Ile or Leu, $[M+H]^+$) produced from WT and depropionylated PC1 (dPC1, Val, $[M+H]^+$) and depropionylated PC2 or PC3 (dPC2, Leu, $[M+H]^+$, or dPC3, Leu, $[M+H]^+$) from Δ*pppK.* Predicted singly charged structures are displayed beside peaks. The *m/z* ratio difference between the molecular peak and the base peak corresponds to the molecular weight of valine (PC1 and dPC1) or leucine/isoleucine (PC2 or PC3, and dPC2 or dPC3). The upper right numbers from left to right are the *m/z* of the fragmentated compound, the collision energy, and the retention time. The blue diamond pinpoints the parent peak.

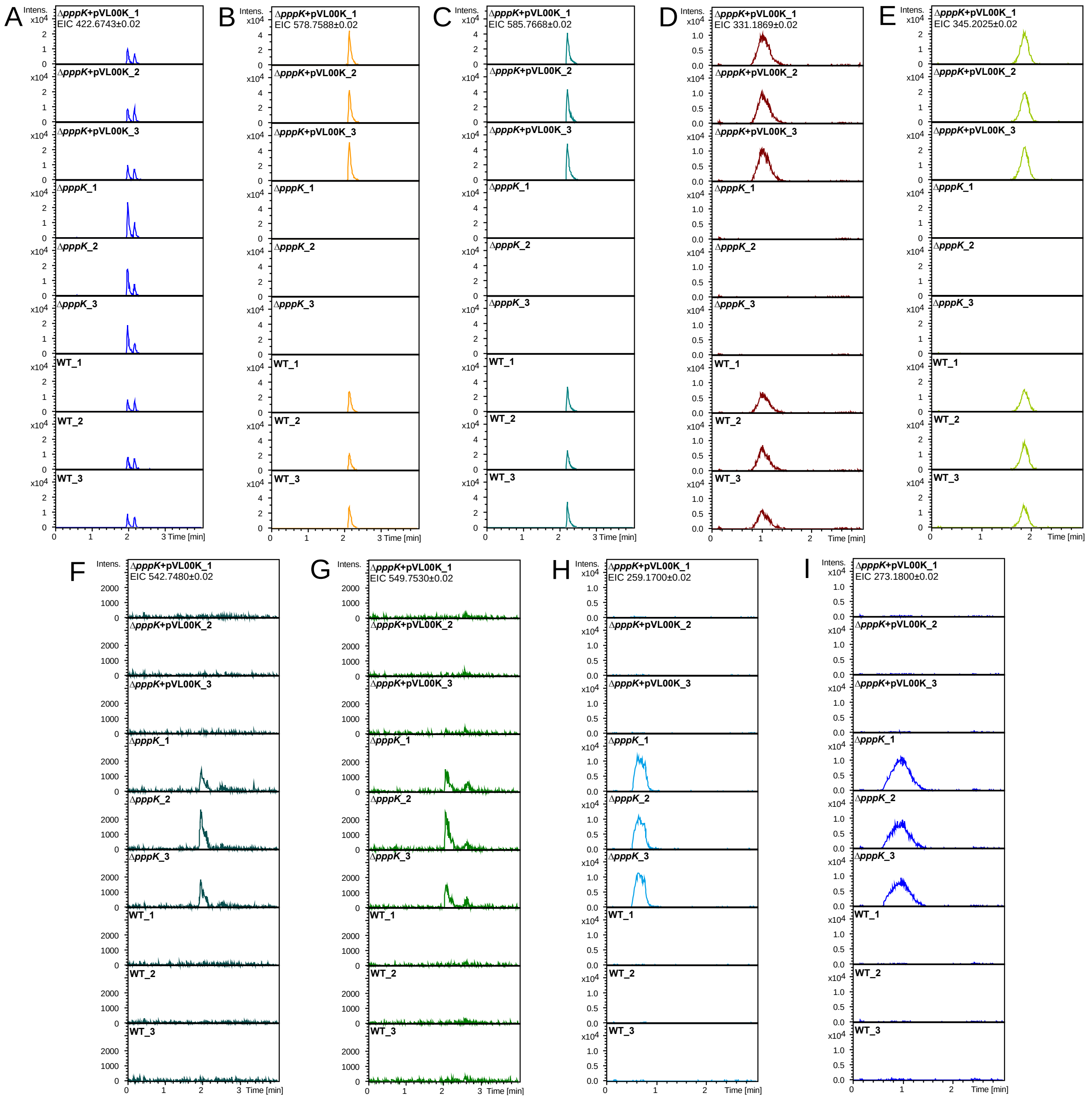

Figure S24. Comparison of PA, PB, and PC production between Δ*pppK* mutant, wild type and genetically complemented ΔpppK mutant. UPLC-QToF-MS analyses were performed for wild type, Δ*pppK* mutant, and complemented Δ*pppK* mutant in triplicates. Extracted Ion Chromatograms (EIC) of (**A**) doubly charged PA1 (*m/z* 442.6743, $[M+2H]^{2+}$), (**B**) doubly charged PB1 (*m/z* 578.7588, Val, $[M+2H]^{2+}$), (**C**) PB2 or PB3 (*m/z* 585.7668, Ile or Leu, $[M+2H]^{2+}$), (**D**) singly charged PC1 (*m/z* 331.1869, Val, $[M+H]^{+}$), (**E**) PC2 or PC3 (*m/z* 345.2025, Ile or Leu, $[M+H]^{+}$), (**F**) doubly charged dPB1 (*m/z* 542.7480, Val, $[M+2H]^{2+}$), (**G**) doubly charged dPB2 or

dPB3 (*m/z* 549.7530, Leu or Ile, $[M+2H]^{2+}$), (**H**) dPC1 (*m/z* 259.1659, Val, $[M+H]^{+}$), and (**I**) dPC2 or dPC3 (*m/z* 273.1818, Leu or Ile, $[M+H]^{+}$). The same mass filter (the expected *m/z* ± 0.02) was applied to all samples.

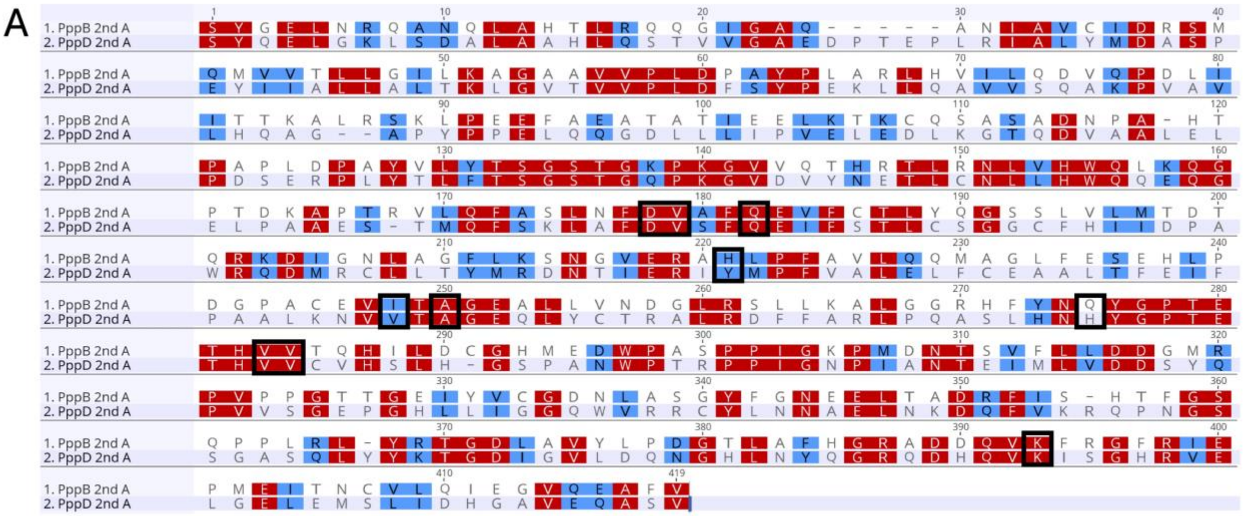

B

Domain	Position 235	236	239	278	299	301	322	330	331	517
Pro	D	V	Q	L	I	A	H	V	V	K
PppB_2nd A	D	V	Q	H	I	A	Q	V	V	K
PppD_2nd A	D	V	Q	Y	V	A	H	V	V	K

Figure S25. Comparison of the second adenylation (A) domains from PppB and PppD. (A) Pairwise alignment of amino acid sequences of second A domains of PppB and PppD. The selectivity-conferring codes of A domains are boxed. Red indicates identical residues, while blue indicates similar residues. (B) Comparison of the 10 selectivity-conferring residues of the A domain activating proline and second A domains from PppB and PppD. Red residues represent variable constituents at ‘wobble’-like positions (23).

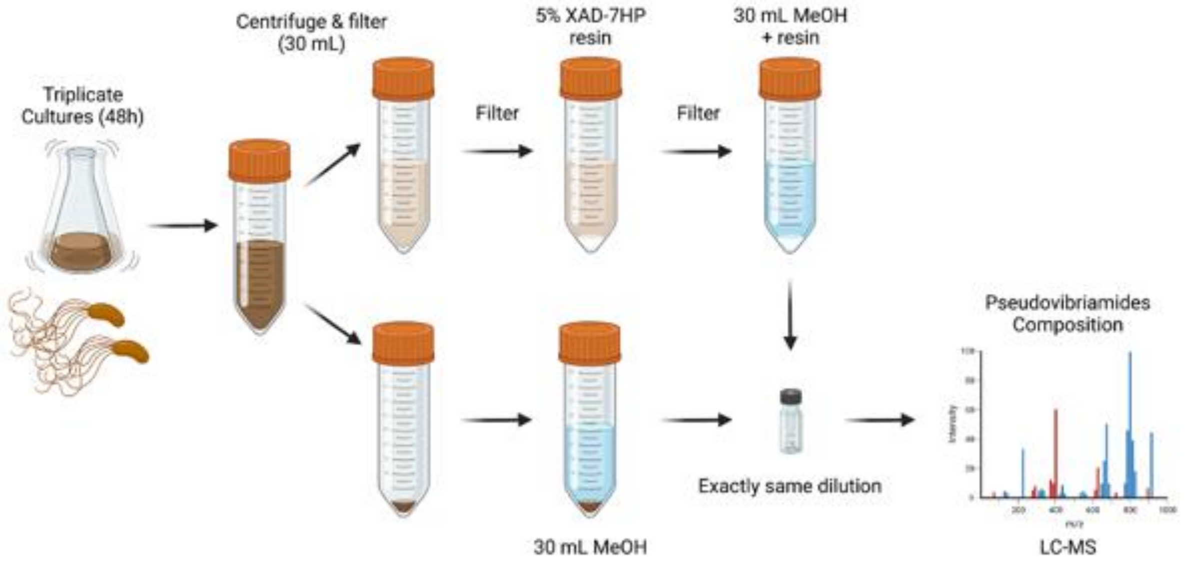

Figure S26. The workflow of pseudovibriamides extraction from cell pellet and supernatant. For the wild type, the Δ*pppG* mutant, the genetically complemented Δ*pppG* mutant, and the Δ*pppL* mutant.

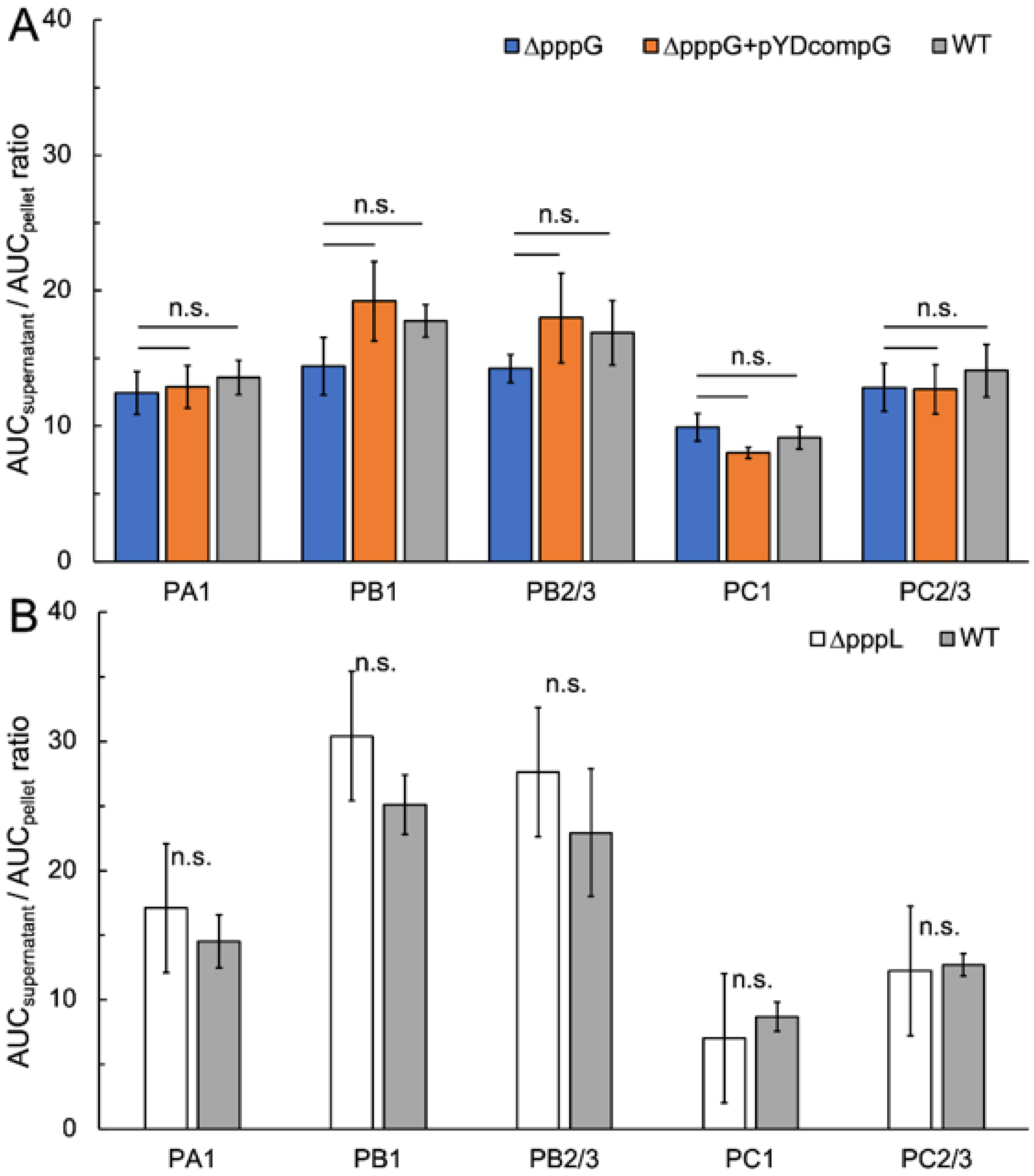

Figure S27. Comparison of pseudovibriamide export ratio between wild type and transporter mutants. Each bar represents the average export ratio of each pseudovibriamide as indicated. (**A**) The average export ratio calculated from triplicate supernatant/pellet extracts was used to assess the relative amount of each pseudovibriamide exported by Δ*pppG,* complemented Δ*pppG* and wild type. Two-tail *P*-values were used to determine statistical significance; n.s., not significant (*P*-value > 0.05). Error bars indicate standard deviation. (**B**) The average export ratio

calculated from quadruplicate supernatant/pellet extracts were used to assess the relative amount of each pseudovibriamide exported in Δ*pppL* and wild type. The same statistical analysis as above was applied.

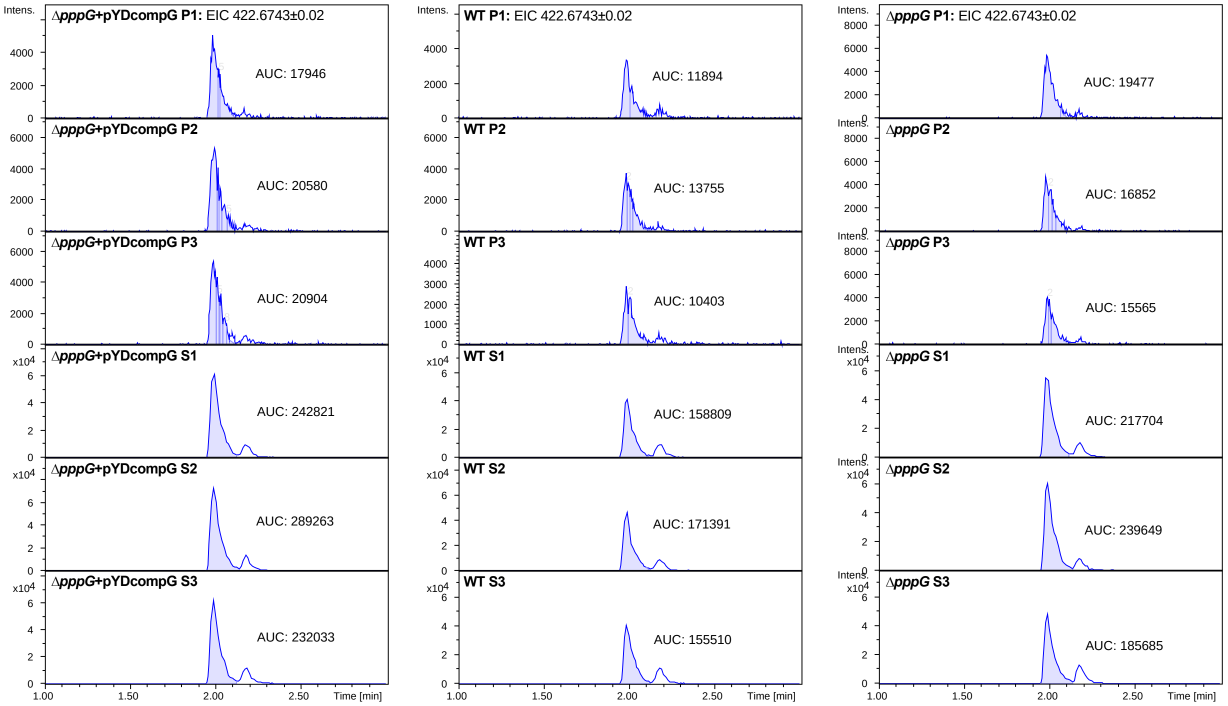

Figure S28. EIC of PA1 from supernatant and pellet extracts of genetically complemented Δ*pppG* strain, wild type, and Δ*pppG* mutant. UPLC-QToF-MS analyses were performed for supernatant and pellet extracts in triplicates. EIC of doubly charged PA1 ($[M+2H]^{2+}$). P, pellet extract; S, supernatant extract; AUC, area under curve. AUC was obtained using “Chromatogram Compound” function, represented by the shaded area.

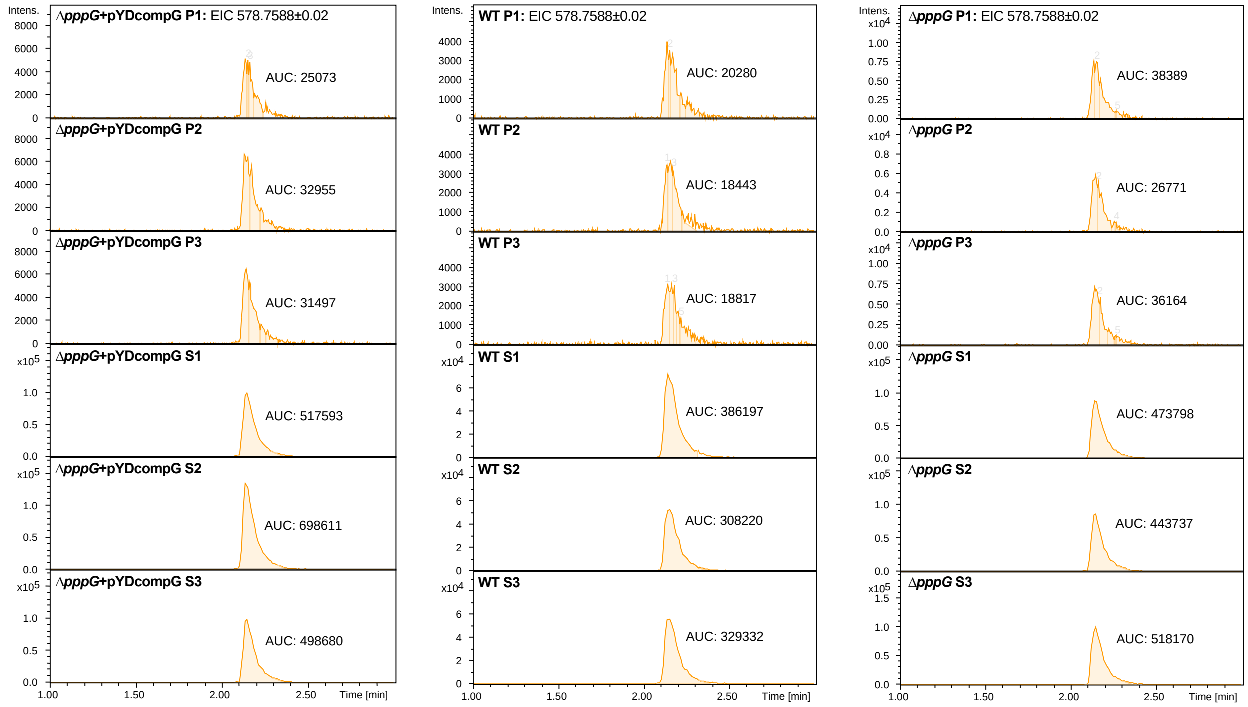

Figure S29. EIC of PB1 from supernatant and pellet extracts of genetically complemented Δ*pppG* strain, wild type, and Δ*pppG* mutant. UPLC-QToF-MS analyses were performed for supernatant and pellet extracts in triplicates. EIC of doubly charged PB1 (Val, $[M+2H]^{2+}$). P, pellet extract; S, supernatant extract; AUC, area under curve. AUC was obtained using “Chromatogram Compound” function, represented by the shaded area.

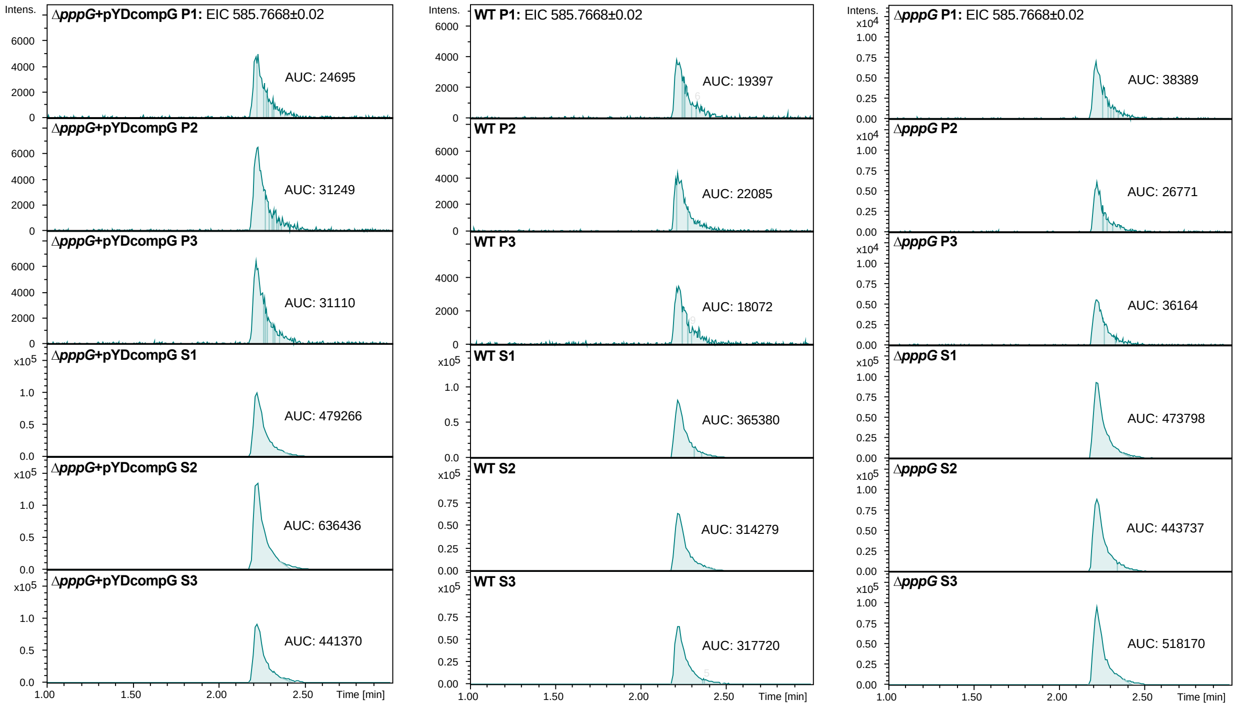

Figure S30. EIC of PB2 or PB3 from supernatant and pellet extracts of genetically complemented Δ*pppG* strain, wild type, and Δ*pppG* mutant. UPLC-QToF-MS analyses were performed for supernatant and pellet extracts in triplicates. EIC of doubly charged PB2 or PB3 (Ile or Leu, $[M+2H]^{2+}$). P, pellet extract; S, supernatant extract; AUC, area under curve. AUC was obtained using “Chromatogram Compound” function, represented by the shaded area.

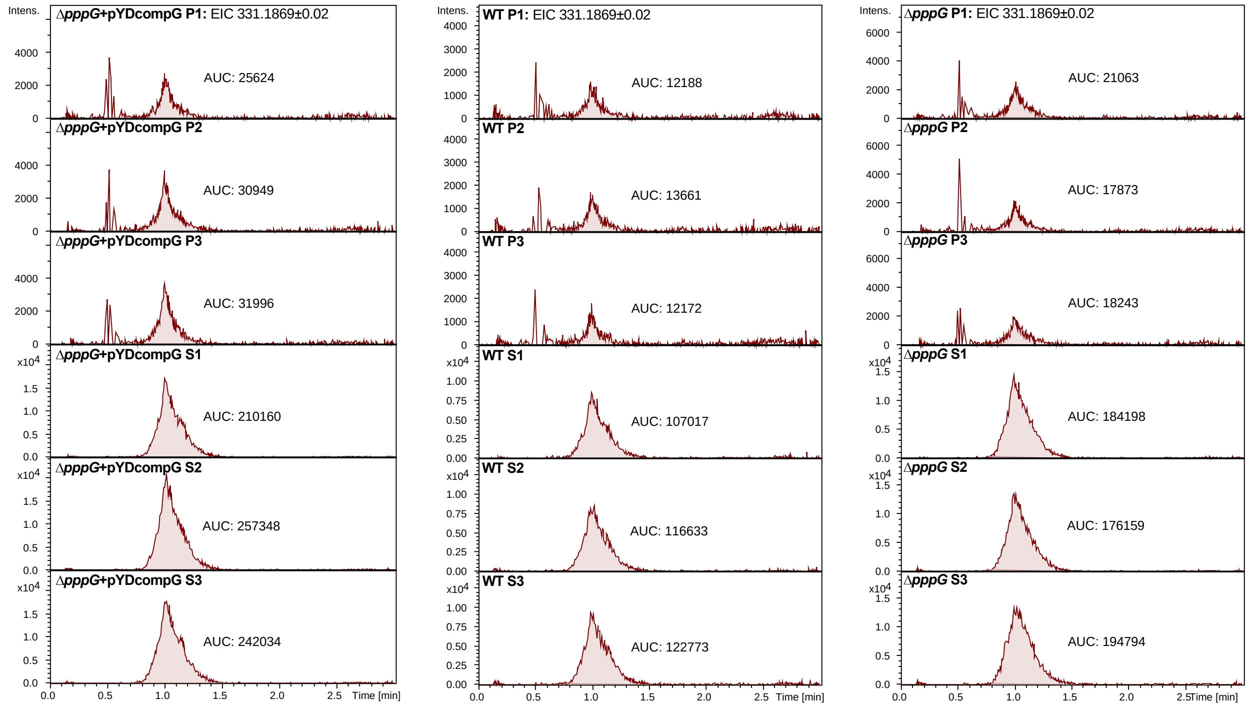

Figure S31. EIC of PC1 from supernatant and pellet extracts of genetically complemented ΔpppG strain, wild type, and Δ*pppG* mutant. UPLC-QToF-MS analyses were performed for supernatant and pellet extracts in triplicates. EIC of singly charged PC1 (Val, $[M+H]^+$). P, pellet extract; S, supernatant extract; AUC, area under curve. AUC was obtained using "Chromatogram Compound" function, represented by the shaded area.

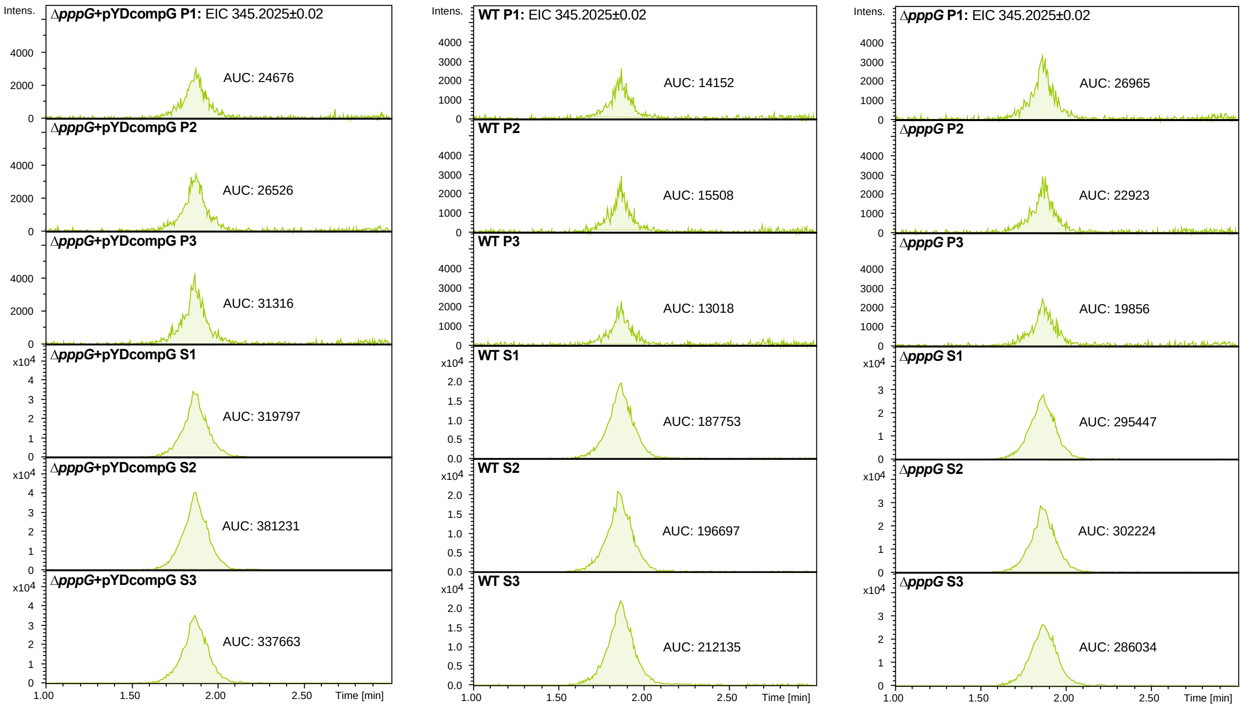

Figure S32. EIC of PC2 or PC3 from supernatant and pellet extracts of genetically complemented Δ*pppG* strain, wild type, and Δ*pppG* mutant. UPLC-QToF-MS analyses were performed for supernatant and pellet extracts in triplicates. EIC of singly charged PC2 or PC3 (Leu or Ile, $[M+H]^+$). P, pellet extract; S, supernatant extract; AUC, area under curve. AUC was obtained using “Chromatogram Compound” function, represented by the shaded area.

Pseudovi-briamide A1	AUC
LP1	1920.3
LP2	1985.7
LP3	1779.2
LP4	2143.1
WP1	5005.2
WP2	4498.2
WP3	4339
WP4	4948.9

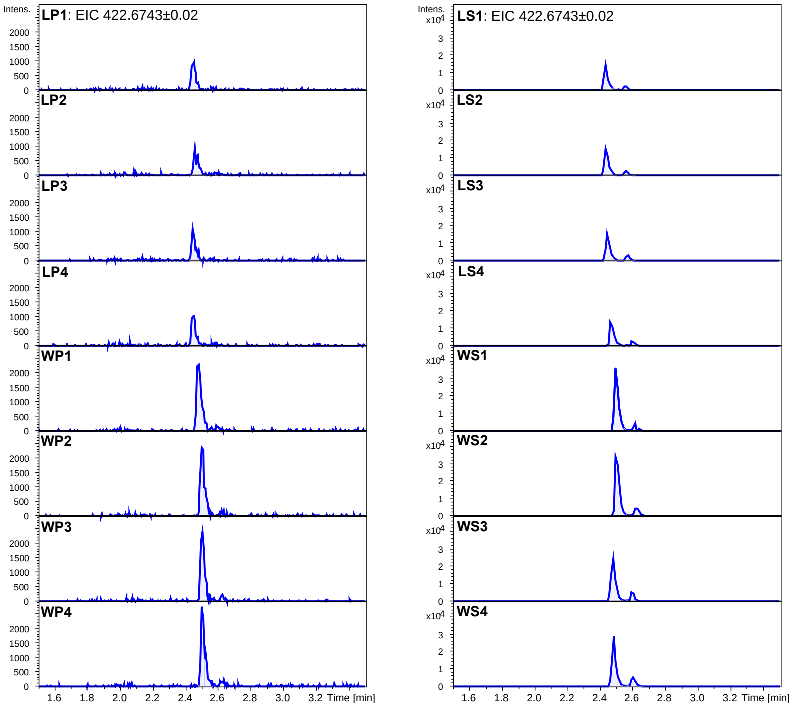

Pseudovi-briamide A1	AUC
LS1	31561.4
LS2	33522.5
LS3	35599.7
LS4	32180.7
WS1	73655.7
WS2	77891
WS3	58383.3
WS4	62277

Figure S33. EIC of PA1 from supernatant and pellet extracts of wild type and Δ*pppL* mutant.

UPLC-QToF-MS analyses were performed for supernatant and pellet extracts from both wild type and Δ*pppL* mutant in quadruplicates. EIC of doubly charged PA1 ($[M+2H]^{2+}$). LP, Δ*pppL* pellet extract; LS, Δ*pppL* supernatant extract; WP, wild type pellet extract; WS, wild type supernatant extract. AUC was obtained using "Chromatogram Compound" function.

Pseudovi-briamide B1	AUC
LP1	2555.6
LP2	2211.7
LP3	2664.5
LP4	2831.6
WP1	6237.4
WP2	6177.4
WP3	5099.8
WP4	6021.3

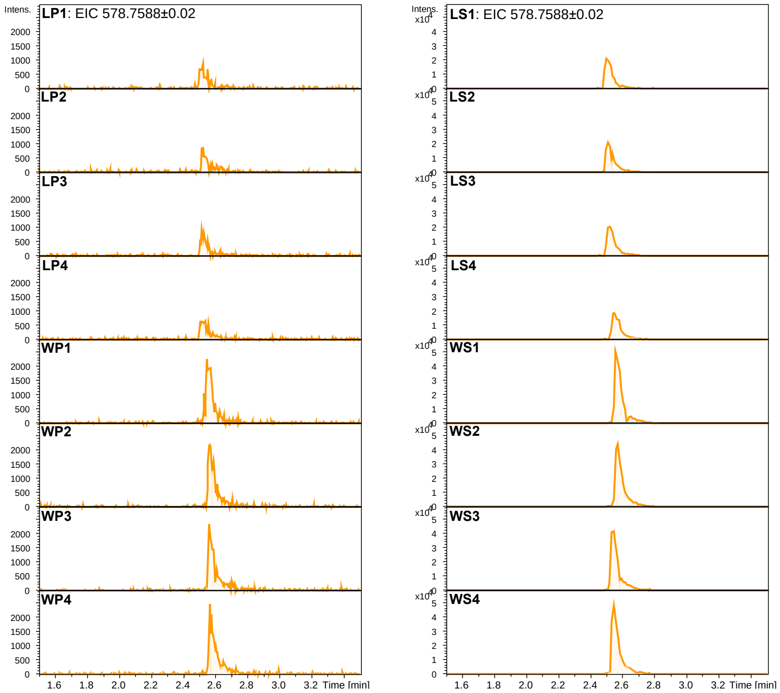

Pseudovi-briamide B1	AUC
LS1	79528.6
LS2	77462.3
LS3	80499.1
LS4	71563.6
WS1	158663.2
WS2	139277.8
WS3	143083.4
WS4	146893.1

Figure S34. EIC of PB1 from supernatant and pellet extracts of wild type and Δ*pppL* mutant. UPLC-QToF-MS analyses were performed for supernatant and pellet extracts from both wild type and Δ*pppL* mutant in quadruplicates. EIC of doubly charged PB1 (Val, $[M+2H]^{2+}$). LP, Δ*pppL* pellet extract; LS, Δ*pppL* supernatant extract; WP, wild type pellet extract; WS, wild type supernatant extract. AUC was obtained using “Chromatogram Compound” function.

Pseudovi-briamide B2/3	AUC
LP1	1857.7
LP2	1963.3
LP3	2403.8
LP4	2322.5
WP1	6183.1
WP2	4797
WP3	5442
WP4	5187.8

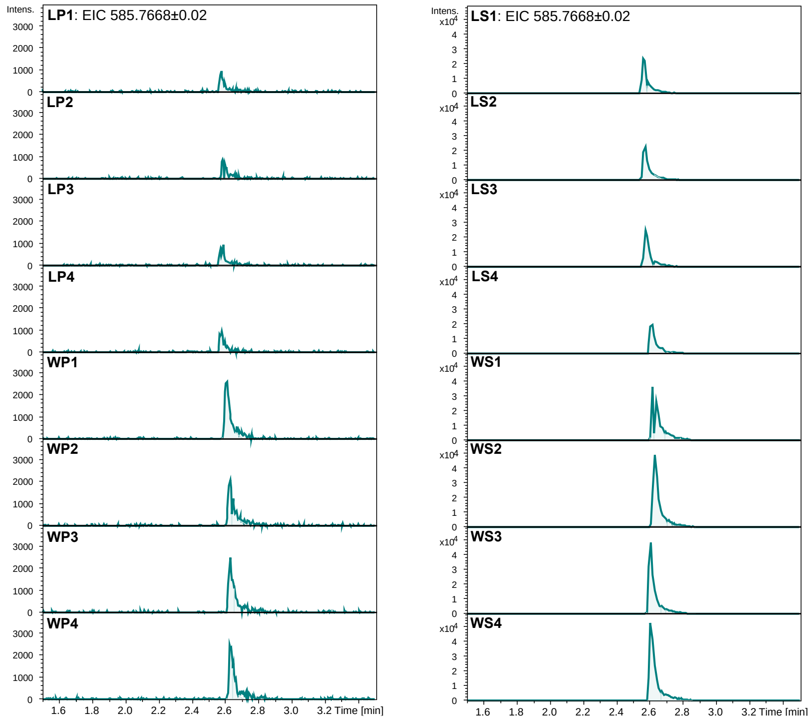

Pseudovi-briamide B2/3	AUC
LS1	62551.2
LS2	58696.8
LS3	51417.6
LS4	59312.8
WS1	98416.9
WS2	125048.9
WS3	125158.5
WS4	138666.5

Figure S35. EIC of PB2 or PB3 from supernatant and pellet extracts of wild type and Δ*pppL* mutant. UPLC-QToF-MS analyses were performed for supernatant and pellet extracts from both wild type and Δ*pppL* mutant in quadruplicates. EIC of doubly charged PB2 or PB3 (Ile or Leu, $[M+2H]^{2+}$). LP, Δ*pppL* pellet extract; LS, Δ*pppL* supernatant extract; WP, wild type pellet extract; WS, wild type supernatant extract. AUC was obtained using “Chromatogram Compound” function.

Pseudovi-briamide C1	AUC
LP1	2281.6
LP2	2078.2
LP3	2014.3
LP4	2573.2
WP1	5249.8
WP2	3634
WP3	4917.8
WP4	4239.3

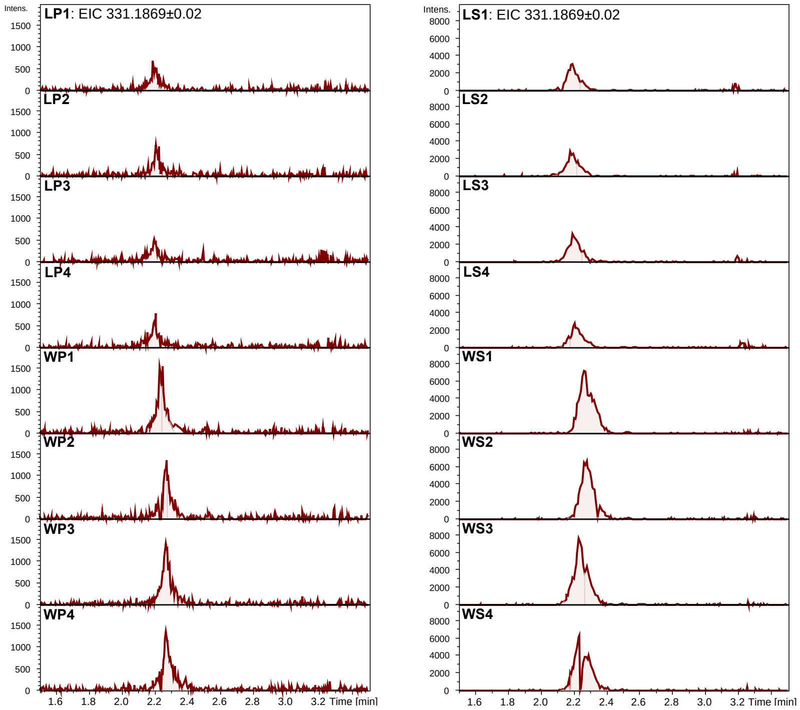

Pseudovi-briamide C1	AUC
LS1	15527.7
LS2	15178
LS3	16596.9
LS4	14818.7
WS1	42620.1
WS2	37514.8
WS3	41612.8
WS4	33424.3

Figure S36. EIC of PC1 from supernatant and pellet extracts of wild type and Δ*pppL* mutant. UPLC-QToF-MS analyses were performed for supernatant and pellet extracts from both wild type and Δ*pppL* mutant in quadruplicates. EIC of singly charged PC1 (Val, $[M+H]^+$). LP, Δ*pppL* pellet extract; LS, Δ*pppL* supernatant extract; WP, wild type pellet extract; WS, wild type supernatant extract. AUC was obtained using “Chromatogram Compound” function.

Pseudovi-briamide C2/3	AUC
LP1	2395.5
LP2	2628.2
LP3	2479.9
LP4	2248.9
WP1	5291.9
WP2	5443.9
WP3	4698.2
WP4	5326.5

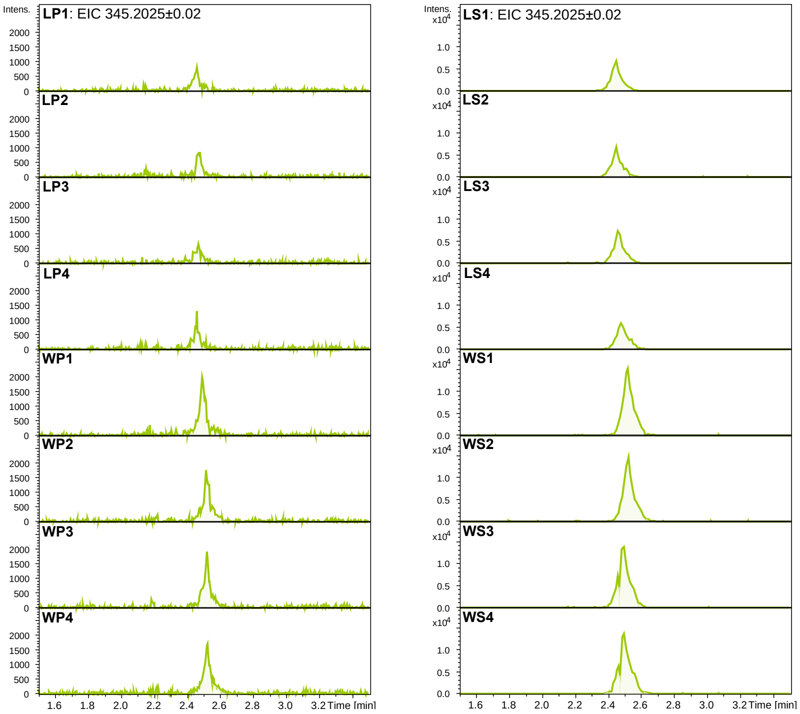

Pseudovi-briamide C2/3	AUC
LS1	29571.7
LS2	27973.3
LS3	32053.7
LS4	29252.5
WS1	68494.1
WS2	65128.7
WS3	64977.1
WS4	64362.9

Figure S37. EIC of PC2 or PC3 from supernatant and pellet extracts of wild type and Δ*pppL* mutant. UPLC-QToF-MS analyses were performed for supernatant and pellet extracts from both wild type and Δ*pppL* mutant in quadruplicates. EIC of singly charged PC2 or PC3 (Leu or Ile, $[M+H]^+$). LP, Δ*pppL* pellet extract; LS, Δ*pppL* supernatant extract; WP, wild type pellet extract; WS, wild type supernatant extract. AUC was obtained using “Chromatogram Compound” function.

A

t-Test: Two-Sample Assuming Equal Variances		
PA1		
	Δ*pppG*	Δ*pppG*+*pYDcompG*
Mean	12.44268	12.89502965
Variance	2.5135	2.486250028
Observations	3	3
Pooled Variance	2.499875	
Hypothesized Mean Differen	0	
df	4	
t Stat	-0.350399	
P(T<=t) one-tail	0.371857	
t Critical one-tail	2.131847	
P(T<=t) two-tail	0.743713	
t Critical two-tail	2.776445	

B

t-Test: Two-Sample Assuming Equal Variances		
PB1		
	Δ*pppG*	Δ*pppG*+*pYDcompG*
Mean	14.41519	19.22499342
Variance	4.485253	8.707890248
Observations	3	3
Pooled Variance	6.596572	
Hypothesized Mean Differenc	0	
df	4	
t Stat	-2.29358	
P(T<=t) one-tail	0.041764	
t Critical one-tail	2.131847	
P(T<=t) two-tail	0.083527	
t Critical two-tail	2.776445	

C

t-Test: Two-Sample Assuming Unequal Variances		
PB2/3		
	Δ*pppG*	Δ*pppG*+*pYDcompG*
Mean	14.24008	17.98705339
Variance	1.080221	11.05809013
Observations	3	3
Hypothesized Mean Differenc	0	
df	2	
t Stat	-1.862782	
P(T<=t) one-tail	0.101764	
t Critical one-tail	2.919986	
P(T<=t) two-tail	0.203529	
t Critical two-tail	4.302653	

D

t-Test: Two-Sample Assuming Unequal Variances		
PC1		
	Δ*pppG*	Δ*pppG*+*pYDcompG*
Mean	9.759722	8.02712732
Variance	0.941016	0.163733112
Observations	3	3
Hypothesized Mean Difference	0	
df	3	
t Stat	2.85513	
P(T<=t) one-tail	0.032418	
t Critical one-tail	2.353363	
P(T<=t) two-tail	0.064835	
t Critical two-tail	3.182446	

E

t-Test: Two-Sample Assuming Equal Variances		
PC2/3		
	Δ*pppG*	Δ*pppG*+*pYDcompG*
Mean	12.84888	12.70466587
Variance	3.057219	3.269551996
Observations	3	3
Pooled Variance	3.163385	
Hypothesized Mean Differenc	0	
df	4	
t Stat	0.099304	
P(T<=t) one-tail	0.462837	
t Critical one-tail	2.131847	
P(T<=t) two-tail	0.925675	
t Critical two-tail	2.776445	

Figure S38. A summary of statistical analyses performed for pseudovibriamides export ratio comparison between Δ*pppG* mutant and complemented Δ*pppG* mutant. (**A**) pseudovibriamide A1; (**B**) pseudovibriamide B1; (**C**) pseudovibriamide B2/3; (**D**) pseudovibriamide C1; (**E**) pseudovibriamide C2/3. P-value of Two-sample two-tail t-Test is used. If the ratio of the larger variance to the smaller variance is less than 4 then assuming the variances are approximately equal or vice versa.

A

t-Test: Two-Sample Assuming Equal Variances		
PA1		
	Δ*pppG*	WT
Mean	12.44268	13.586988
Variance	2.5135	1.5885838
Observations	3	3
Pooled Variance	2.051042	
Hypothesized Mean Differenc	0	
df	4	
t Stat	-0.978593	
P(T<=t) one-tail	0.191595	
t Critical one-tail	2.131847	
P(T<=t) two-tail	0.38319	
t Critical two-tail	2.776445	

B

t-Test: Two-Sample Assuming Equal Variances		
PB1		
	Δ*pppG*	WT
Mean	14.41519	17.75247
Variance	4.485253	1.406649
Observations	3	3
Pooled Variance	2.945951	
Hypothesized Mean Differenc	0	
df	4	
t Stat	-2.381363	
P(T<=t) one-tail	0.037938	
t Critical one-tail	2.131847	
P(T<=t) two-tail	0.075877	
t Critical two-tail	2.776445	

C

t-Test: Two-Sample Assuming Unequal Variances		
PB2/3		
	Δ*pppG*	WT
Mean	14.24008	16.88279
Variance	1.080221	5.670846
Observations	3	3
Hypothesized Mean Differenc	0	
df	3	
t Stat	-1.761666	
P(T<=t) one-tail	0.08817	
t Critical one-tail	2.353363	
P(T<=t) two-tail	0.17634	
t Critical two-tail	3.182446	

D

t-Test: Two-Sample Assuming Equal Variances		
PC1		
	Δ*pppG*	WT
Mean	9.759722	9.135051
Variance	0.941016	0.693882
Observations	3	3
Pooled Variance	0.817449	
Hypothesized Mean Differenc	0	
df	4	
t Stat	0.846188	
P(T<=t) one-tail	0.22255	
t Critical one-tail	2.131847	
P(T<=t) two-tail	0.4451	
t Critical two-tail	2.776445	

E

t-Test: Two-Sample Assuming Equal Variances		
PC2/3		
	Δ*pppG*	WT
Mean	12.84888	14.08208
Variance	3.057219	3.760672
Observations	3	3
Pooled Variance	3.408945	
Hypothesized Mean Difference	0	
df	4	
t Stat	-0.818033	
P(T<=t) one-tail	0.229634	
t Critical one-tail	2.131847	
P(T<=t) two-tail	0.459268	
t Critical two-tail	2.776445	

Figure S39. A summary of statistical analyses performed for pseudovibriamides export ratio comparison between Δ*pppG* mutant and wild type. (**A**) pseudovibriamide A1; (**B**) pseudovibriamide B1; (**C**) pseudovibriamide B2/3; (**D**) pseudovibriamide C1; (**E**) pseudovibriamide C2/3. P-value of Two-sample two-tail t-Test is used. If the ratio of the larger variance to the smaller variance is less than 4 then assuming the variances are approximately equal or vice versa.

A t-Test: Two-Sample Assuming Equal Variances

	AUC_{Sup} / AUC_{pellet} Ratio	
	ΔpppL PA1	*WT PA1*
Mean	17.0855999	14.5178241
Variance	4.43084945	4.2458049
Observations	4	4
Pooled Variance	4.33832717	
Hypothesized Mean Difference	0	
df	6	
t Stat	1.7434558	
P(T<=t) one-tail	0.06593936	
t Critical one-tail	1.94318028	
P(T<=t) two-tail	0.13187871	
t Critical two-tail	2.44691185	

B t-Test: Two-Sample Assuming **Unequal** Variances

	AUC_{Sup} / AUC_{pellet} Ratio	
	ΔpppL PB1	*WT PB1*
Mean	30.4070327	25.1090378
Variance	16.072323	5.2909549
Observations	4	4
Hypothesized Mean Difference	0	
df	5	
t Stat	2.29249072	
P(T<=t) one-tail	0.03521492	
t Critical one-tail	2.01504837	
P(T<=t) two-tail	0.07042985	
t Critical two-tail	2.57058184	

C t-Test: Two-Sample Assuming Equal Variances

	AUC_{Sup} / AUC_{pellet} Ratio	
	ΔpppL PB2/3	*WT PB2/3*
Mean	27.62419968	22.9282923
Variance	28.31588535	24.4896975
Observations	4	4
Pooled Variance	26.40279145	
Hypothesized Mean Difference	0	
df	6	
t Stat	1.2924377	
P(T<=t) one-tail	0.12187237	
t Critical one-tail	1.94318028	
P(T<=t) two-tail	0.24374474	
t Critical two-tail	2.44691185	

D t-Test: Two-Sample Assuming Equal Variances

	AUC_{Sup} / AUC_{pellet} Ratio	
	ΔpppL PC1	*WT PC1*
Mean	7.0268631	8.69692961
Variance	1.067950189	1.23176821
Observations	4	4
Pooled Variance	1.149859198	
Hypothesized Mean Difference	0	
df	6	
t Stat	-2.20255333	
P(T<=t) one-tail	0.03492803	
t Critical one-tail	1.94318028	
P(T<=t) two-tail	0.06985605	
t Critical two-tail	2.44691185	

E t-Test: Two-Sample Assuming Equal Variances

	AUC_{Sup} / AUC_{pellet} Ratio	
	ΔpppL PC2/3	*WT PC2/3*
Mean	12.2302697	12.7051177
Variance	1.2060375	0.7528973
Observations	4	4
Pooled Variance	0.9794674	
Hypothesized Mean Difference	0	
df	6	
t Stat	-0.67853871	
P(T<=t) one-tail	0.26136265	
t Critical one-tail	1.94318028	
P(T<=t) two-tail	0.5227253	
t Critical two-tail	2.44691185	

Figure S40. A summary of statistical analyses performed for pseudovibriamides export ratio comparison between Δ*pppL* mutant and wild type. (**A**) pseudovibriamide A1; (**B**) pseudovibriamide B1; (**C**) pseudovibriamide B2/3; (**D**) pseudovibriamide C1; (**E**) pseudovibriamide C2/3. P-value of Two-sample two-tail t-Test is used. If the ratio of the larger variance to the smaller variance is less than 4 then assuming the variances are approximately equal or vice versa.

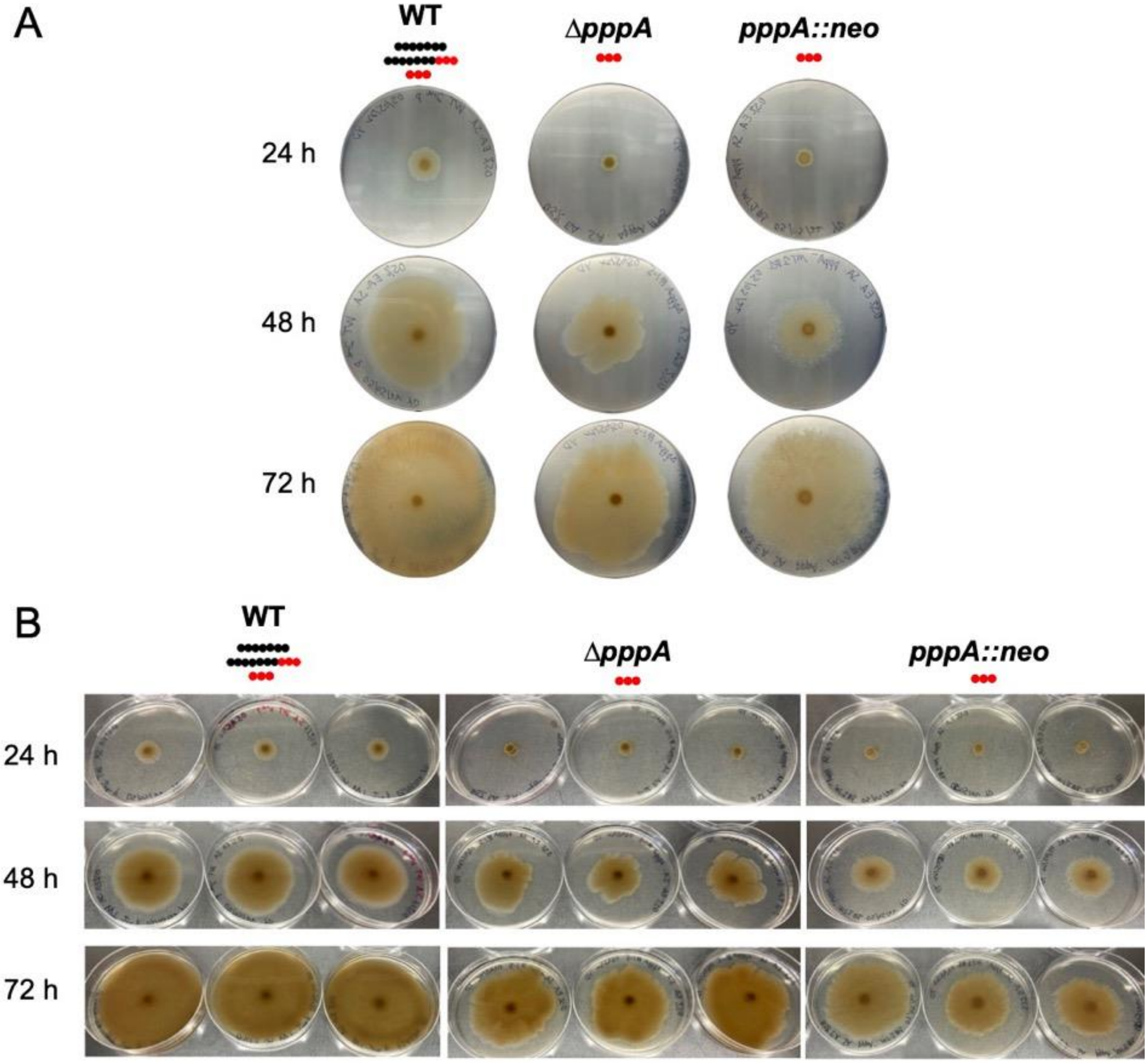

Figure S41. Swarming assay results of ∆*pppA* and *pppA::neo* mutants compared to the wild type. The swarming assay was performed as described under Methods. Pictures shown were taken at 24, 48, and 72 hours after inoculation. (**A**) Representative results are shown. Pseudovibriamides are represented by beads: PA, seven black beads; PB, seven black beads and three red beads; PC, three red beads. (**B**) Triplicates of each strain are shown.

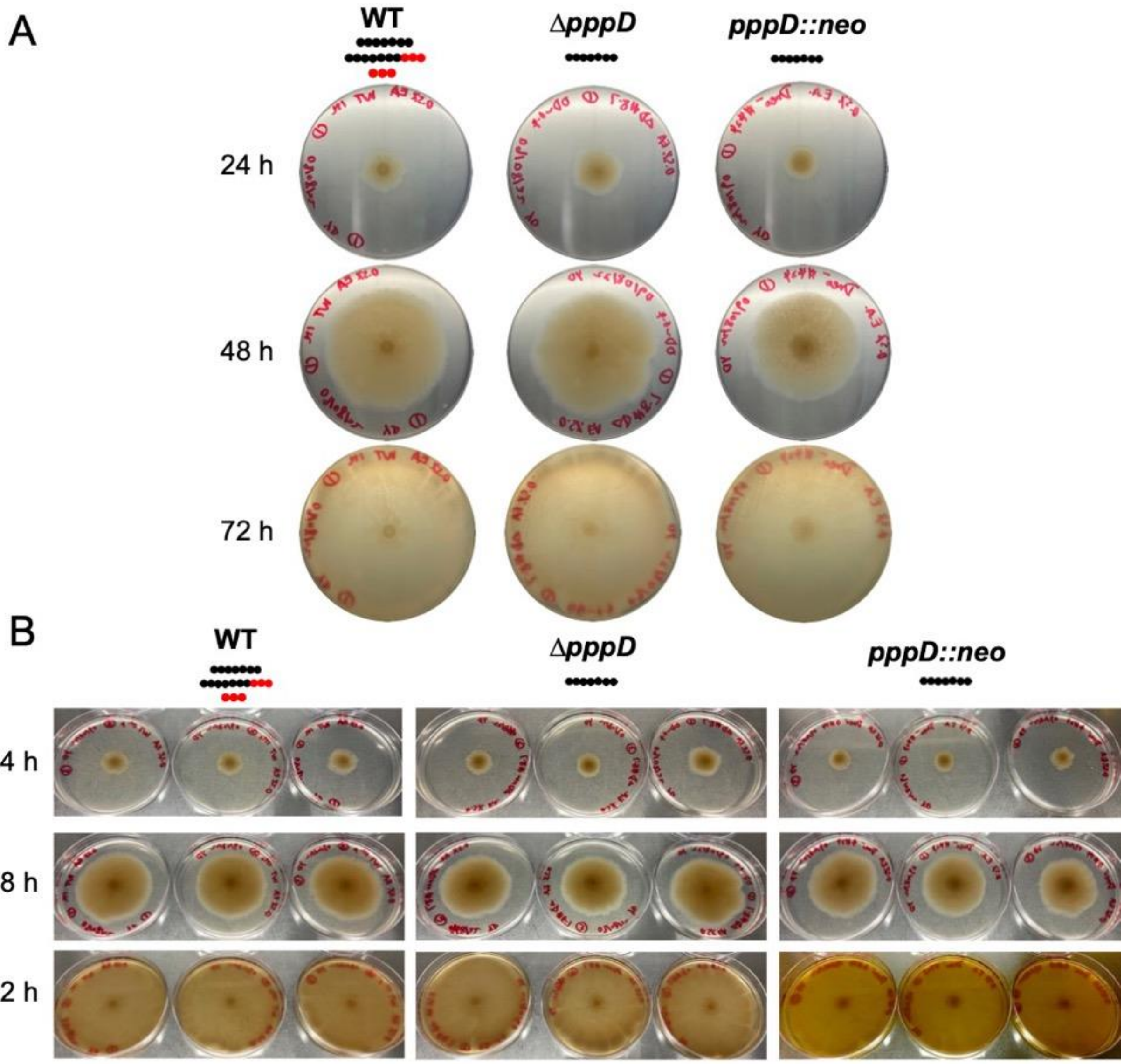

Figure S42. Swarming assay results of Δ*pppD* and *pppD::neo* mutants compared to the wild type. The swarming assay was performed as described under Methods. Pictures shown were taken at 24, 48, and 72 hours after inoculation. (**A**) Representative results are shown. Pseudovibriamides are represented by beads: PA, seven black beads; PB, seven black beads and three red beads; PC, three red beads. (**B**) Triplicates of each strain are shown.

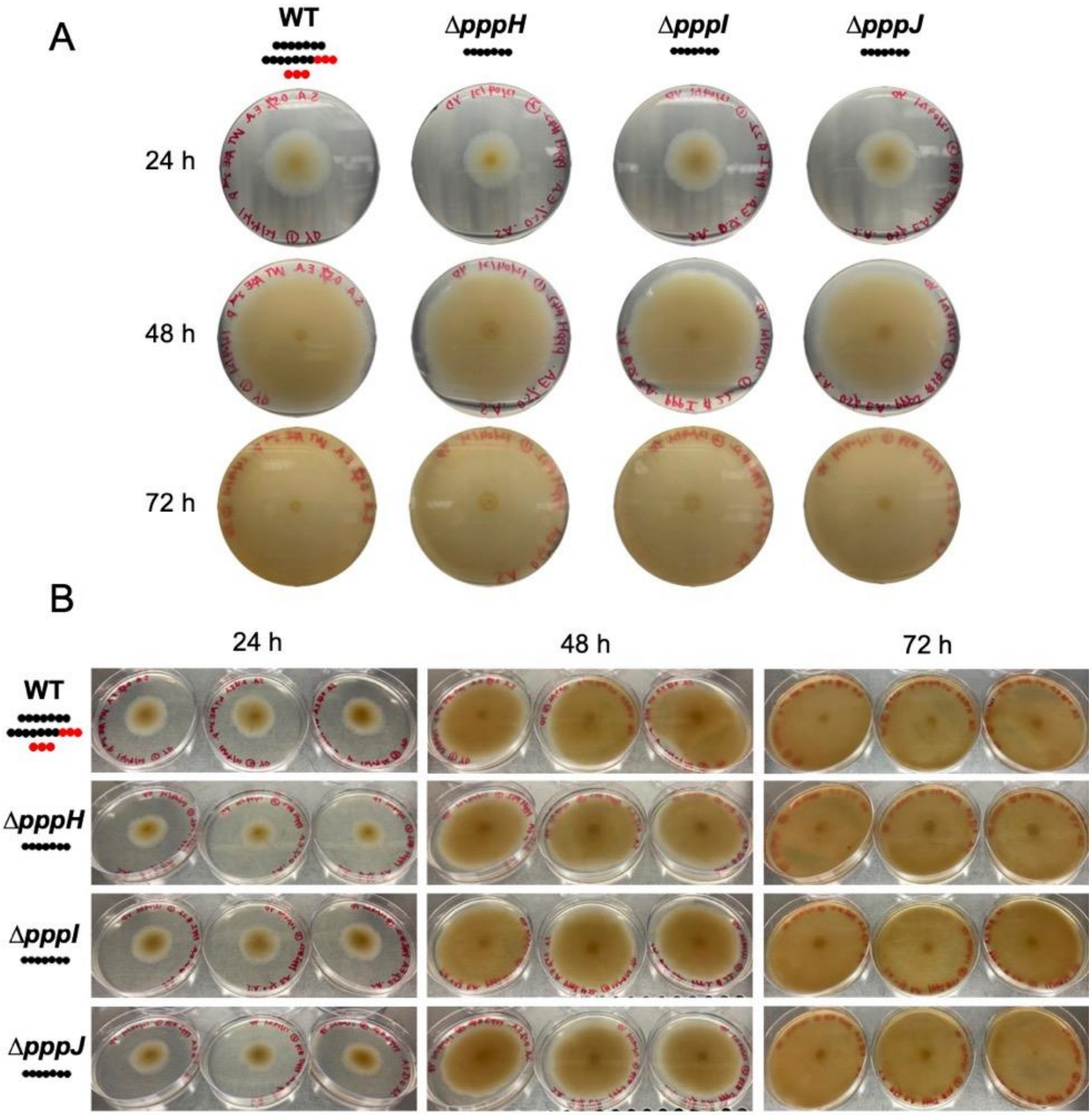

Figure S43. Swarming assay results of Δ*pppH,* Δ*pppI,* and Δ*pppJ* mutants compared to the wild type. The swarming assay was performed as described under Methods. Pictures shown were taken at 24, 48, and 72 hours after inoculation. (**A**) Representative results are shown. Pseudovibriamides are represented by beads: PA, seven black beads; PB, seven black beads and three red beads; PC, three red beads. (**B**) Triplicates of each strain are shown.

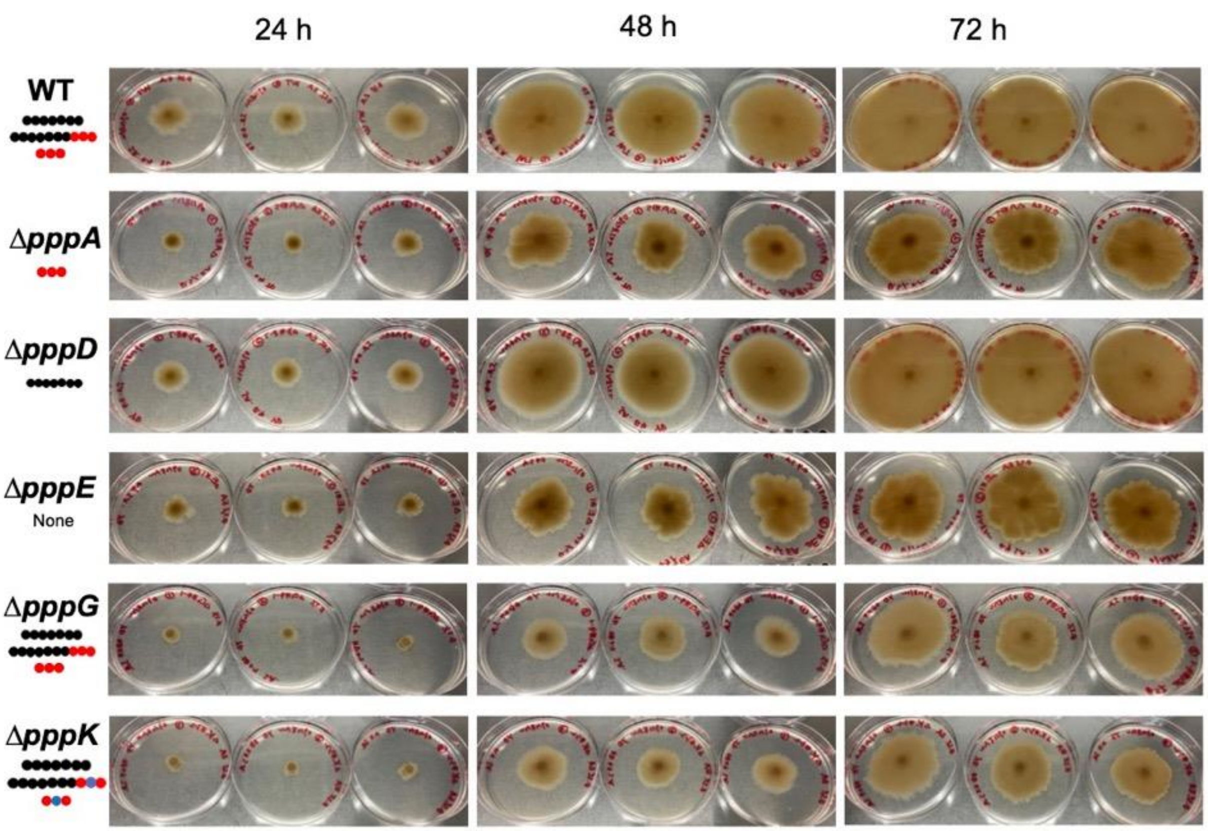

Figure S44. Triplicate swarming assay results of Δ*pppA,* Δ*pppD,* Δ*pppE,* Δ*pppG,* and Δ*pppK* mutants compared to the wild type. The swarming assay was performed as described under Methods. Pictures shown were taken at 24, 48, and 72 hours after inoculation. Pseudovibriamides are represented by beads: PA, seven black beads; PB, seven black beads and three red beads; PC, three red beads; dPB, seven black beads and two red beads with one blue bead in the middle; and dPC, two red beads with one blue bead in the middle. Triplicates of each strain are shown.

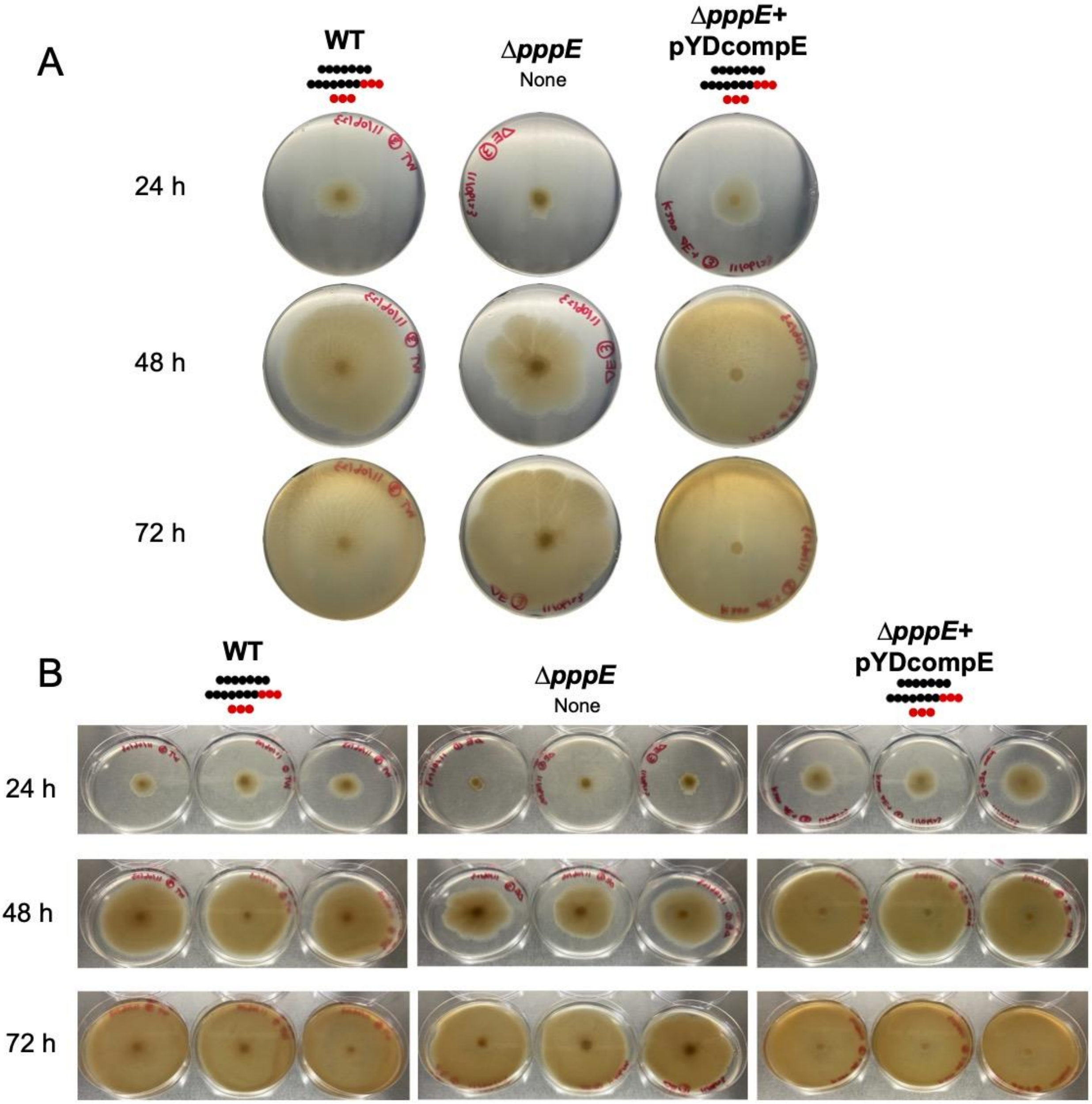

Figure S45. Swarming assay results of Δ*pppE* mutant compared to the wild type and the genetically complemented strain. The swarming assay was performed as described under Methods. Pictures shown were taken at 24, 48, and 72 hours after inoculation. (**A**) Representative results are shown. Pseudovibriamides are represented by beads: PA, seven black beads; PB, seven black beads and three red beads; PC, three red beads. Δ*pppE*+pYDcompE, complemented Δ*pppE* mutant using plasmid pYDcompE. (**B**) Triplicates of each strain are shown.

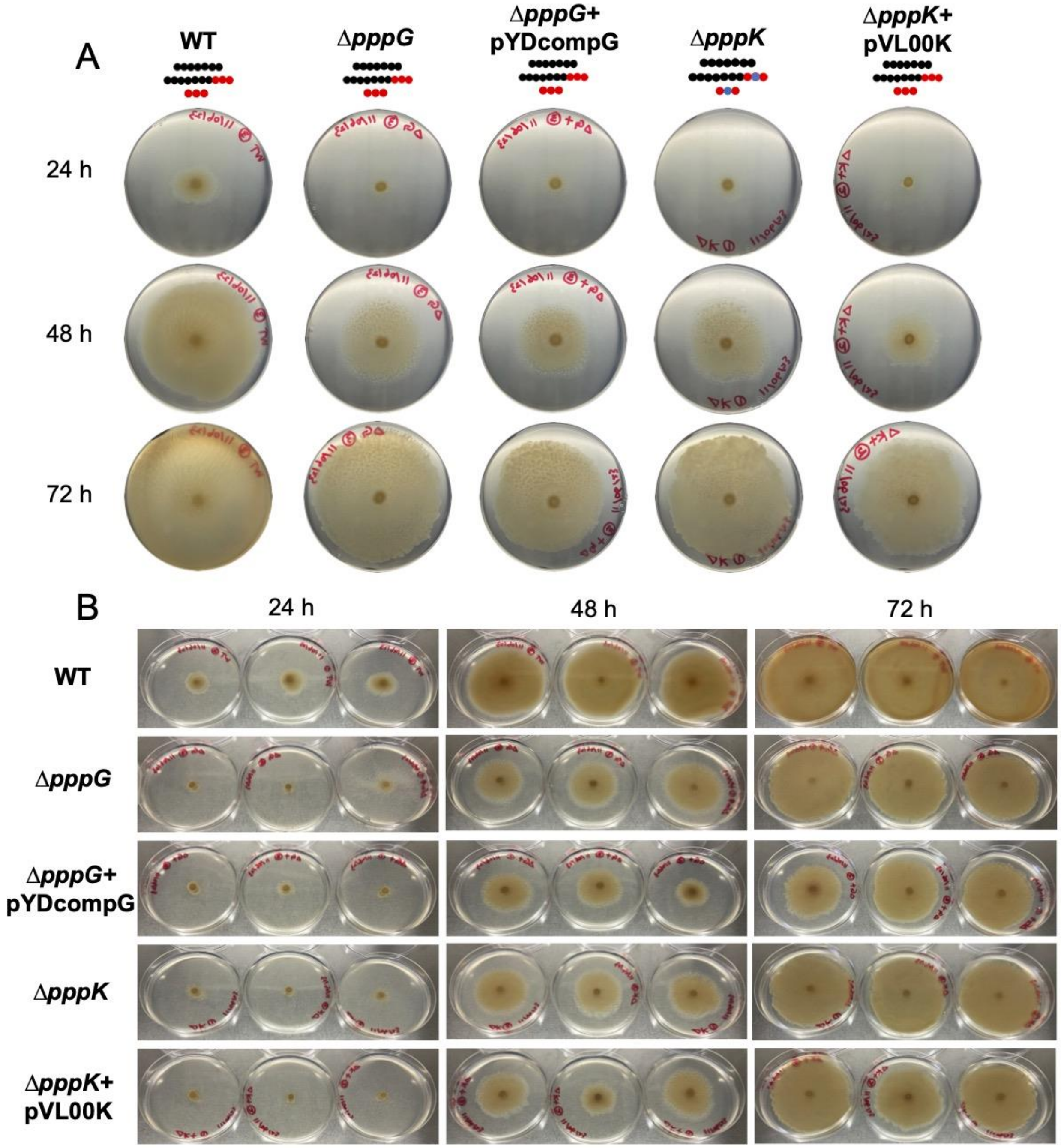

Figure S46. Swarming assay results of Δ*pppG* and Δ*pppK* mutants compared to the wild type and genetically complemented strains. The swarming assay was performed as described under Methods. Pictures shown were taken at 24, 48, and 72 hours after inoculation. (**A**) Representative results are shown. Pseudovibriamides are represented by beads: PA, seven black

beads; PB, seven black beads and three red beads; PC, three red beads; dPB, seven black beads and two red beads with one blue bead in the middle; dPC, two red beads with one blue bead in the middle; Δ*pppK*+pVL00K, trans-complemented Δ*pppK* mutant using plasmid pVL00K; Δ*pppG*+pYDcompG, trans-complemented Δ*pppG* mutant using plasmid pYDcompG. (**B**) Triplicates of each strain are shown.

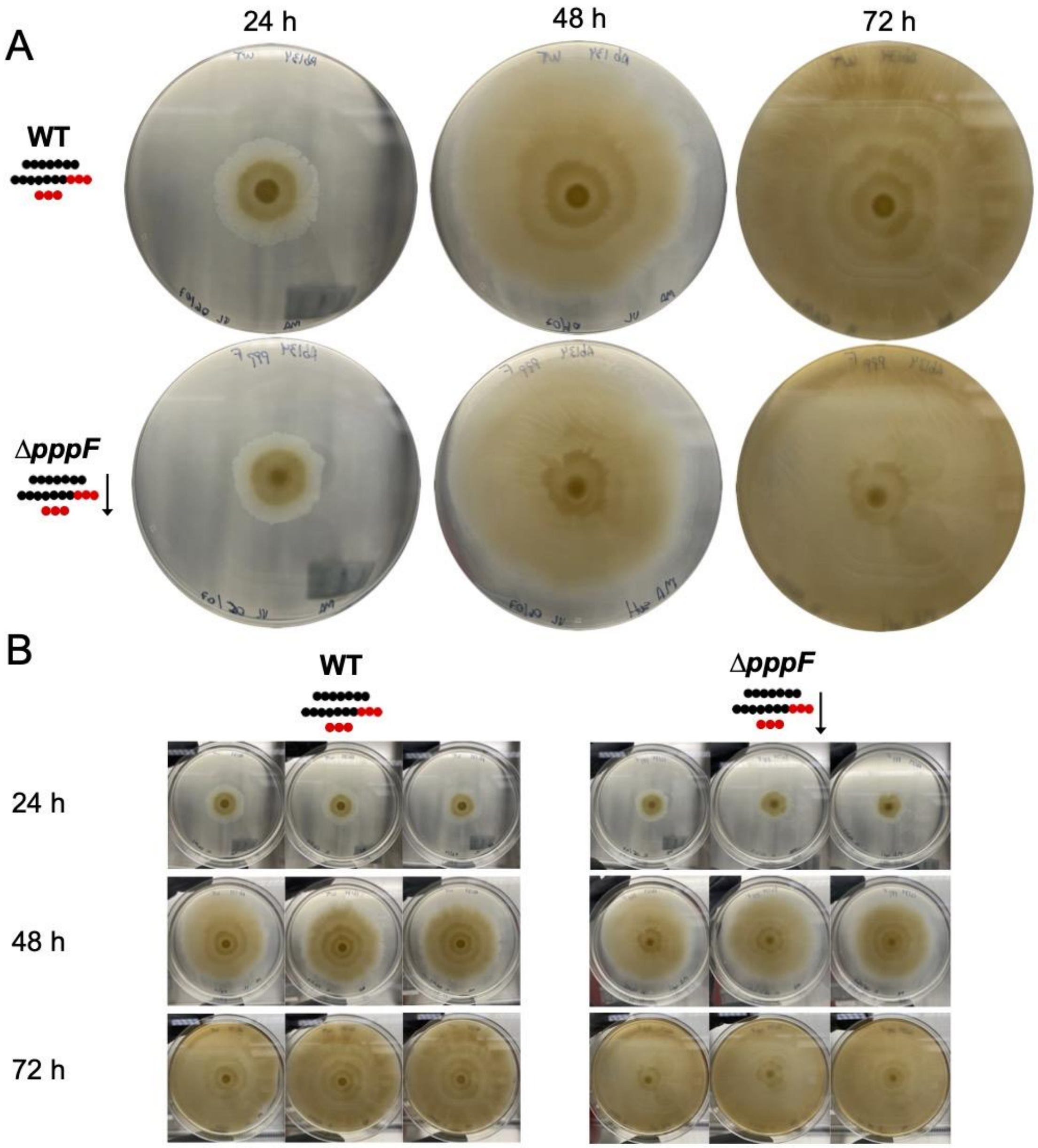

Figure S47. Swarming assay results of ∆*pppF* mutant compared to the wild type. The swarming assay was performed as described under Methods. Pictures shown were taken at 24, 48, and 72 hours after inoculation. (**A**) Representative results are shown. Pseudovibriamides are represented by beads: PA, seven black beads; PB, seven black beads and three red beads; PC, three red beads. (**B**) Triplicates of each strain are shown. The downward arrow means less pseudovibriamide produced.

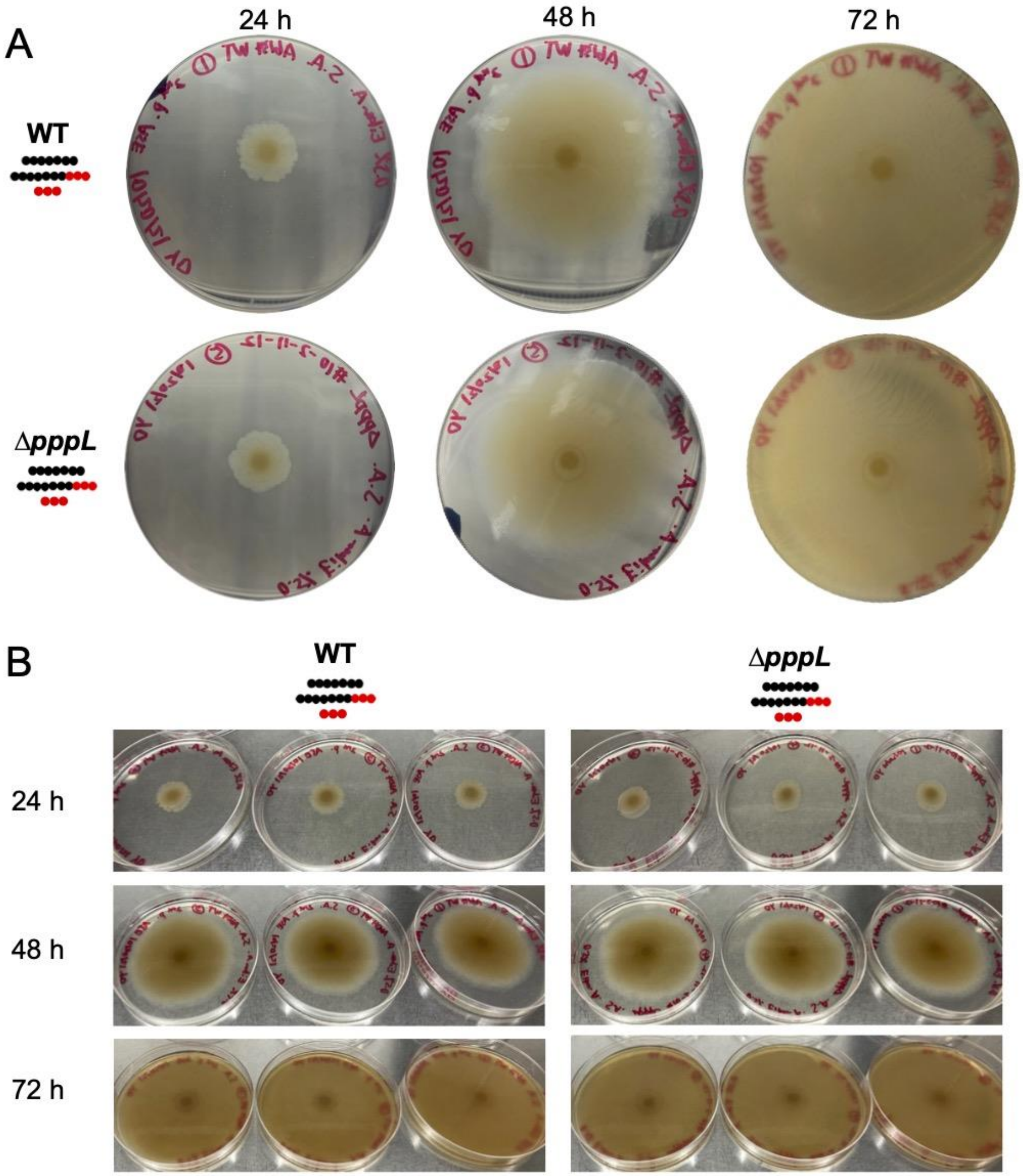

Figure S48. Swarming assay results of Δ*pppL* mutant compared to the wild type. The swarming assay was performed as described under Methods. Pictures shown were taken at 24, 48, and 72 hours after inoculation. (**A**) Representative results are shown. Pseudovibriamides are represented by beads: PA, seven black beads; PB, seven black beads and three red beads; PC, three red beads. (**B**) Triplicates of each strain are shown.

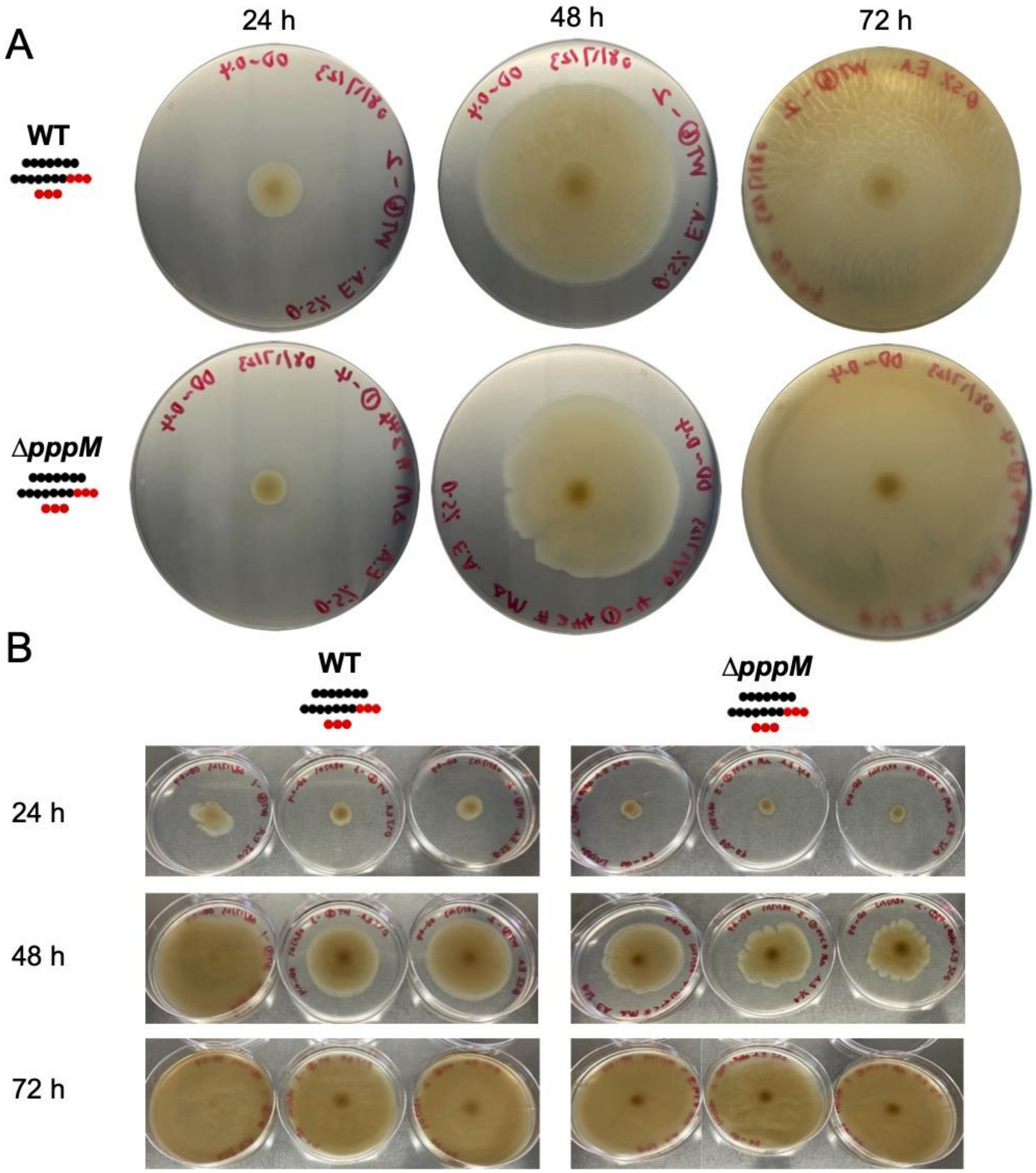

Figure S49. Swarming assay results of Δ*pppM* mutant compared to the wild type. The swarming assay was performed as described under Methods. Pictures shown were taken at 24, 48, and 72 hours after inoculation. (**A**) Representative results are shown. Pseudovibriamides are represented by beads: PA, seven black beads; PB, seven black beads and three red beads; PC, three red beads. (**B**) Triplicates of each strain are shown.

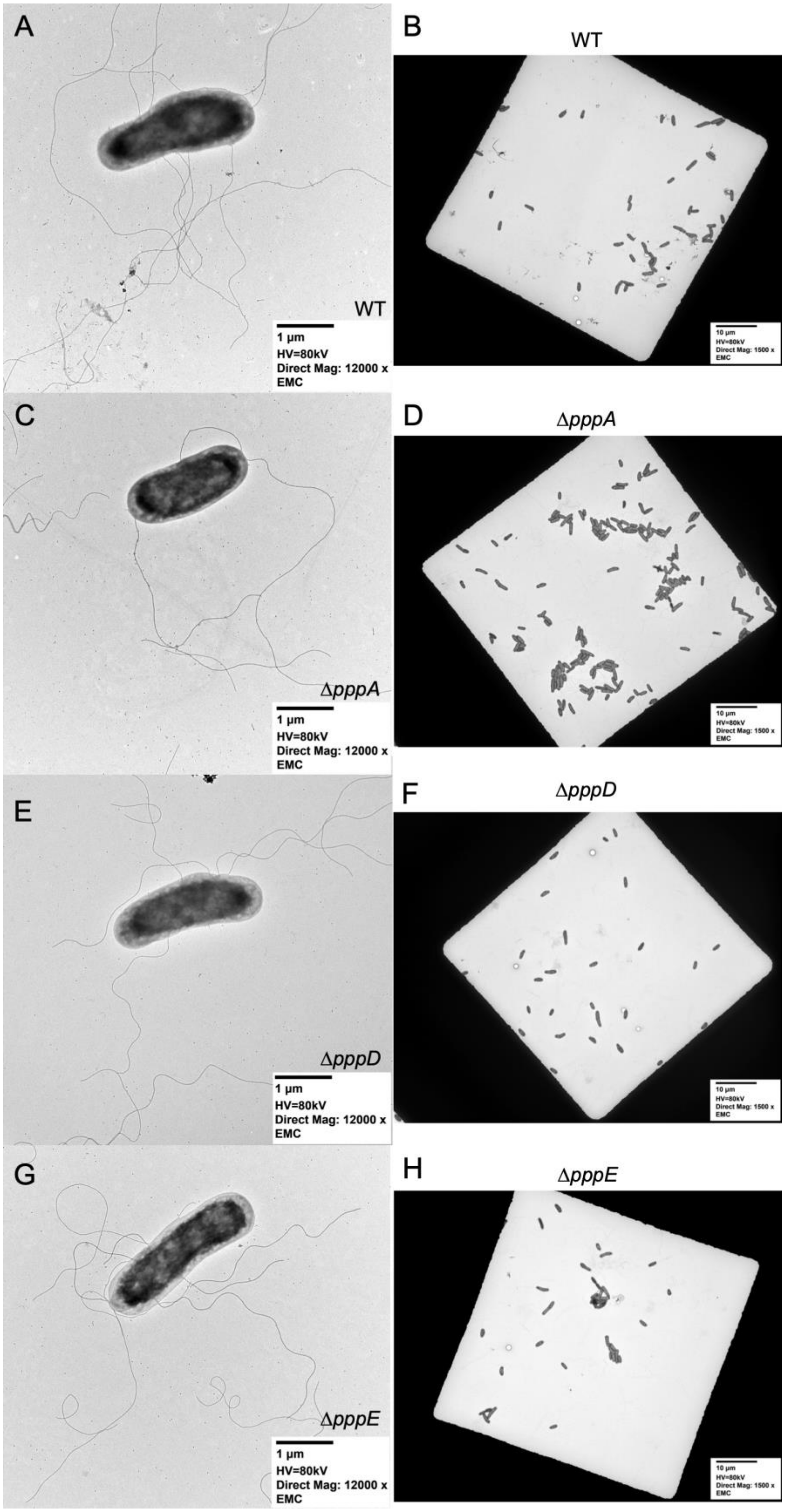
A
WT
1 µm
HV=80kV
Direct Mag: 12000 x
EMC
B
WT
C
ΔpppA
1 µm
HV=80kV
Direct Mag: 12000 x
EMC
D
ΔpppA
E
ΔpppD
1 µm
HV=80kV
Direct Mag: 12000 x
EMC
F
ΔpppD
G
ΔpppE
1 µm
HV=80kV
Direct Mag: 12000 x
EMC
H
ΔpppE

Figure S50. Transmission Electron Microscopy of wild type and mutants. Images of single cells and individual mesh windows, respectively of (**A**) and (**B**) wild type; (**C**) and (**D**) Δ*pppA* mutant; (**E**) and (**F**) Δ*pppD* mutant; (**G**) and (**H**) Δ*pppE* mutant. Each TEM grid has 300 mesh windows in total. Negative staining with 1% phosphotungstic acid was used. HV, high voltage; EMC, Electron Microscopy Core.

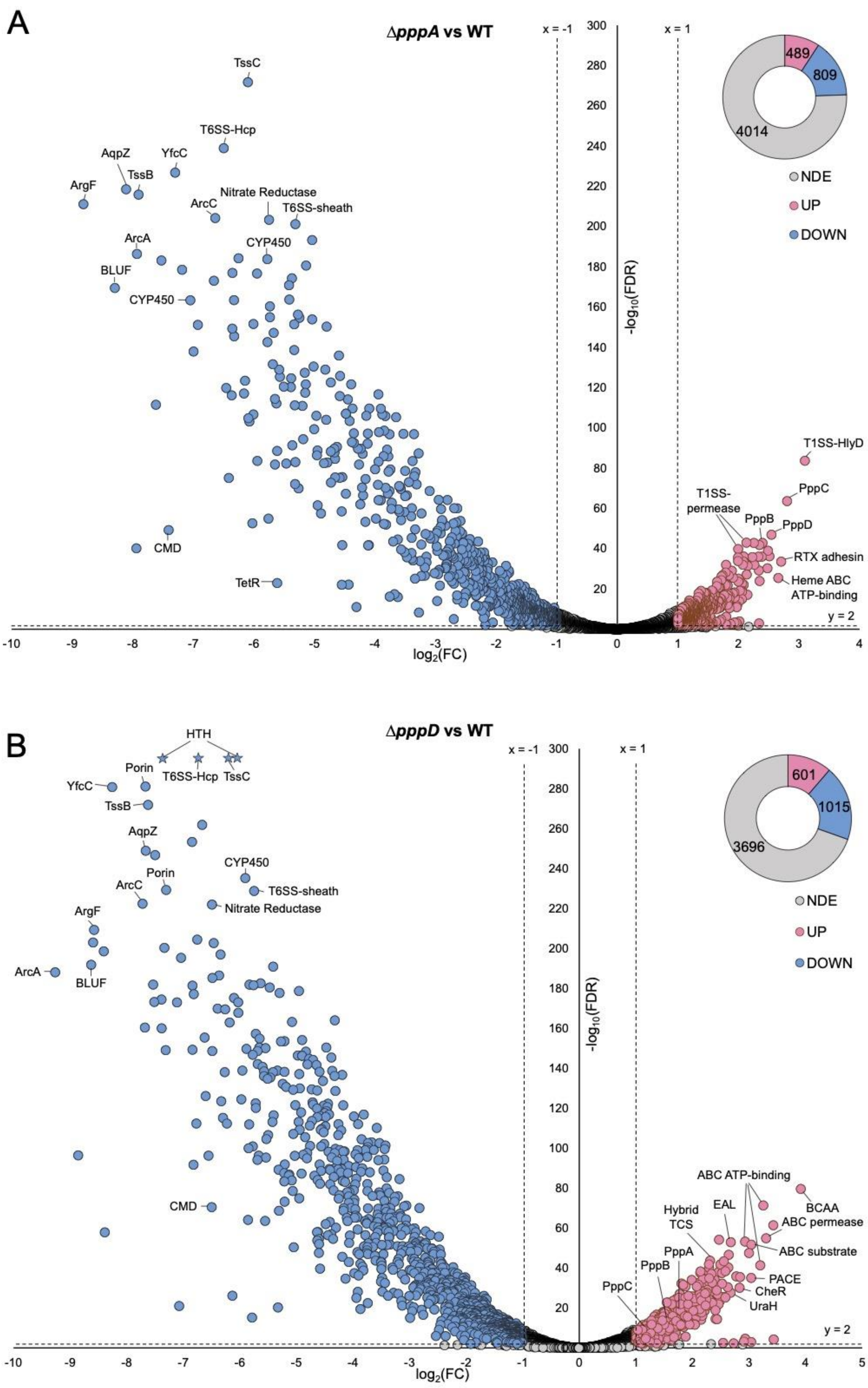
A
ΔpppA vs WT
x = -1
x = 1
y = 2
-log10(FDR)
log2(FC)
TssC
T6SS-Hcp
AqpZ
YfcC
TssB
ArgF
Nitrate Reductase
ArcC
T6SS-sheath
ArcA
CYP450
BLUF
CYP450
CMD
TetR
T1SS-HlyD
PppC
T1SS-permease
PppB
PppD
RTX adhesin
Heme ABC ATP-binding
489
809
4014
NDE
UP
DOWN
B
ΔpppD vs WT
HTH
T6SS-Hcp
TssC
Porin
YfcC
TssB
AqpZ
CYP450
Porin
ArcC
T6SS-sheath
Nitrate Reductase
ArgF
ArcA
BLUF
CMD
ABC ATP-binding
BCAA
EAL
Hybrid TCS
ABC permease
PppA
ABC substrate
PppB
PACE
PppC
CheR
UraH
601
1015
3696
NDE
UP
DOWN

Figure S51. Volcano plots of differentially expressed genes identified between Δ*pppA* and Δ*ppp*D mutants and the wild type using DESeq2. (**A**) Differentially expressed genes of Δ*pppA* mutant compared to wild type. (**B**) Differentially expressed genes of Δ*pppD* mutant compared to wild type. FDR, false discovery rate or q-value; FC, fold-change; NDE, non-differential expression; UP, upregulated; DOWN, downregulated; Blue dots or blue donut portion, downregulated genes; Pink dots or pink donut portion, upregulated genes; and grey dots or grey donut portion, non-differentially expressed genes. The same filter (dotted lines) was applied to all differential expression analyses that is FC ≤ -2 (x = -1) or FC ≥ 2 (x = 1), and FDR ≤ 1% (y = 2). The total number of genes from each portion is listed in the donut chart. Products of outstanding downregulated or upregulated genes are labeled. HTH, helix-turn-helix transcriptional regulator; TssB, type VI secretion system contractile sheath small subunit; TssC, type VI secretion system contractile sheath large subunit; T6SS-sheath, type VI secretion system contractile sheath large subunit; T6SS-Hcp, type VI secretion system tube protein Hcp; YfcC, Arginine/ornithine antiporter; AqpZ, aquaporin Z; ArcA, arginine deiminase; ArcC, carbamate kinase; ArgF, ornithine carbamoyltransferase; BLUF, blue light using flavin domain; CYP450, cytochrome P450; T1SS-HlyD, HlyD family type I secretion periplasmic adaptor subunit; RTX adhesin, repeats-in-toxin adhesion protein; T1SS permease, type I secretion system permease/ATPase; CMD, carboxymuconolactone decarboxylase; TetR, TetR/AcrR family transcriptional regulator; BCAA ABC permease, branched-chain amino acid ATP-binding cassette transporter permease; ABC ATP-binding, ATP-binding cassette transporter ATP-binding protein; ABC substrate, ATP-binding cassette transporter substrate binding protein; Heme ABC ATP-binding, heme ATP-binding cassette transporter ATP-binding protein; EAL, EAL domain containing protein; Hybrid TCS, hybrid sensor histidine kinase/response regulator; PACE, the proteobacterial antimicrobial compound efflux transporter; CheR, protein-glutamate O-methyltransferase; and UraH, hydroxyisourate hydrolase. Some outstanding dots are unlabeled as they are hypothetical

proteins. Stars represent DE genes with too large values of -log10(FDR), which were rounded to 1000.

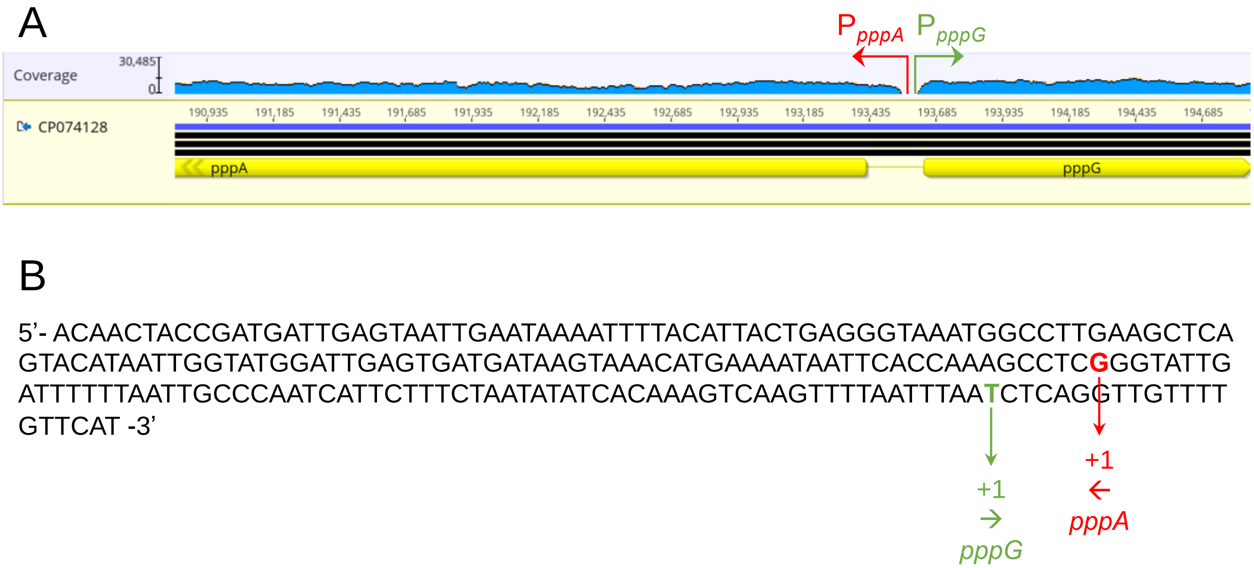

Figure S52. Identification of P_{pppA} based on RNA-Seq data. (**A**) RNA-Seq reads mapped to the reference genome. The gap in coverage indicates the presence of divergent promoters for *pppA* and *pppG*. (**B**) Sequence of promoter region highlighting the predicted +1 of transcription of *pppG* and +1 of transcription of *pppA* according to RNA-seq data.

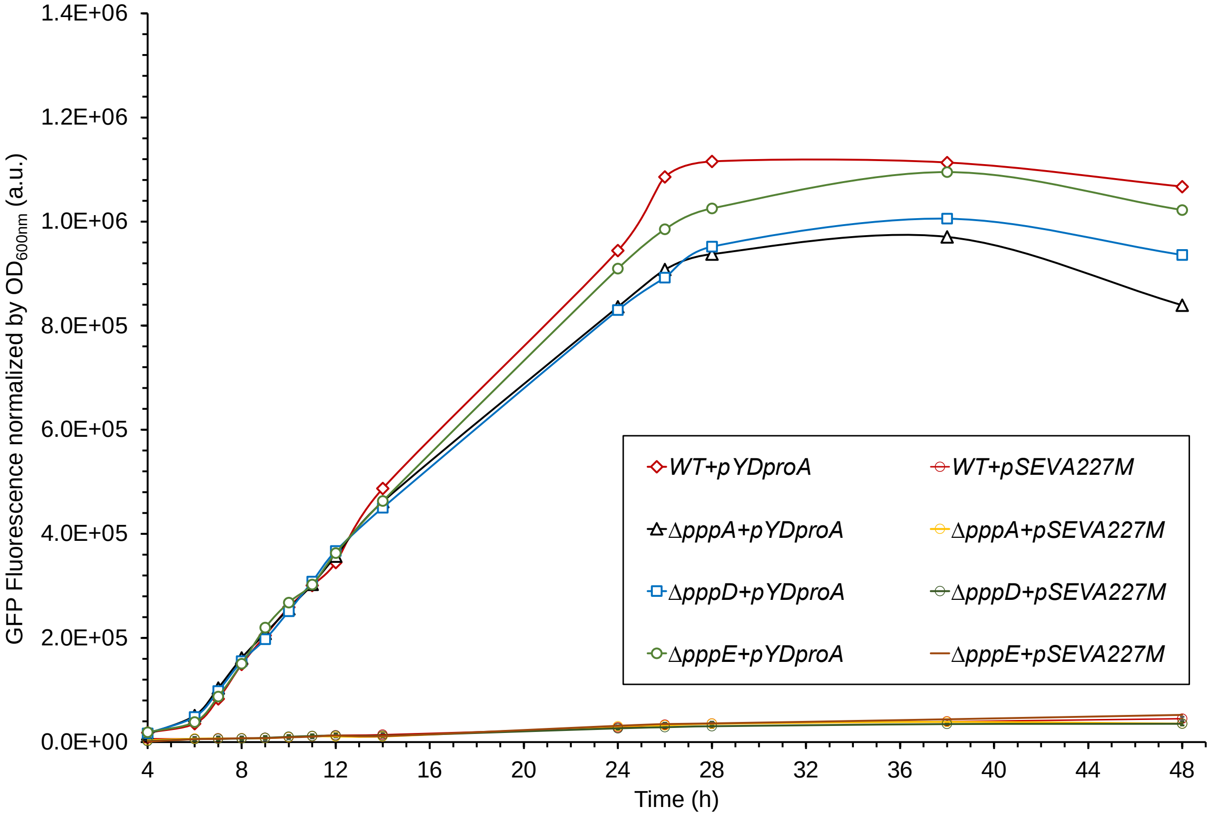

Figure S53. P*pppA* autoregulation assay. Readings of GFP fluorescence level were normalized by OD_{600nm} and plotted against incubation time of 48 hours. pYDproA, GFP under P_{pppA}; pSEVA227M, promoterless GFP. *N*=12. Error bars indicate standard deviation.

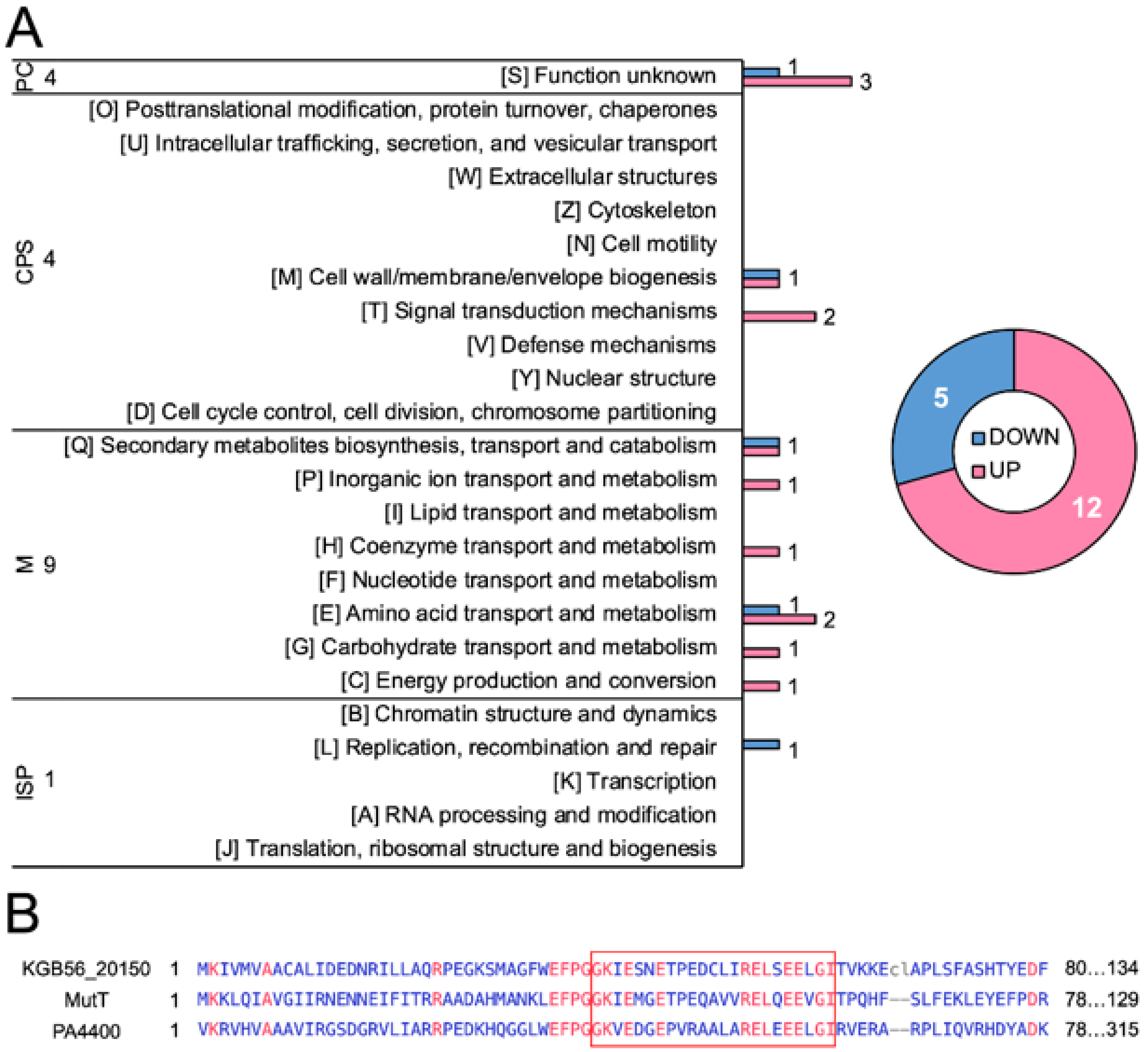

Figure S54. COG classification of DE genes in Δ*pppA/E* mutants filtered using NDE genes of Δ*pppD* mutant compared to the wild type. (**A**) The distribution of 24 upregulated genes and 5 downregulated genes in each COG category. The donut graph shows the total number of upregulated or downregulated genes. Pink, upregulated; Blue, downregulated. PC, poorly characterized; CPS, cellular processes and signaling; M, metabolism; and ISP, information storage and processing. The total number of genes in each category is listed. (**B**) COBALT was used to align amino acid sequences using Ab134 MutT as the anchor sequence. *P. brasiliensis*

Ab134 KGB56_20150 (QUS55589.1); *E. coli* strain K12 MutT (CAD6022180.1); and *P. aeruginosa* PAO1 PA4400 (NP_253090.1). Red color denotes conserved residues. Blue color denotes not conserved residues. The red box highlights the conserved Nudix motif, G-X5-E-X7-R-E-U-X-E-E-X-G-U, where X could be any amino acid, and U should be a hydrophobic amino acid.

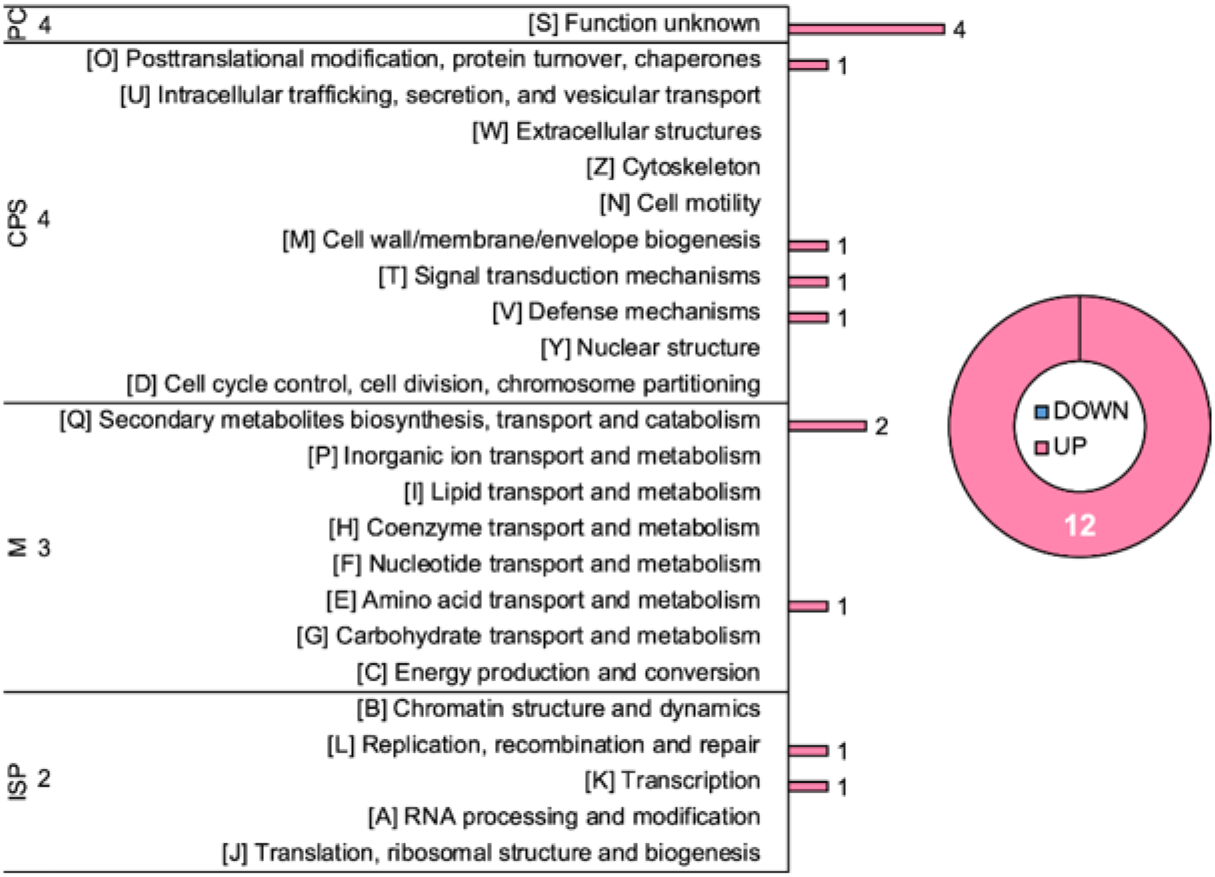

Figure S55. COG classification of DE genes reversely regulated in Δ*pppA/E* mutants and the Δ*pppD* mutant compared to the wild type. Donut graph shows the total number of upregulated or downregulated genes. Pink color, upregulated; Blue color, downregulated; PC, poorly characterized; CPS, cellular processes and signaling; M, metabolism; and ISP, information storage and processing. The total number of genes in each category is listed.

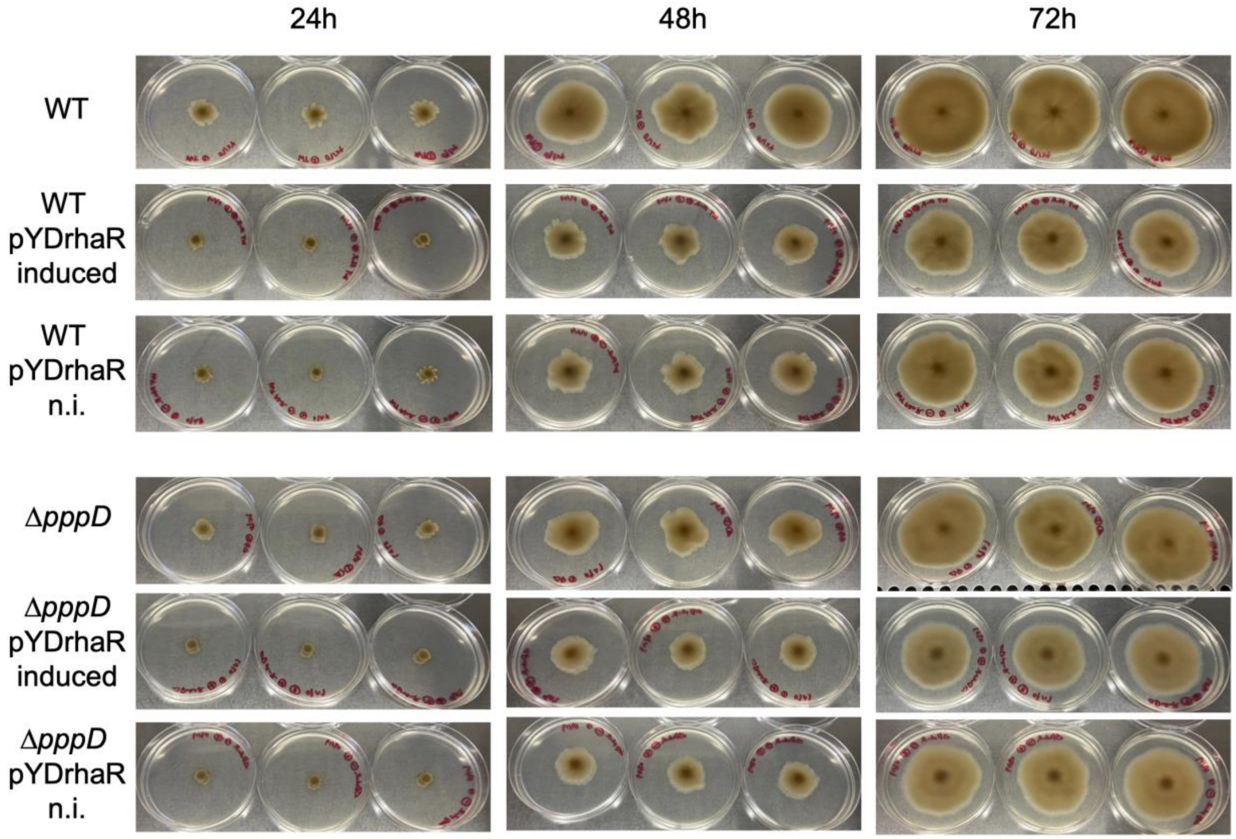

Figure S56. Swarming assay results of strains with inducible vector pYDrhaR. The wild type (WT), WT with pYDrhaR induced or not induced (n.i.), the Δ*pppD* mutant, and the Δ*pppD* mutant with pYDrhaR induced or not induced are shown. The swarming assay was performed as described under Methods. Pictures shown were taken at 24, 48, and 72 hours after inoculation. Induced plates contained 0.1% L-rhamnose. No kanamycin was added.

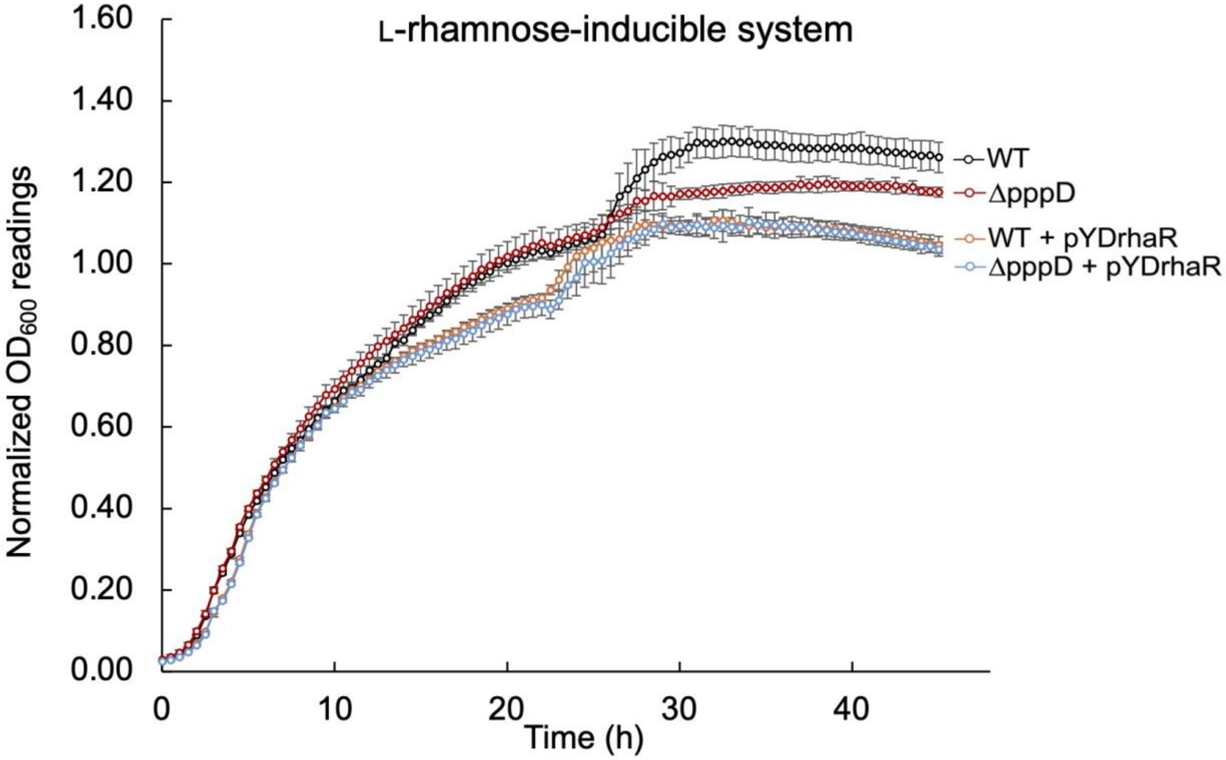

Figure S57. Growth of strains with inducible vector pYDrhaR as measured by OD_{600}. The growth of the wild type (WT), WT + pYDrhaR, the Δ*pppD* mutant, and Δ*pppD* + pYDrhaR for 46 hours is shown. *N*=10. Error bars indicate standard deviation. No L-rhamnose was added.

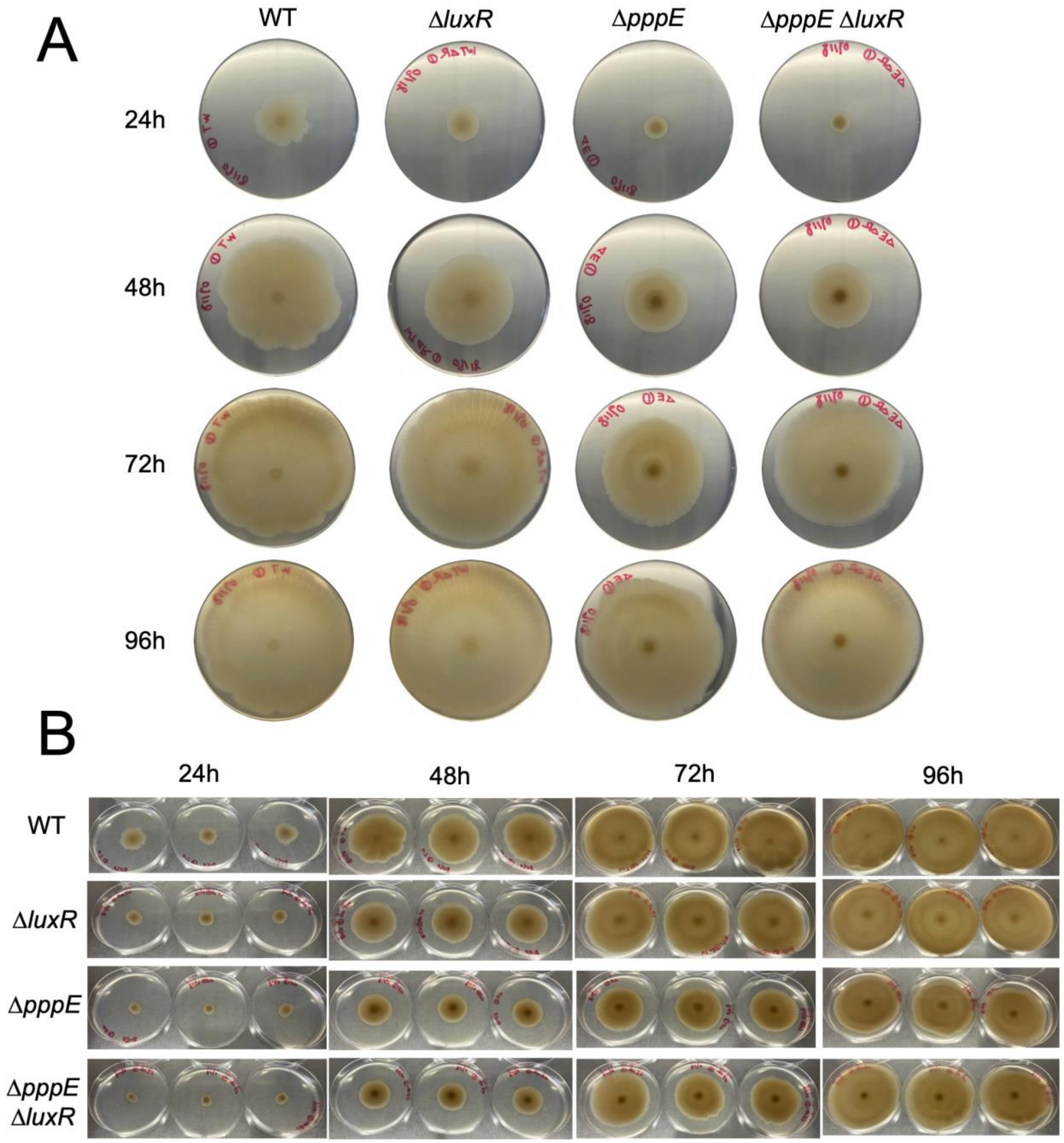

Figure S58. Swarming assay results of the wild type, Δ*luxR*, Δ*pppE*, and Δ*pppE* Δ*luxR* mutants. The swarming assay was performed as described under Methods. Pictures shown were taken at 24, 48, 72, and 96 hours after inoculation. (**A**) Representative results are shown. (**B**) Triplicates of each strain are shown.

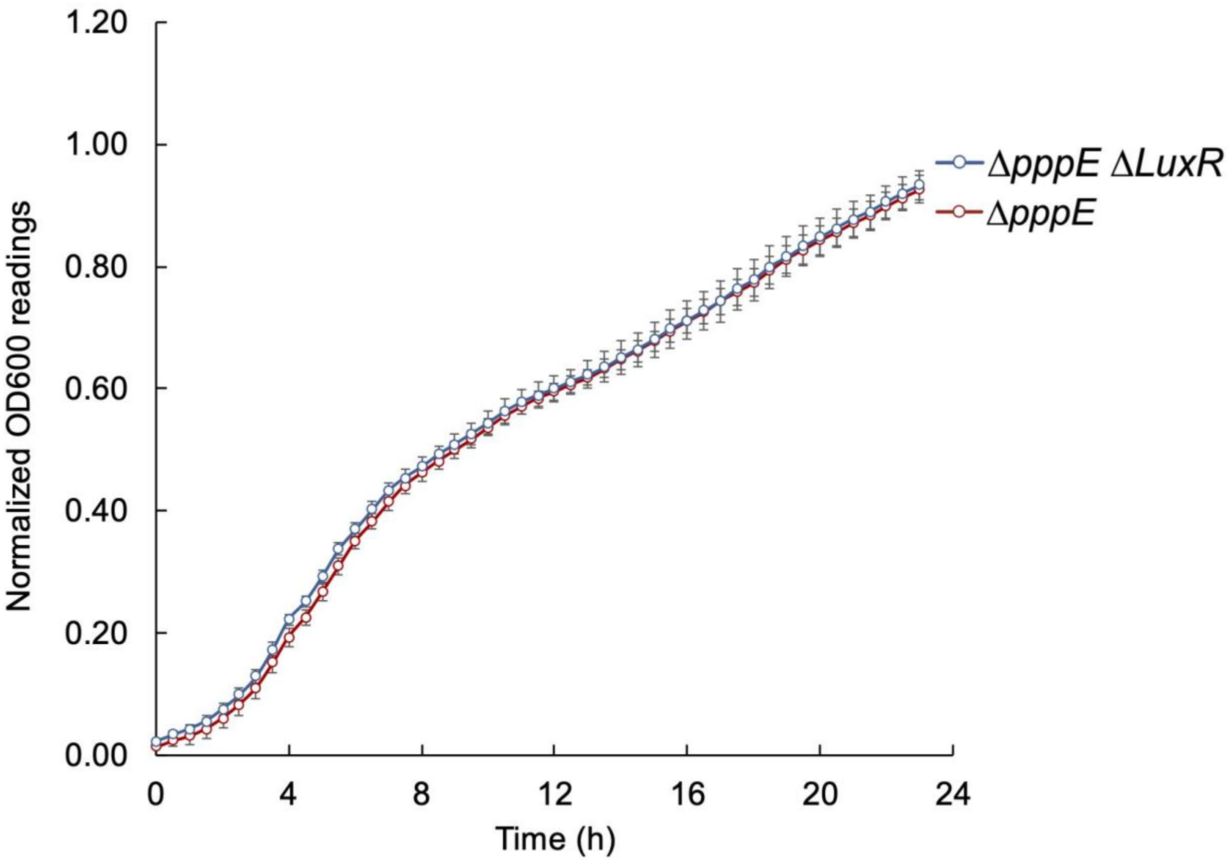

Figure S59. Growth of the ∆*pppE* mutant, and the ∆*pppE* ∆*luxR* mutant as measured by OD_{600} for 24 hours. *N*=10. Error bars indicate standard deviation.

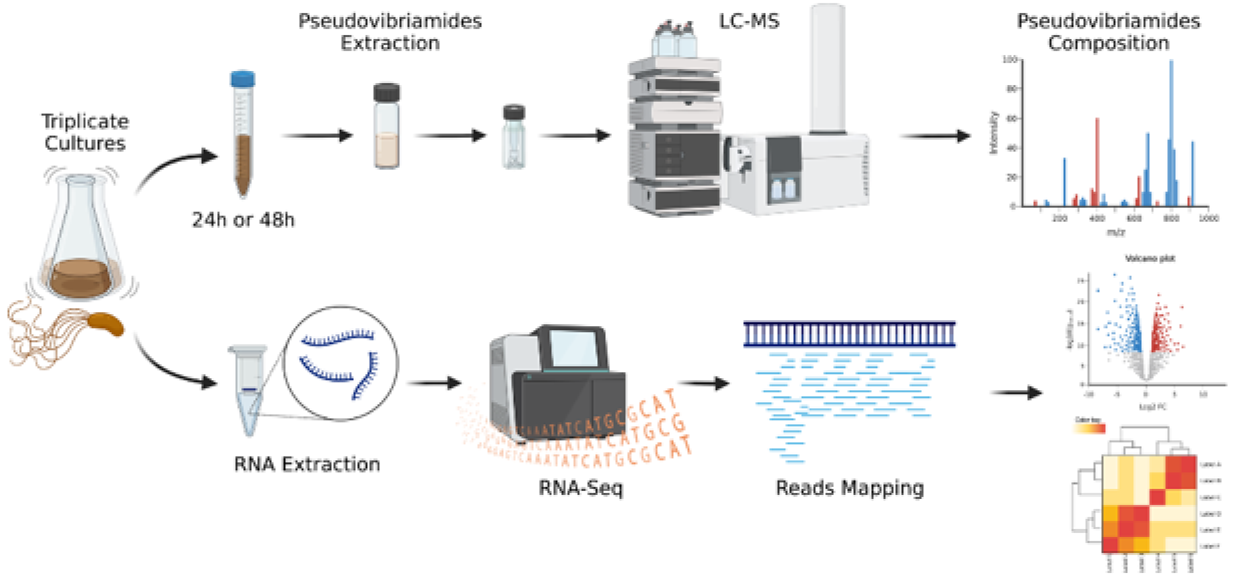

Figure S60. Workflow from bacterial culture to transcriptomic analysis.

References

1. Kudryashev M, Stenta M, Schmelz S, Amstutz M, Wiesand U, Castaño-Díez D, Degiacomi MT, Münnich S, Bleck CK, Kowal J, Diepold A, Heinz DW, Dal Peraro M, Cornelis GR, Stahlberg H. 2013. In situ structural analysis of the *Yersinia enterocolitica* injectisome. Elife 2:e00792.

2. Tremblay J, Déziel E. 2010. Gene expression in *Pseudomonas aeruginosa* swarming motility. BMC Genomics 11:587.

3. Ióca LP, Dai Y, Kunakom S, Diaz-Espinosa J, Krunic A, Crnkovic CM, Orjala J, Sanchez LM, Ferreira AG, Berlinck RGS, Eustáquio AS. 2021. A family of nonribosomal peptides modulate collective behavior in *Pseudovibrio* bacteria isolated from marine sponges. Angew Chem Int Ed Engl 60:15891–15898.

4. Süssmuth RD, Mainz A. 2017. Nonribosomal peptide synthesis—principles and prospects. Angew Chem Int Ed 56:3770–3821.

5. Fischbach MA, Walsh CT. 2006. Assembly-line enzymology for polyketide and nonribosomal peptide antibiotics: logic, machinery, and mechanisms. Chem Rev 106:3468–3496.

6. Beld J, Sonnenschein EC, Vickery CR, Noel JP, Burkart MD. 2014. The phosphopantetheinyl transferases: catalysis of a post-translational modification crucial for life. Nat Prod Rep 31:61–108.

7. Heathcote ML, Staunton J, Leadlay PF. 2001. Role of type II thioesterases: evidence for removal of short acyl chains produced by aberrant decarboxylation of chain extender units. Chem Biol 8:207–220.

8. Schwarzer D, Mootz HD, Linne U, Marahiel MA. 2002. Regeneration of misprimed nonribosomal peptide synthetases by type II thioesterases. Proc Natl Acad Sci U S A 99:14083–14088.

9. Pao SS, Paulsen IT, Saier MH. 1998. Major facilitator superfamily. Microbiol Mol Biol Rev 62:1–34.

10. Anderson AC, Stangherlin S, Pimentel KN, Weadge JT, Clarke AJ. 2022. The SGNH hydrolase family: a template for carbohydrate diversity. Glycobiology 32:826-848.

11. Baker P, Ricer T, Moynihan PJ, Kitova EN, Walvoort MTC, Little DJ, Whitney JC, Dawson K, Weadge JT, Robinson H, Ohman DE, Codée JDC, Klassen JS, Clarke AJ, Howell PL. 2014. *P. aeruginosa* SGNH hydrolase-like proteins AlgJ and AlgX have similar topology but separate and distinct roles in alginate acetylation. PLoS Pathog 10:e1004334.

12. Martinez S, Hausinger RP. 2015. Catalytic mechanisms of Fe(II)- and 2-Oxoglutarate-dependent oxygenases. J Biol Chem 290:20702–20711.

13. Kumar S, Mukherjee MM, Varela MF. 2013. Modulation of bacterial multidrug resistance efflux pumps of the major facilitator superfamily. Int J Bacteriol 2013:204141.

14. Burckhardt RM, Escalante-Semerena JC. 2020. Small-molecule acetylation by GCN5-related *N*-acetyltransferases in bacteria. Microbiol and Mol Biol Rev 84:e00090-19.

15. Maqbool A, Horler RSP, Muller A, Wilkinson AJ, Wilson KS, Thomas GH. 2015. The substrate-binding protein in bacterial ABC transporters: dissecting roles in the evolution of substrate specificity. Biochem Soc Trans 43:1011–1017.

16. Mitrophanov AY, Groisman EA. 2008. Signal integration in bacterial two-component regulatory systems. Genes Dev 22:2601–2611.

17. Gao R, Mack TR, Stock AM. 2007. Bacterial response regulators: versatile regulatory strategies from common domains. Trends Biochem Sci 32:225–234.

18. Philippe N, Alcaraz JP, Coursange E, Geiselmann J, Schneider D. 2004. Improvement of pCVD442, a suicide plasmid for gene allele exchange in bacteria. Plasmid 51:246–255.

19. Bierman M, Logan R, O'Brien K, Seno ET, Rao RN, Schoner BE. 1992. Plasmid cloning vectors for the conjugal transfer of DNA from *Escherichia coli* to *Streptomyces* spp. Gene 116:43–49.

20. Silva-Rocha R, Martínez-García E, Calles B, Chavarría M, Arce-Rodríguez A, de Las Heras A, Páez-Espino AD, Durante-Rodríguez G, Kim J, Nikel PI, Platero R, de Lorenzo V. 2013. The Standard European Vector Architecture (SEVA): a coherent platform for the analysis and deployment of complex prokaryotic phenotypes. Nucleic Acids Res 41:D666-675.

21. Taton A, Unglaub F, Wright NE, Zeng WY, Paz-Yepes J, Brahamsha B, Palenik B, Peterson TC, Haerizadeh F, Golden SS, Golden JW. 2014. Broad-host-range vector system for synthetic biology and biotechnology in Cyanobacteria. Nucleic Acids Res 42:e136.

22. Sydow A, Pannek A, Krieg T, Huth I, Guillouet SE, Holtmann D. 2017. Expanding the genetic tool box for *Cupriavidus necator* by a stabilized L-rhamnose inducible plasmid system. J Biotechnol 263:1–10.

23. Stachelhaus T, Mootz HD, Marahiel MA. 1999. The specificity-conferring code of adenylation domains in nonribosomal peptide synthetases. Chem Biol 6:493–505.
